# Heterologous protection against malaria by a simple chemoattenuated PfSPZ vaccine regimen in a randomized trial

**DOI:** 10.1038/s41467-021-22740-w

**Published:** 2021-05-04

**Authors:** Zita Sulyok, Rolf Fendel, Bianca Eder, Freia-Raphaella Lorenz, Natasha KC, Matthias Karnahl, Albert Lalremruata, The T. Nguyen, Jana Held, Folashade Almeine Cyntiche Adjadi, Torsten Klockenbring, Judith Flügge, Tamirat Gebru Woldearegai, Carlos Lamsfus Calle, Javier Ibáñez, Miriam Rodi, Diane Egger-Adam, Andrea Kreidenweiss, Carsten Köhler, Meral Esen, Mihály Sulyok, Anita Manoj, Thomas L. Richie, B. Kim Lee Sim, Stephen L. Hoffman, Benjamin Mordmüller, Peter G. Kremsner

**Affiliations:** 1 Institute of Tropical Medicine, University and University Hospital of Tübingen, Tübingen, Germany; 2 German Center for Infection Research (DZIF), Partner Site Tübingen, Tübingen, Germany; 3 Sanaria Inc, Rockville, MD USA; 4 Fraunhofer Institute of Molecular Biology and Applied Ecology IME, Aachen, Germany; 5 Department of Medical Microbiology, Radboud University Medical Center, Nijmegen, The Netherlands; 6 Centre de Recherches Médicales de Lambaréné (CERMEL), Lambaréné, Gabon

**Keywords:** Malaria, Phase I trials, Translational research

## Abstract

Immunization with *Plasmodium falciparum* (Pf) sporozoites under chemoprophylaxis (PfSPZ-CVac) is the most efficacious approach to malaria vaccination. Implementation is hampered by a complex chemoprophylaxis regimen and missing evidence for efficacy against heterologous infection. We report the results of a double-blinded, randomized, placebo-controlled trial of a simplified, condensed immunization regimen in malaria-naive volunteers (EudraCT-Nr: 2018-004523-36). Participants are immunized by direct venous inoculation of 1.1 × 10^5^ aseptic, purified, cryopreserved PfSPZ (PfSPZ Challenge) of the PfNF54 strain or normal saline (placebo) on days 1, 6 and 29, with simultaneous oral administration of 10 mg/kg chloroquine base. Primary endpoints are vaccine efficacy tested by controlled human malaria infection (CHMI) using the highly divergent, heterologous strain Pf7G8 and safety. Twelve weeks following immunization, 10/13 participants in the vaccine group are sterilely protected against heterologous CHMI, while (5/5) participants receiving placebo develop parasitemia (risk difference: 77%, p = 0.004, Boschloo’s test). Immunization is well tolerated with self-limiting grade 1–2 headaches, pyrexia and fatigue that diminish with each vaccination. Immunization induces 18-fold higher anti-Pf circumsporozoite protein (PfCSP) antibody levels in protected than in unprotected vaccinees (p = 0.028). In addition anti-PfMSP2 antibodies are strongly protection-associated by protein microarray assessment. This PfSPZ-CVac regimen is highly efficacious, simple, safe, well tolerated and highly immunogenic.

## Abstract (web summary)

In this placebo-controlled trial, 10/13 malaria naïve subjects immunized with a simplified regimen of chemoattenuated *P. falciparum* sporozoites, PfSPZ-CVac, show sterile protection from heterologous malaria challenge. Immunization was well tolerated and induced high levels of anti-PfCSP antibodies.

## Introduction

The global disease burden of malaria is a major public health challenge. The World Health Organization (WHO) estimated a total of 228 million cases and 405,000 deaths in 2018 worldwide; the majority caused by *Plasmodium falciparum* (Pf) in sub-Saharan Africa^1^. Despite the decreasing incidence in many countries, the economic and social consequences of malaria are still enormous. New interventions for prevention and treatment are critically needed to control and eradicate the disease. An effective vaccine would be an ideal additional tool to reach this goal. However, developing a vaccine against parasites is particularly challenging because of their complexity in genome size, life cycle, epidemiology, and immunology. The only vaccine against malaria that has completed clinical development is RTS,S, a recombinant protein vaccine targeting the Pf circumsporozoite protein (PfCSP), the predominant sporozoite surface protein. It received a positive scientific opinion from the European Medicines Agency in 2015 but has not received marketing approval (licensure) so far due to moderate vaccine efficacy (VE) and inconclusive safety signals^2,3^.

Whole-cell Pf sporozoite (PfSPZ)-based vaccines are a promising way to evoke immunity, since a broad antigenic repertoire of the parasite is present in the pre-erythrocytic development stages, especially in the liver phase.

The history of attempts in humans to develop such a vaccine dates back to the 1970s^4,5^. The translation of experimental immunization using mosquito bites into a candidate vaccine was only recently achieved by developing aseptic, purified, cryopreserved PfSPZ for use in humans^6^. Availability of PfSPZ products boosted the development of malaria vaccines. It was shown in previous trials that immunization with whole-cell sporozoites, either radiation-attenuated (PfSPZ Vaccine)^7–11^ or chemoattenuated by the concomitant administration of an antimalarial (PfSPZ chemoprophylaxis vaccine, PfSPZ-CVac)^12–14^, is highly immunogenic and induces robust protection against homologous (vaccine) strain-controlled human malaria infection (CHMI). However, after mosquito bite immunization with this approach, which is called chemoprophylaxis with sporozoites (CPS), VE against heterologous (non-vaccine) strain CHMI was minimal^15,16^.

In our previous PfSPZ-CVac trial TÜCHMI-002 (NCT02115516), we showed that direct venous inoculation (DVI) administration of three doses of 5.12 × 10^4^ PfSPZ Challenge (infectious PfSPZ) over 8 weeks under weekly chloroquine (CQ) chemoprophylaxis protected 100% (9/9) of study participants against CHMI conducted with the homologous (vaccine) strain of Pf and performed 10 weeks after immunization^12^. CQ, a blood schizonticide without effect on liver stages, was selected as the partner drug rather than a liver active drug to allow parasite multiplication within hepatocytes, thereby increasing the antigenic stimulus and the expression of late liver stage and early blood stage antigens. However, this regimen was suboptimal for a routine setting as it required 13 clinic visits including ten for administration of CQ. In the second cohort of this trial, a condensed immunization regimen requiring fewer doses of CQ was selected. Here, three doses of 5.12 × 10^4^ PfSPZ Challenge at 5- or 14-day intervals protected 63% (5/8) and 67% (6/9) of volunteers, respectively^12^. These results indicated that shorter regimens with fewer doses of CQ could be used, although potentially at the cost of reduced VE.

**Fig. 1:**
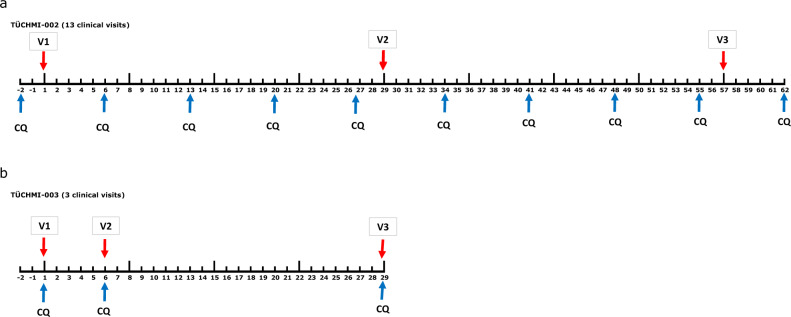
Diagram illustrating the differences between the established and current vaccination regimen for PfSPZ-CVac (CQ). **a** The previously used vaccination regimen included 13 clinic visits during 9 weeks, 10 for administration of CQ, and 3 for immunizations^12^. **b** The immunization regimen assessed in this study included three clinic visits during 4 weeks, three for administration of CQ, and the same three for immunizations. Red arrows: vaccination time points; blue arrows: chloroquine treatment time points.

Building on these data, the aim of the current study was to establish a condensed immunization regimen of three injections of PfSPZ Challenge offering improved VE compared to the prior condensed regimens. To compensate for the potentially reduced VE due to the condensed regimen and heterologous CHMI, the dose was increased from the previously administered dose (5.12 × 10^4^ PfSPZ Challenge)^12^ to a dose of 1.1 × 10^5^ PfSPZ Challenge per injection. To facilitate the procedure for future application (e.g. in endemic countries and for travelers) CQ was given only on the days of PfSPZ Challenge injection, thereby requiring only three visits to complete vaccination (Fig. 1). Additionally, we aimed to determine if a condensed regimen with increased dose of PfSPZ Challenge protected against heterologous CHMI.

## Results

### Description of study population

**Fig. 2:**
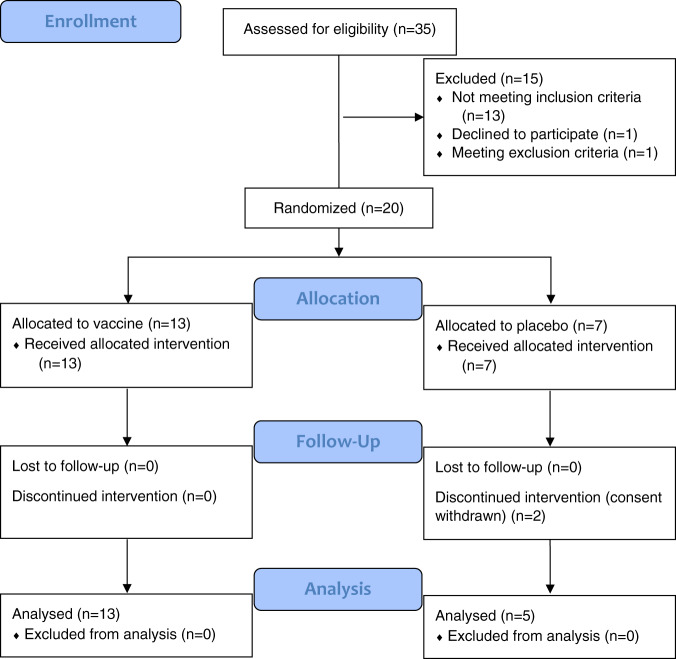
Study flow chart. CONSORT flow diagram showing study participant flow through for each individual stage of the randomized controlled vaccination trial (enrollment, allocation, follow-up, and analysis).

We enrolled 21 volunteers from 30 April 2019 to 15 May 2019. One participant dropped out due to a new onset ECG abnormality on Day 1 before any study treatment was given. Hence, 20 volunteers were randomized and received at least one immunization (intention to treat population). A total of 18 participants underwent all three immunizations and CHMI (per protocol population). Two volunteers withdrew consent after the second immunization and received rescue treatment with atovaquone/proguanil on Days 34, 35, and 36 (later identified as placebo recipients). Third immunization was postponed in the case of one volunteer (to Day 35 instead of Day 28) because of an acute gastroenteritis (later identified as placebo recipient). Baseline characteristics and study flow chart are shown in Table 1 and Fig. 2.

**Table 1:** Demographic data.

Number of participants (*n* = 20)	Vaccine	Placebo	*P* value
Sex			
Male (*n,* percentage)	6 (46%)	5 (71%)	*p* = 0.37*
Female (*n*, percentage)	7 (54%)	2 (29%)	
Age in years (median, range)	25 (19–42)	26 (21–36)	*p* = 0.79**
Height in cm (median, range)	171 (159–184)	178 (165–188)	*p* = 0.14**
Weight in kg (median, range)	69 (50–100)	73 (51–86)	*p* = 0.92**
BMI in kg/m^2^ (median, range)	23.8 (16.9–33.8)	23.2 (18.7–25.6)	*p* = 0.50**

^*^Two-sided Fisher’s exact test.

^**^Two-sided Student’s *t*-test.

### Parasitemia during immunization

**Fig. 3:**
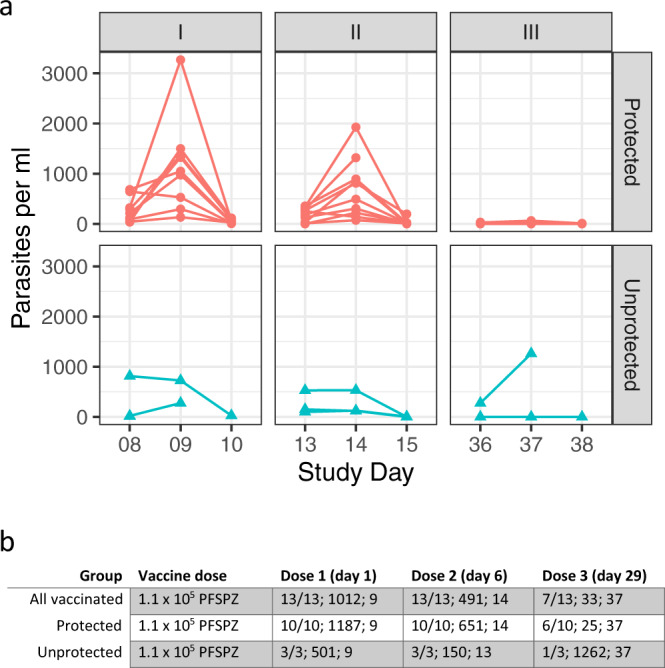
Parasitemia during immunization phase. **a** Parasitemia (parasites/ml) was estimated by quantitative PCR during the immunization phase after immunizations 1, 2, and 3. Individual parasitemia levels are shown. Red circles: vaccinated group (protected); turquoise triangles: vaccinated group (unprotected). During the first vaccination period, one unprotected vaccinee missed visits of Study days 9 and 10 after immunization, two volunteers missed the visit on Study day 10 after immunization. At second immunization, one protected volunteer missed the visit on Study day 15. For the third immunization, one unprotected and one protected vaccinee missed the visit on Study day 38 after immunization. **b** Number of subjects positive per number injected, median peak parasite density among positives, and study day of median peak parasite density among positives after each dose of PfSPZ-CVac. Values are given for all vaccinated volunteers as well as separated by outcome of CHMI.

We had previously shown that transient parasitemia develops 7 days after administration of 5.12 × 10^4^ PfSPZ Challenge (PfNF54) during immunization and the peak parasite density diminishes after each of three immunizations^12^. In this study, after administration of 1.1 × 10^5^ PfSPZ Challenge (PfNF54) parasite density peaked 8 days after each immunization, but never reached the levels seen with half the dose of immunizing PfSPZ in our first study (median peak parasite density after first dose was 15,755 vs 1012 parasites/ml in this study) (Fig. 3). In the previous study 7/9 (78%) subjects had detectable parasitemia after the third immunization^12^, but in this study only 7 of the 13 vaccinees (54%) had any detectable parasitemia (Fig. 3).

### Vaccine efficacy

**Fig. 4:**
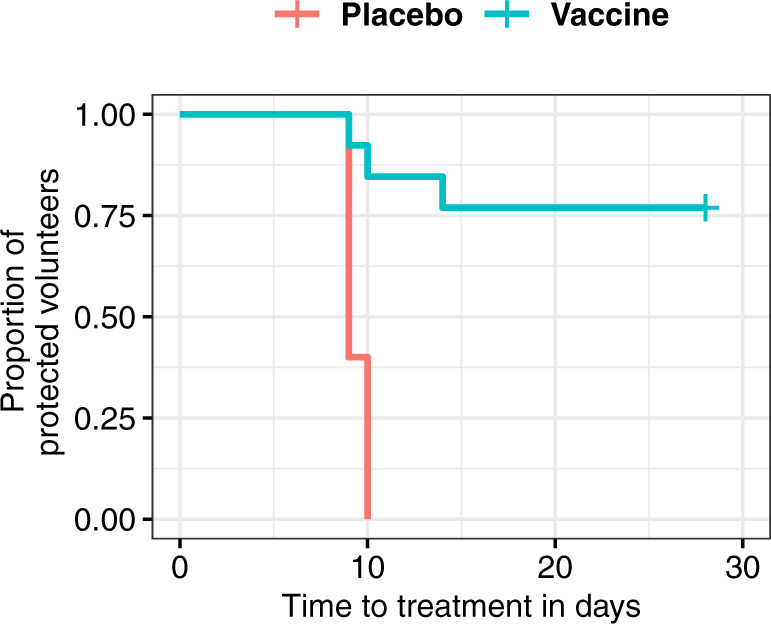
Kaplan–Meier plot of time from infection to treatment initiation. The overall time to treatment (in days) after injection of 3.2 × 10^3^ PfSPZ Challenge (onset of CHMI) is presented for both the placebo (*n* = 5) and the vaccinated (*n* = 13) groups. The cross represents the censoring event at the end of the follow-up period of the specific CHMI (day 28 after CHMI). Two volunteers receiving placebo withdrew consent before CHMI; these individuals were not included in the survival analysis. Time to treatment: treatment was initiated at time of parasitemia. Parasitemia as the CHMI endpoint was defined as at least one qPCR result above 100 parasites/ml among three positive results at least 12 h apart or as a positive thick blood smear.

After CHMI with heterologous PfSPZ (7G8), 10/13 vaccine and 0/5 placebo volunteers were sterilely protected. VE compared to placebo was 77% (95% CI: 13–95%, *p* = 0.004, Boschloo’s test, two tailed). Participants developed parasitemia detected by quantitative real-time reverse transcriptase PCR (RT-qPCR) with a median prepatent period of 7 days (interquartile range (IQR) 0) after CHMI; median peak parasite density was 2925 parasites/ml (IQR 2168–6218) in the three unprotected vaccine participants and 11,555 parasites/ml (IQR 7937–12,563) in the placebo group. Median time to treatment threshold was 9 days (IQR 9.0–10.0) in the placebo group and 10 days (IQR 9.5–12.0) in the unprotected vaccinated participants, which was not significantly different (Fig. 4). Distribution of age between the two groups was similar (median age of 29 years in unprotected vs 26 years in protected vaccinees, Mann–Whitney test, *p* = 0.84). Of note, all unprotected participants were males.

The 10 protected individuals in general had higher levels of parasitemia after the first two doses of PfSPZ-CVac (Fig. 3), but after the third dose, one unprotected subject had a high parasitemia above 1000 parasites/ml. Only 6 of the 10 protected subjects and 1 of the 3 unprotected subjects had a parasitemia level above the detection limit after the third immunizing dose; the other six of these subjects did not develop any detectable parasitemia (detection limit: 6 parasites/ml) after injection with a dose of PfSPZ Challenge (PfNF54) that was 34.5 times higher than the 100% infectious dose of PfSPZ Challenge (PfNF54) used for CHMI.

### Safety and reactogenicity

**Fig. 5:**
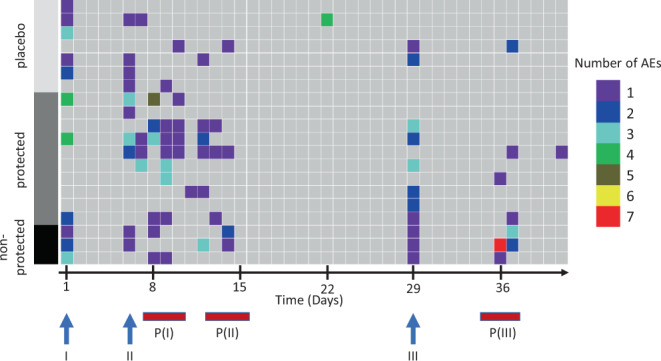
Adverse events (AEs) over time during immunization period. AEs during the immunization period were recorded for each individual. The total number of mild and moderate AEs per individual (rows) and per day (columns) are plotted as a heatmap. The study population was divided into the subgroups of treatment allocation and CHMI outcome. Numbers of AEs are represented as given by the color scheme in the legend. Arrows highlight the time points of injection of the investigational product. Bars in burgundy indicate the period of transient parasitemia in vaccinees. AEs on Day 1 occurred after administration of investigational product. There were no AEs on days 2–5 after dose I or on days 30–35 after dose III.

**Fig. 6:**
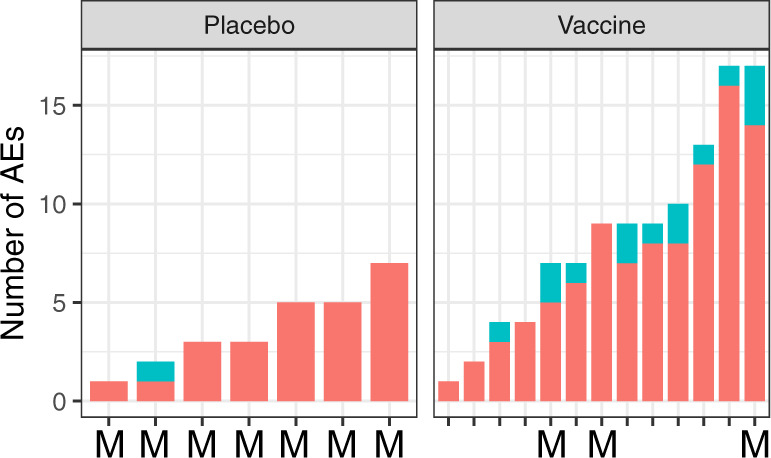
The number of adverse events (AEs) during the immunization period in the placebo and vaccine groups. Each bar represents one volunteer sorted on the number of AEs from the time of first injection with normal saline or PfSPZ Challenge until the end of the vaccination period. Mild (grade 1) AEs are depicted in red, moderate (grade 2) in turquoise. Non-protected volunteers are marked with an “M” on the *x*-axis.

There were no related grade 3 adverse events (AEs) or serious adverse events (SAEs) during the immunization phase, but there was one unrelated grade 3 AE, a case of diastolic hypertension. In all, 222 grade 1 or 2 AEs occurred during the immunization period. One hundred and thirty-five (109 in vaccinees and 26 in controls) were considered as possibly, probably, or definitely related to the investigational product (Table 1). Headache and/or dizziness occurred in 92.3% of vaccinees and 71.4% of controls, and fatigue in 69.2% of vaccinees and 42.9% of the controls, suggesting that CQ may have played a significant role in causing AEs. Solicited grade 1 and 2 AEs within the first 5 days following the first and third immunization are summarized in Table 2. Nervous system disorders occurred in 46% of vaccinees and 43% of placebo controls after the first vaccination/CQ treatment, and 46% and 14% after third vaccination/CQ treatment for vaccinees and placebo controls, respectively (Supplementary Table 2). The second vaccine administration is not shown as this time overlaps with the time period of transient parasitemia. At most, three AEs occurred per individual per day, and they primarily occurred shortly after CQ treatment or during transient parasitemia on days 7 or 8 (Fig. 5). The range of AEs occurring in subjects in the vaccinated and placebo groups was 1–17 AEs and 1–7 AEs, respectively (Fig. 6).

**Table 2:** Related grade 1 and 2 AEs using MedDRA terminology during the immunization phase Days −2 to +42).

System organ class	Preferred term	Vaccine (*N* = 13)	Placebo (*N* = 7)
Blood and lymphatic system disorders	Lymphopenia	[1] 1 (7.7%)	–
	#Total	[1] 1 (7.7%)	–
Cardiac disorders	Tachycardia	[2] 2 (15.4%)	–
	#Total	[2] 2 (15.4%)	–
Gastrointestinal and abdominal pains (excl. oral and throat)	Abdominal pain	[1] 1 (7.7%)	[2] 1 (14.3%)
	#Total	[1] 1 (7.7%)	[2] 1 (14.3%)
Gastrointestinal disorders	Diarrhea	[2] 2 (15.4%)	[1] 1 (14.3%)
	Nausea	[7] 6 (46.2%)	[3] 2 (28.6%)
	#Total	[9] 7 (53.8%)	[4] 3 (42.9%)
General disorders and administration site conditions	Chills	[3] 3 (23.1%)	[1] 1 (14.3%)
	Fatigue	[16] 9 (69.2%)	[3] 3 (42.9%)
	Hyperhidrosis	[4] 4 (30·8%)	–
	Malaise	[4] 3 (23.1%)	–
	Pyrexia	[8] 5 (38.5%)	[1] 1 (14.3%)
	#Total	[35] 10 (76.9%)	[5] 3 (42.9%)
Infections and infestations	Oral herpes	–	[1] 1 (14.3%)
	#Total	–	[1] 1 (14.3%)
Musculoskeletal and connective tissue disorders	Back pain	[1] 1 (7.7%)	–
	Myalgia	[5] 4 (30.8%)	[1] 1 (14.3%)
	#Total	[6] 4 (30.8%)	[1] 1 (14.3%)
Nervous system disorders	Dizziness	[15] 7 (53.8%)	[4] 2 (28.6%)
	Headache	[35] 12 (92.3%)	[8] 4 (57.1%)
	Paresthesia	[1] 1 (7.7%)	–
	Vision blurred	[3] 2 (15.4%)	–
	#Total	[54] 12 (92.3%)	[12] 5 (71.4%)
Psychiatric disorders	Depersonalization/derealization disorder	–	[1] 1 (14.3%)
	#Total	–	[1] 1 (14.3%)
Respiratory, thoracic, and mediastinal disorders	Tachypnoea	[1] 1 (7.7%)	–
	#Total	[1] 1 (7.7%)	–

In square brackets: number of events; number of patients with events; in brackets: percentage of patients with event.

No differences were statistically significant (*p* > 0.10, Fisher’s exact test, two-sided for all).

There was no significant difference between the 13 vaccinees and 7 controls in regard to any single AE at the time of peak parasitemia (Fig. 3a) after each of the three immunizations, which was on days 7–9 after each dose (Fig. 3b). However, after each dose, there was a higher frequency of headache in vaccinees (62, 15, and 31%) as compared to controls (14, 14, and 14%); the difference in the frequency after the first dose was 62% vs 14% (*p* = 0.07, Fisher’s exact test, two sided). No other AE occurred during this time window after any dose in more than 32% of vaccinees or controls. The highest incidence of pyrexia occurred after the first dose (23% vs 0%, *p* = 0.25); 15% vs 0% after second dose, and 8% vs 0% after the third dose. The participant with pyrexia after the third dose had the highest parasitemia on day 8 after vaccination (1262 parasites/ml). All of these AEs were grade 1 or 2 (Supplementary Table 2).

Six of the eight participants, who developed parasitemia during CHMI, experienced at least one AE related to malaria, including one grade 3 AE (neutropenia). Volunteers were treated with atovaquone/proguanil according to the national guidelines and all recovered uneventfully.

One unrelated SAE occurred in the late follow-up period. On day 63 after CHMI, one vaccinee underwent an elective surgery for an anal fissure.

### Immunogenicity against PfCSP

**Fig. 7:**
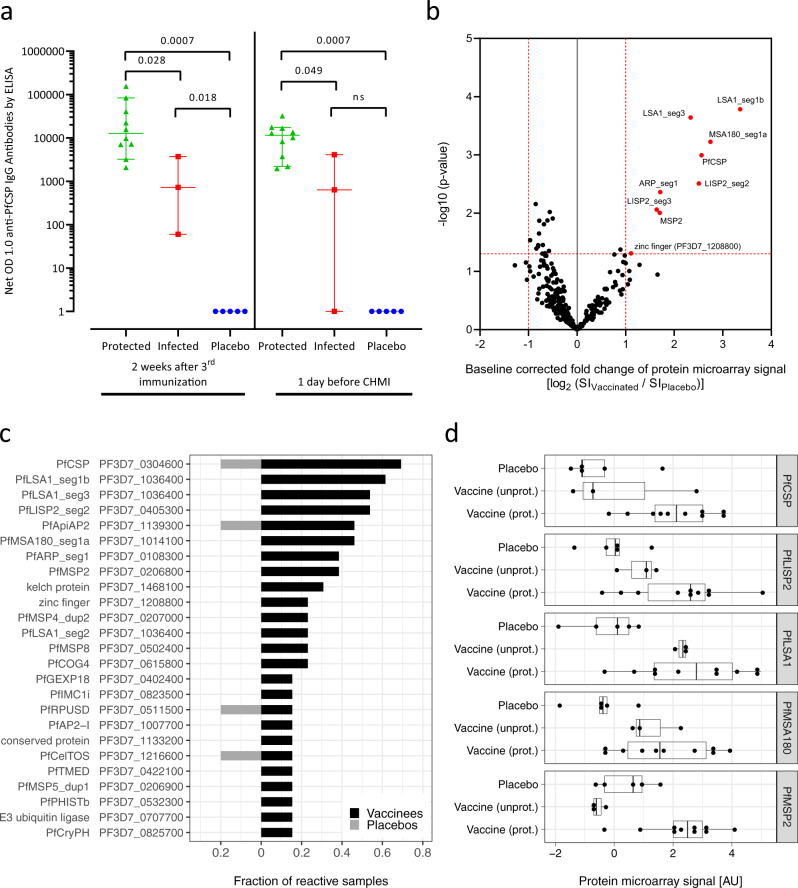
IgG antibody reactivity against *Plasmodium falciparum* antigens. **a** IgG antibody levels specific for PfCSP were measured by ELISA using sera from volunteers before the first immunization (D-1), 15 days after the last immunization (D 44), as well as 1 day before CHMI (C-1). PfCSP-specific antibody levels of baseline-corrected post-immunization time points (net OD 1.0) are shown. Green triangles: vaccinated group (protected); red squares: vaccinated group (unprotected); blue circles: placebo controls. Reported data were derived from a single assessment with three technical replicates. *N* = 18 biologically independent study participants. Lines shown are median with 95% CI. *P* values were estimated by two-sided Wilcoxon–Mann–Whitney test. **b**–**d** Sera from all volunteers collected before immunization (baseline, D-1) and one day before CHMI (C-1) were assessed on protein microarrays containing 262 *P. falciparum* proteins representing 228 unique antigens. Analysis was performed on C-1 data after subtraction of the individual baseline reactivity. **b** To estimate PfSPZ-CVac immunogenicity, antigen reactivity in vaccinated donors (to the right) was compared to the placebo controls (to the left). Differentially recognized antigens (*p* value <0.05 and fold change >2) are depicted in red. *P* values were estimated using the two-sided Welch-corrected Student’s *t*-test. **c** The fraction of seropositive vaccinated and placebo samples (seropositivity defined as at least fourfold overall baseline reactivity) of the 25 most immunogenic antigens were assessed. **d** Individual antibody reactivities to the five most immunogenic proteins representing sporozoite, liver, and early blood stage are presented stratified by the group allocation (placebo, unprotected, and protected vaccinees). The boxplot gives median signal intensities, interquartile ranges (IQR), and whiskers of the length of 1.5 × IQR. *N* = 18 biologically independent study participants.

IgG antibody responses (net OD 1.0) to PfCSP 2 weeks after the third dose and just prior to CHMI were 17.6 and 18.1 times higher in uninfected (protected) (*N* = 10) vs infected (*N* = 3) vaccinees (*p* = 0.028 and 0.049) (Fig. 7a). Quantification by a second assay of anti-PfCSP-specific IgG one day before CHMI showed that median PfCSP-specific IgG was 10-fold higher in protected (127 µg/ml, 95% confidence interval (CI): 36–231 µg/ml) than in unprotected vaccinees (18 µg/ml, 95% CI: 6–33 µg/ml). Median levels of IgM antibodies to PfCSP 2 weeks after the third dose and 1 day before challenge were 6.7 and 2.5 higher in uninfected (protected) vs infected vaccinees, but differences did not reach the level of statistical significance (*p* = 0.112 and 0.119) (Supplementary Fig. 1). IgG antibody responses to PfCSP were in general highest 2 weeks after the third dose and remained elevated through 28 days after CHMI (Supplementary Fig. 2A). IgM response also peaked after the third dose, but decreased by the time of CHMI in most volunteers (Supplementary Fig. 2B); thus, antibody levels were not boosted during CHMI.

### Immunogenicity estimated by protein microarrays

The overall antibody response against the parasite in vaccinees compared to the placebo controls was estimated by protein microarray^17–20^. Before CHMI, significantly elevated antibody levels were detected for multiple proteins (Fig. 7b). For the proteins PfLSA1 (Pf liver stage antigen 1), PfMSA180, PfCSP, PfLISP2 (liver-specific protein 2), and Pf merozoite surface protein 2 (PfMSP2), more than half of the vaccinated cohort reached antibody levels at least four times higher than did placebo controls (Fig. 7c), corresponding to a high effect size (Hedge’s *g* > 0.9).

Protection from development of blood stage parasitemia following CHMI was associated with significant increases in five Pf-specific antibodies, PfMSP2, sporozoite invasion-associated protein 2 (PfSIAP2), chaperone protein DnaJ (PfDnaJ protein), gametocytogenesis-implicated protein (PfGIG), and a member of the erythrocyte membrane protein 1, PfEMP1 (*p* < 0.05, Welch’s corrected Student’s *t*-test). As expected from the enzyme-linked immunosorbent assay (ELISA) data, IgG levels against PfCSP were higher in the protected than the unprotected vaccinees (Hedge’s *g* > 0.8). Interestingly, antibodies against PfMSP2 were 7.5-fold increased in protected vs unprotected vaccinees (Supplementary Fig. 3).

In order to verify the general vaccination effect in the unprotected study population, the antibody levels against representative markers of the different stages, which the parasite pass through under CQ treatment, were more closely investigated (Fig. 7d). Despite the lower PfCSP-specific reactivity of the unprotected subjects in the vaccine group compared to the protected subjects in the vaccine group, antibody levels against early-to-mid liver stage antigens (PfLSA1, PfLISP2) and one late liver stage antigen (PfMSA180) were similarly elevated in the protected and the unprotected volunteers of the vaccine group. IgG levels against PfMSP2 were as low in the vaccinated but unprotected subjects as in the placebo group.

Concerning IgM antibody levels, PfMSP2, PfLSA1, and PfMSA180 IgM antibodies were elevated in the vaccinees compared to the placebo controls and only an asparagine-rich protein with unknown function (conserved protein, PF3D7_0817300) was associated with protection (Supplementary Fig. 4).

## Discussion

Immunization with chemoattenuated PfSPZ in a condensed regimen with three doses of 1.1 × 10^5^ PfSPZ administered on Days 1, 6, and 29 with 10 mg/kg CQ base protected 77% of malaria-naive volunteers against heterologous CHMI 12 weeks after the immunization. This study established three key principles that are critical for moving forward with PfSPZ-CVac: (1) VE was demonstrated against a heterologous strain of Pf parasite, (2) VE was achieved after a 4-week immunization period, and (3) VE and safety were achieved while administering CQ only on the days of administration of PfSPZ.

For implementation of this vaccine for use in endemic areas, it was crucial to demonstrate VE against heterologous CHMI. It has been reported that immunization by mosquito bite gave only minimal protection against heterologous CHMI^15,16^. Here, we used Pf7G8, which originates from Brazil. Compared to PfNF54, Pf7G8 possesses tens of thousands of genetic variations, including regulatory and immunologically important regions^21^. More importantly, Pf7G8 varies more from PfNF54 at the genome, proteome, and CD8 T cell immunome level than do >700 Pf strains from East, West, and Central Africa^21^. Furthermore, it has been shown that when the same immunization regimen of PfSPZ Vaccine is administered, it is more difficult to protect against heterologous CHMI with Pf7G8 than against field acquired malaria in Mali^9,10^. We think that we achieved such good VE against a heterologous parasite, because we increased the immunizing dose more than twofold from our studies that gave 100% VE against homologous CHMI^12^, and because immunization on Days 1 and 6 achieves multi-dose priming, which enhances VE. In the mosquito bite CPS studies that did not show heterologous VE^15,16^, the subjects were immunized with only a maximum of bites from 15 infected mosquitoes three times (total of 45). It requires the bites of five Pf-infected mosquitoes or 3.2 × 10^3^ PfSPZ of PfSPZ Challenge to achieve 100% infection of subjects in CHMI^5,22^. The two systems to infect humans with Pf sporozoites are difficult to compare directly, as the number of Pf sporozoites injected by a mosquito is highly variable, but nevertheless we estimate that the dose of 15 mosquito bites is the equivalent of 9.6 × 10^3^ PfSPZ, which is approximately a 10-fold lower dose than the 1.1 × 10^5^ PfSPZ we used in this study.

A number of CPS and PfSPZ-CVac regimens with different routes of administration (mosquito bite, intradermal injection, DVI), intervals between doses and numbers of doses of PfSPZ have been assessed in the last decade with intention to evoke sterile VE. With PfSPZ administered by mosquito bite (CPS) or by DVI (PfSPZ-CVac), VE was 75–100%^12,13,23^ against homologous CHMI using longer vaccination schedules (three doses at 4-week intervals). Our results from a former PfSPZ-CVac trial suggested that shorter three-dose regimens—desirable for general use—show reduced VE. Therefore, in retesting a 5-day interval between the first and second doses in this trial, we increased the dose of PfSPZ and delayed the third dose from Day 11 to Day 29 to improve VE. The increased dose of PfSPZ might raise concerns about a higher parasite density at the time of parasite egress from the liver with more pronounced symptoms and signs of malaria, eventually carrying a higher risk of a possible breakthrough infection during the immunization phase. However, our data showed that parasitemia was at submicroscopic levels and was efficaciously cleared by the simplified dosing of CQ during immunization. However, as in the previous study, parasite egress into the blood 7–9 days after the first immunization was associated with a non-significant, self-limiting increase in grade 1 or 2 headaches (62% of vaccinees), pyrexia (32%), and fatigue (31%); the incidence rate was lower after second and third doses (Supplementary Table 1).

In previous studies we administered a loading dose of 10 mg/kg CQ base 2 days before the first immunization and then 5 mg/kg weekly thereafter through 5 days after the last immunization for a total of 10 doses (Fig. 1). This is a rigorous schedule to follow and we have been concerned that if after administration of the first dose of PfSPZ, vaccinees were lost to follow-up and did not take their subsequent CQ doses, they could develop malaria. In this study the CQ was administered prior to each dose, ensuring the CQ was swallowed and retained before PfSPZ were administered (Fig. 1). For all three doses 10 mg/kg CQ base was administered. This approach was adequate to kill all the blood stage parasites. However, 71.4% of the controls had headache and/or dizziness, which we speculate to be attributed to CQ.

It would be useful if it was possible to predict protective efficacy. One possibility might be the prediction of protection based on the peak parasitemias seen after each immunizing dose. After the first and second doses, vaccinees who were protected had greater median levels of peak parasitemia (1187 vs 501 after first dose and 651 vs 150 after second dose). We will continue to investigate whether RT-qPCR results can be used to predict protective efficacy.

A number of studies of PfSPZ vaccines have shown that protected vaccinees had significantly higher antibodies to PfSPZ as measured by PfCSP ELISA, PfSPZ immunofluorescence assay, and/or inhibition of sporozoite invasion of hepatocytes^10,11^. In our previous study of PfSPZ-CVac, the nine highest dose subjects had the highest levels of antibodies to PfCSP, but all were protected, so we could not try to correlate protection with antibody level. In the intermediate dose group 6/9 were protected, and the difference in PfCSP antibody levels between protected and infected subjects was not significant^12^. In this study the 10 protected vaccinees had significantly higher levels of antibodies to PfCSP 2 weeks after the last immunization, and prior to CHMI, than did the three infected vaccinees. This indicates that anti-PfCSP antibodies either play a role in protection and/or are a biomarker for other protective immune responses. In animal studies protection by sporozoite vaccines is dependent on cellular immune responses, especially CD8 T cells^24,25^. Thus, the anti-PfCSP antibody levels can serve as a correlate of protection, even though we cannot elucidate the mechanistic function in this work. Nevertheless, PfCSP-specific antibodies are highly functional in inhibiting sporozoite invasion into the liver and were recently shown to neutralize sporozoites in the liver^26,27^.

In our previous work, using a whole proteome microarray, 22 antigens were identified by qualitative analysis as specifically immunogenic (recognized by 5/9 subjects) after immunization with 5.12 × 10^4^ PfSPZ of PfSPZ-CVac^12^. Of these 22 antigens, 7 were on the microarray used in this study, including PfCSP, PfMSP4, PfLSA1, PfGLURP, PfLISP2, and two unknown function antigens. Our microarray data confirm the immunogenicity of PfCSP, PfLSA1, and PfLISP2 but we did not identify elevated antibodies against PfMSP4, PfGLURP, or the two unknown function antigens. In addition, we confirmed the immunogenicity of PfMSP2, which has been described before^12,18,20^. We also identified several novel or less considered markers of vaccination. These include PfMSA180, a protein with unknown function (PF3D7_01083Na00) and the sporozoite surface protein, PfSIAP2. In the microarray studies, only PfMSP2 was consistently recognized by sera from protected vs infected subjects (Fig. 7d). The expression of the antigen PfMSP2 has been confirmed by immunofluorescence in day 5 Pf liver stage^28^. This result shows that vaccine-induced immunity to the late liver stage is of importance for the protection. The majority of identified immune markers, especially LSA1 (ref. ^29^) and MSP2 (ref. ^30^), but also SIAP2 (ref. ^31^) and most recently MSA180 (ref. ^31^), a merozoite surface protein with assumed function during erythrocyte invasion, have been suggested as promising targets for peptide-based vaccines^32^.

Despite the promising, high level and cross-strain protection, our study has limitations. The number of participants in this early phase of clinical development was low. Thus, possible individual differences could lead to overrepresentation of chance effects. Furthermore, the longevity of protection beyond 12 weeks induced by this condensed PfSPZ-CVac regimen needs to be followed-up. The long-term protection against heterologous CHMI is part of the study protocol and will be further investigated. However, in the CPS model, durable immunity for 28 months against homologous CHMI was achieved^14^.

A 4-week immunization regimen with PfSPZ-CVac, in which the PfSPZ and CQ were administered on the three same days (Days 1, 6, and 29), was a practical and effective immunization strategy that reached more than 75% cross-strain protection in CHMI 12 weeks after immunization. This work is an important step in the clinical development of PfSPZ-CVac for protection against malaria and a milestone towards regulatory approval. Further optimization of the study regimen will include the replacement of oral CQ as the chemoprophylactic drug. To increase tolerability of vaccination and to ensure the administration of the drug, we aim to replace oral CQ by intramuscular pyronaridine.

We believe that PfSPZ-CVac will be an excellent vaccine for travelers to Africa, as usually more than 90% of travelers are voyaging for less than 12 weeks, and a vaccination regimen as proposed here can be well-included into the travel preparation. However, our major goal is to conduct studies to assess PfSPZ-CVac in 2–12-year-old African children, the demographic group that suffers the greatest morbidity and mortality from malaria, and is responsible for most of the transmission of malaria^33^. We believe that PfSPZ-CVac will be able to meet the strategic goals of the WHO Preferred Product Characteristics for Malaria Vaccines^34^.

## Methods

### Design and participants

This single-center, double-blinded, randomized trial was conducted at the Institute of Tropical Medicine, University of Tübingen, Tübingen, Germany, from April 2019. Screening was started on 2 May 2019 and first vaccination was done on 15 May 2019. The last volunteer was vaccinated on 23 May 2019. Planned trial close out was November 2020, but this had to be postponed due to the COVID-19 pandemic. Therefore, it was decided to report the data of this interim analysis. The first CHMI was performed in September 2019 with a follow-up until November 2019. This interim analysis on the vaccine safety, efficacy, and immunogenicity was predetermined in advance in the study protocol, and the safety monitoring committee reviewed the reports on safety and tolerability data.

The trial was approved by the Paul Ehrlich Institute and the Clinical Ethics Committee at the University Hospital of Tübingen (UKT). The study design and conduct complied with all relevant regulations regarding trials with human study participants and was conducted in accordance with the criteria set by the Declaration of Helsinki. The study was performed in accordance with Good Clinical Practice/International Conference on Harmonisation guidelines. The trial is registered in the European Union Clinical Trials Register (EudraCT-Nr: 2018-004523-36).

Healthy, malaria-naive volunteers aged 18–45 were recruited in Tübingen and surrounding areas. All participants provided written informed consent at the screening visit. Female volunteers were required to practice continuous effective birth control during the study period. For safety reasons, volunteers were required to be reachable 24/7 by mobile phone. Prior to enrollment, participants had to pass a questionnaire assessing the understanding of risks and obligations of the trial. Main exclusion criteria were history of malaria or previous participation in a malaria vaccine trial and any relevant medical history. The full list of eligibility criteria is listed in the protocol which is available in the supplementary material.

### Procedures

Participants were randomly allocated to immunization with either 1.1 × 10^5^ PfSPZ of PfSPZ Challenge (PfNF54) or normal saline as placebo on the day of first vaccination. All vaccinations were administered by DVI in 0.5 ml on Days 1, 6, and 29.

All volunteers received 10 mg/kg CQ base orally (up to a maximum dose of 620 mg CQ base) within 2 h prior to each immunization (Resochin, Bayer Schering Pharma). PfSPZ Challenge (PfNF54) for immunization consisted of aseptic, purified, cryopreserved, infectious PfSPZ, strain PfNF54, produced by Sanaria Inc. (Rockville, USA). PfSPZ were stored and transported in liquid nitrogen vapor phase and thawed and diluted at the Institute of Tropical Medicine, Tübingen within 20 min before DVI.

Twelve weeks after the last dose of immunization with PfSPZ or placebo, all volunteers underwent CHMI by DVI of 3.2 × 10^3^ PfSPZ Challenge^22^, using the heterologous Pf strain 7G8 to assess VE. Pf7G8 is derived from a Brazilian isolate^35^, is antigenically highly divergent from PfNF54 the West African vaccine strain^21^, and in prior challenge studies has been harder to protect against than for CHMI using homologous PfNF54 (ref. ^10^), thereby increasing the stringency of the CHMI. Thick blood smear (TBS) and RT-qPCR were performed daily from Day 6 to 21 and on all later follow-up visits (Day 28 and 56) after CHMI as described earlier^12^. Subjects were treated regardless of symptoms in case of TBS positivity or three positive PCR results, at least 12 h apart and at least one of them above 100 parasites/ml. First-line treatment was 1000 mg atovaquone and 400 mg proguanil once daily for three consecutive days.

### Randomization and masking

Randomization was performed on the day of first immunization prior to injection by a third party outside the study team and sponsor. The randomization list was generated using a random number generator (Mersenne-Twister implemented in R; www.r-project.org) and given to a dedicated member of the formulation team who did not have a further role in the trial. The allocation ratio for PfSPZ Challenge to placebo was 2:1. All syringes used were identical and labeled with the volunteer identification code. PfSPZ Challenge (PfNF54) and placebo were both clear fluids and not distinguishable by appearance or consistency. Clinical team, funder, and volunteers remained blinded until database lock. First unblinding was done following an interim database lock after Day 56 of CHMI to allow assessment of VE by an independent statistician.

### Outcomes

The aim of the trial was to assess safety, tolerability, and efficacy of a condensed immunization regimen with three doses of PfSPZ Challenge and CQ. The primary VE endpoint was the proportion of protected volunteers. Protection was defined as the absence of parasitemia in the peripheral blood for 28 days after CHMI. According to the study protocol, parasitemia was defined as at least one RT-qPCR result above 100 parasites/ml among three positive results, at least 12 h apart or as a positive TBS.

The primary safety endpoint was the occurrence of related grade 3 and 4 AEs events following the first CQ administration until the end of the trial. Related AEs were recorded and reported using the terminology defined in the Medical Dictionary for Regulatory Activities (MedDRA).

Further exploratory endpoints were time to parasitemia (defined as the time to the first positive RT-qPCR result among three positive results at least 12 h apart, with at least one of them being above 100 parasites/ml, or the time to a positive TBS) and the characterization of immune responses including the identification of parasitological and immunological correlates of protection against CHMI.

### Immunological assays

IgG and IgM antibodies to the PfCSP were assessed by titration ELISA, and in addition by quantitative ELISA, as described^12,36^. Antibody reactivity against Pf antigens was assessed by protein microarray (array design in supplementary Table 1). The array comprises 262 Pf protein fragments representing 228 unique proteins down-selected from previous larger microarray screens^19,20^ and performed as described^37^. A detailed description of the immunological assays is available in the supplement.

### Statistical analysis and sample size

To detect infection rates of 25% or less in the vaccination and 85% in the placebo groups (allocated in a 2:1 ratio) with a power of 80% and a two-tailed alpha of 5%, 14 PfSPZ-CVac immunized, and 7 placebo-treated volunteers were required. Sample size was calculated in R using the nBinomial function provided in the gsDesign package v3.2.

Safety and tolerability data are presented as descriptive analyses. VE was calculated as 1−relative risk of reaching the parasitemia endpoint for vaccinated participants compared to unvaccinated participants. Proportions between immunized and placebo-treated volunteers were compared with an unconditional exact test (Boschloo’s test) using R (exact2x2 package v1.6.5). The level of significance was set at a two-tailed type 1 error alpha <5%.

Participants, investigators, and diagnostic team remain blinded until completion of all CHMI procedures and data collection.

All statistical analyses were performed using R, version 4.0.4, and GraphPad Prism 9.0.2.

### Reporting summary

Further information on research design is available in the Nature Research Reporting Summary linked to this article.

## Supplementary information

Supplementary Information

Reporting Summary

## Data Availability

Individual participant data that underlie the results reported in this publication are available from the clinical trial sponsor on the basis of a data sharing agreement on reasonable request. The data are not publicly available due to them containing information that could compromise research participant privacy and consent. The study protocol is available in the supplementary material. Correspondence should be submitted to R.F. (rolf.fendel@uni-tuebingen.de).
