# Supplementary material for: Heterologous protection against malaria by a simple chemoattenuated PfSPZ vaccine regimen in a randomized trial
Source: Nat Commun. 2021 May 4;12:2518. doi: 10.1038/s41467-021-22740-w (PMC8097064; doi:10.1038/s41467-021-22740-w)
Supplement: Supplementary file 1 — Supplementary Information [file 41467_2021_22740_MOESM1_ESM.pdf]

Supplements

Supplementary methods

Enzyme-linked immunosorbent assays (ELISA)

PfCSP-specific IgG and IgM antibody reponses were assessed using the antigen and methodology described. [1] The serum dilution at which the optical density (OD) was 1·0 (OD 1·0) was determined. Samples were considered positive if the difference between the post-immunization OD 1·0 and the pre-immunization OD 1·0 (net OD 1·0) was ≥ 50 and the ratio of post-immunization OD 1·0 to pre-immunization OD 1·0 (ratio) was ≥ 3.

In addition a second ELISA was done to quantify anti-PfCSP IgG and IgM antibodies as described with minor modifciations. [1] For this assay, PfCSP (sequence from NF54, Genbank: XP_001351122, amino acid 199-377) was recombinantly expressed in *Escherichia coli (E. coli)* strain BL21(DE3) using a stress expression system as described before. [2] The antigen construct contained both a pelB-leader sequence for periplasmic expression and a C-terminal His_6-tag, allowing the subsequent protein purification by immobilized-metal affinity chromatography (IMAC).

Pooled sera from a European PfSPZ-immunized study cohort (ClinicalTrials ID: NCT02704533) and a second pool from non-immunized European donors were used as positive or negative controls respectively. Both controls were included on every plate and used for standardization of plate-to-plate variances.

Microplates were coated with 20 ng PfCSP per well at a concentration of 200 ng/ml in 0·1 M sodium bicarbonate (pH 9·6) at 4°C overnight. Between each of the following steps, plates were washed using a 1X PBS/0·1% Tween 20 washing buffer. Wells were blocked using 1X PBS supplemented with 5% BSA, 0·1% Tween 20 and 0·5 mM EDTA for two hours at room temperature (RT). Subsequently, controls and study samples were applied at eight two-fold dilutions in blocking buffer, ranging from 1:400 to 1:51,200. Bound IgG was detected with horseradish peroxidase conjugated goat anti-human IgG (Jackson ImmunoResearch, # 109-035-098) at a dilution of 1:20,000

in blocking buffer. The signal was developed using TMB substrate and stopped using 50 µl of 1 M hydrochloric acid when the TMB reaction reached an OD of 1·5 to 2 at 350 nm. The absorbance was read at 450 nm (specific signal) and 620 nm (background control). For the estimation of PfCSP-specific IgM antibodies, the same procedure was applied, with the exception that the blocking buffer was 1X RotiBlock (Roth) and the secondary antibody was HRP-conjugated goat anti-human IgM (ImmunoReagents, # GtxHu-006-E2HRPX) at a dilution of 1:5,000. Microplates were measured using the BMG CLARIOstar with Clariostar Software Version 5.40 R2 and MARS Data Analysis Software Version 3.31.

For each sample, the area under the curve (AUC) was calculated and normalized to the AUC of the positive and negative control measured on the same plate. Results are given as relative AUC [$(AUC_{sample}-AUC_{negative\ control}) / (AUC_{positive\ control}-AUC_{negative\ control})$]. For graphical representation of the results, individual baseline values were subtracted.

To calculate the absolute IgG and IgM concentration, a four-parameter logistic curve was fitted to the results of IgG and IgM positive control standards and the respective concentrations in the sera using R statistical software package version 4.0.4. Simple logistic regression was used to predict the probability of protection based on PfCSP-specific antibody levels. The respective p-value was estimated using the likelihood ratio test implemented in GraphPad Prism version 9.0.2.

Microarray assay

Microarrays were produced at the University of California Irvine, Irvine, California, USA. [3] In total, 262 *P. falciparum* proteins representing 228 unique antigens were expressed using an *E. coli* lysate *in vitro* expression system and spotted on a 16-pad ONCYTE AVID slide, representing 228 important *P. falciparum* antigens known to frequently provide a positive signal when tested with sera from those with sterile and naturally acquired immunity against the parasite. For the detection of binding antibodies, secondary IgG antibody (goat anti-human IgG QDot™800, Grace Bio-Labs #110635),

secondary IgM antibody (biotin-SP-conjugated goat anti-human IgM, Jackson ImmunoResearch #109-065-043) and Qdot™585 Streptavidin Conjugate (Invitrogen #Q10111MP) were used.

Study serum samples as well as the European control serum were diluted 1:50 in 0·05X Super G Blocking Buffer (Grace Bio-Labs, Inc.) containing 10% *E. coli* lysate (GenScript, Piscataway, NJ) and incubated for 30 minutes on a shaker at room temperature (RT). Meanwhile, microarray slides were rehydrated using 0·05X Super G Blocking buffer at RT. Rehydration buffer was subsequently removed and samples added onto the slides. Arrays were incubated overnight at 4°C on a shaker (180 rpm). Serum samples were removed the following day and microarrays were washed using 1X TBST buffer (Grace Bio-Labs, Inc.). Secondary antibodies were then applied at a dilution of 1:200 and incubated for two hours at RT on the shaker, followed by another washing step and a one-hour incubation in a 1:250 dilution of Qdot™585 Streptavidin Conjugate. After a final washing step, slides were dried by centrifugation at 500 g for 10 minutes. Slide images were taken using the ArrayCAM® Imaging System (Grace Bio-Labs) and the ArrayCAM 400-S Microarray Imager Software version 2.2.

Microarray data analysis

Microarray data were analyzed in R statistical software package version 4.0.4. All images were manually checked for any noise signal. Each antigen spot signal was corrected for local background reactivity by applying a normal-exponential convolution model [4] using the RMA-75 algorithm for parameter estimation (available in the LIMMA package v3.28.14). [5] Data was log2-transformed and further normalized by subtraction of the median signal intensity of mock expression spots on the particular array to correct for background activity of antibodies binding to *E. coli* lysate. After log2 transformation data were normally distributed. Differential antibody levels (protein array signal) in the different allocated study outcomes (placebo, non-protected and protected vaccinees) were determined by Welch-corrected Student’s t-test. Antigens with $p < 0·05$ and a fold change > 2 of mean signal intensities were defined as differentially recognized between the tested sample groups.

Box plots and volcano plots were generated using the ggplot2 v3.3.3 and PAA v3.12 packages, respectively.

Supplementary Table 1. Grade 1 and 2 AEs using the MedDRA terminology during the first five days after CQ treatment and vaccine administration (Day of Vaccination to five days post vaccination) for the first and the third vaccine administration. The second vaccine administration is not shown as this time overlaps with the time period of transient parasitemia. No differences were statistically significant (for all, $p > 0.10$, Fisher's exact test, 2-tailed)

		**Immunization 1**		**Immunization 3**	
**System organ class**	**Preferred Term**	**Placebo (N=7)**	**Vaccine (N=13)**	**Placebo (N=7)**	**Vaccine (N=13)**
Gastrointestinal and abdominal pains (excl. oral and throat)	Abdominal pain				[1] 1 (8%)
	#Total				[1] 1 (8%)
Gastrointestinal disorders	Diarrhoea	[1] 1 (14%)			[1] 1 (8%)
	Nausea		[3] 3 (23%)		
	#Total	[1] 1 (14%)	[3] 3 (23%)		[1] 1 (8%)
General disorders and administration site conditions	Fatigue	[2] 2 (29%)	[3] 2 (15%)		[1] 1 (8%)
	Malaise		[1] 1 (8%)		[2] 2 (15%)
	#Total	[2] 2 (29%)	[4] 2 (15%)		[3] 3 (23%)
Infections and infestations	Oral herpes			[1] 1 (14%)	
	#Total			[1] 1 (14%)	
Nervous system disorders	Dizziness	[2] 1 (14%)	[4] 4 (31%)	[1] 1 (14%)	[5] 5 (38%)
	Headache	[2] 2 (29%)	[4] 4 (31%)	[1] 1 (14%)	[5] 4 (31%)
	Paraesthesia				[1] 1 (8%)
	Vision blurred		[1] 1 (8%)		
	#Total	[4] 3 (43%)	[9] 6 (46%)	[2] 1 (14%)	[11] 6 (46%)
Psychiatric disorders	Depersonalisation/derealisation disorder	[1] 1 (14%)			
	#Total	[1] 1 (14%)			

In square brackets: number of events; number of patients with events; in brackets: percentage of patients with event.

Supplementary Table 2: Related grade 1 and 2 AEs using the MedDRA terminology during transient parasitemia after each immunization (7 to 9 days after each immunization). The statistical test used Fisher's exact test, two-sided for all.

		Immunization 1		Immunization 2		Immunization 3		Any Immunization		
**System organ class**	**Preferred Term**	**Placebo (N=7)**	**Vaccine (N=13)**	**Placebo (N=7)**	**Vaccine (N=13)**	**Placebo (N=7)**	**Vaccine (N=13)**	**Placebo (N=7)**	**Vaccine (N=13)**	**Fisher's exact test**
Blood and lymphatic system disorders	Lymphopenia	-	-	-	-	-	[1] 1 (8%)	-	[1] 1 (8%)	>0·9999
	#Total	-	-	-	-	-	[1] 1 (8%)	-	[1] 1 (8%)	>0·9999
Cardiac disorders	Tachycardia	-	[1] 1 (8%)	-	-	-	[1] 1 (8%)	-	[2] 2 (15%)	0·52
	#Total	-	[1] 1 (8%)	-	-	-	[1] 1 (8%)	-	[2] 2 (15%)	0·52
Gastrointestinal disorders	Nausea	-	[2] 2 (15%)	-	-	-	[1] 1 (8%)	-	[3] 3 (23%)	0·52
	#Total	-	[2] 2 (15%)	-	-	-	[1] 1 (8%)	-	[3] 3 (23%)	0·52
General disorders and administration site conditions	Chills	-	[2] 2 (15%)	-	-	-	[1] 1 (8%)	-	[3] 3 (23%)	0·52
	Fatigue	-	[5] 4 (31%)	-	-	[1] 1 (14%)	[3] 3 (23%)	[1] 1 (14%)	[8] 6 (46%)	0·33
	Hyperhidrosis	-	[1] 1 (8%)	-	[2] 2 (15%)	-	[1] 1 (8%)	-	[4] 4 (31%)	0·25
	Pyrexia	-	[3] 3 (23%)	-	[2] 2 (15%)	-	[2] 1 (8%)	-	[7] 5 (38%)	0·11
	#Total	-	[11] 5 (38%)	-	[4] 4 (31%)	[1] 1 (14%)	[7] 3 (23%)	[1] 1 (14%)	[22] 8 (62%)	0·07
Musculoskeletal and connective tissue disorders	Back pain	-	-	-	-	-	[1] 1 (8%)	-	[1] 1 (8%)	>0·9999
	Myalgia	[1] 1 (14%)	[2] 2 (15%)	-	-	-	[1] 1 (8%)	[1] 1 (14%)	[3] 3 (23%)	>0·9999
	#Total	[1] 1 (14%)	[2] 2 (15%)	-	-	-	[2] 2 (15%)	[1] 1 (14%)	[4] 4 (31%)	0·61
Nervous system disorders	Headache	[1] 1 (14%)	[12] 8 (62%)	[1] 1 (14%)	[2] 2 (15%)	[1] 1 (14%)	[4] 4 (31%)	[3] 2 (29%)	[18] 9 (69%)	0·16
	#Total	[1] 1 (14%)	[12] 8 (62%)	[1] 1 (14%)	[2] 2 (15%)	[1] 1 (14%)	[4] 4 (31%)	[3] 2 (29%)	[18] 9 (69%)	0·16
Respiratory, thoracic and mediastinal disorders	Tachypnoea	-	-	-	[1] 1 (8%)	-	-	-	[1] 1 (8%)	>0·9999
	#Total	-	-	-	[1] 1 (8%)	-	-	-	[1] 1 (8%)	>0·9999

In square brackets: number of events; number of patients with events; in brackets: percentage of patients with events.

Supplementary Figure 1

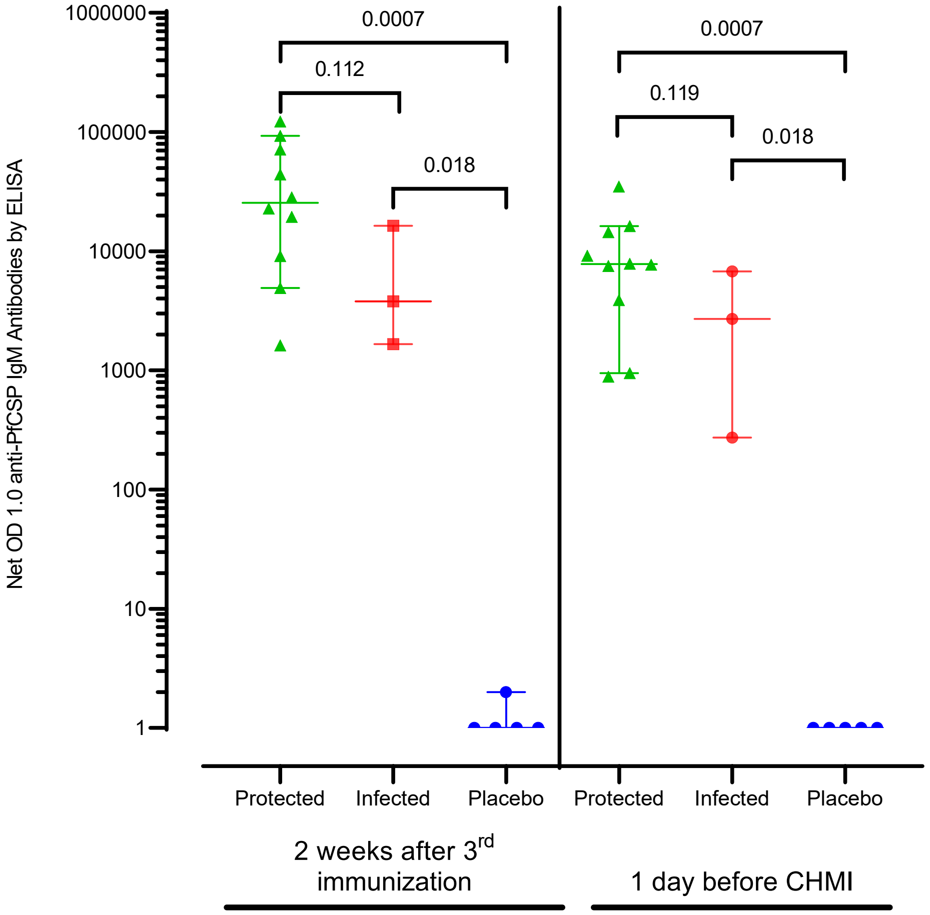

Supplementary Figure 1: IgM antibody reactivity against PfCSP. IgM antibodies specific to PfCSP were measured by ELISA using serum from volunteers before the first immunization (D-1), 15 days after the last immunization (D 44), as well as one day before CHMI (C -1). The net serum dilution (timepoint minus baseline) at which the optical density (OD) was 1·0 is plotted for each subject. Green triangles: Vaccine (protected); red squares: Vaccine (unprotected); blue circles: placebo controls. Represented data are derived from a single experiment with three technical replicates. Lines shown are median with 95% CI. N = 18 biologically independent study participants. Lines shown are median with 95% CI. P-values were estimated by two-sided Wilcoxon-Mann-Whitney test.

Supplementary Figure 2

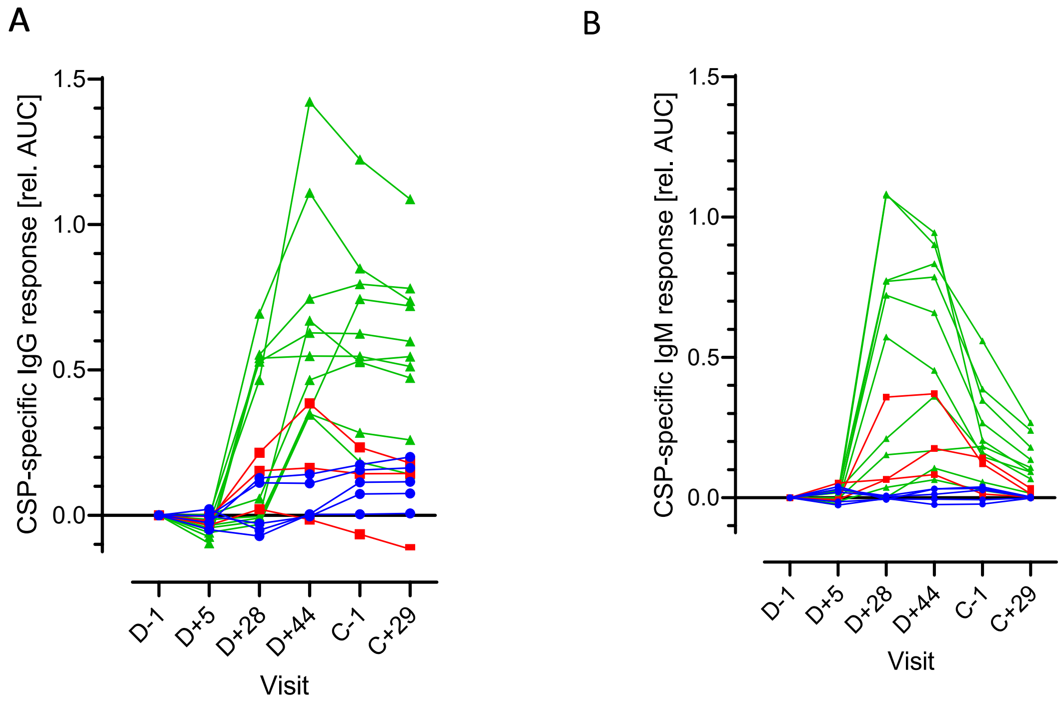

Supplementary Figure 2. PfCSP-specific antibody response during immunization and challenge phase. IgG (A) and IgM (B) antibody titers specific to PfCSP were measured by ELISA using serum from volunteers before each immunization (D-1, D+5, D+28), 15 days after the last immunization (D+44), as well as one day before challenge (C-1) and four weeks after challenge (C+29). Two-fold serum dilutions starting at 1:400 up to 1:51,200 were applied onto the ELISA plates, the area under the curve (AUC) was calculated. The presented data are baseline corrected values relative to a positive control sample pool. Green triangles: Vaccine (protected); red squares: Vaccine (unprotected); blue circles: placebo controls.

Supplementary Figure 3

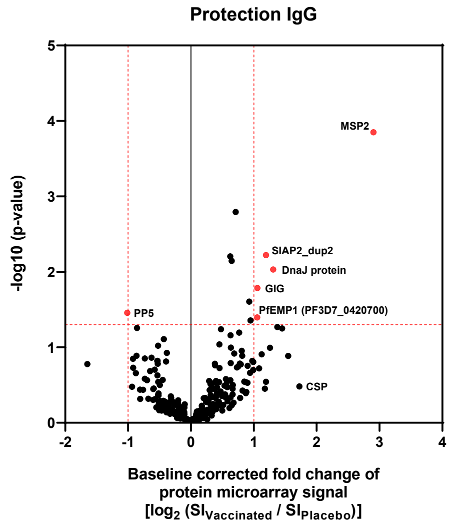

Supplementary Figure 3. IgG antibody reactivity against sporozoite, liver stage and blood stage proteins as quantified by protein microarray. Sera from all volunteers collected before immunization (baseline, D-1) and one day before challenge (C-1) were applied on protein microarrays containing 262 *Plasmodium falciparum* proteins representing 228 unique antigens. Analysis was performed on C-1 data after subtraction of the individual baseline reactivity. Differentially recognized antigens (p-value < 0·05 and fold change > 2) are depicted in red. P-values were estimated using the two-sided Welch-corrected Student's t-test. Protection-associated antibodies were identified by comparison of antigen reactivity in protected (to the right) and unprotected volunteers (to the left) within the Vaccine group.

Supplementary Figure 4

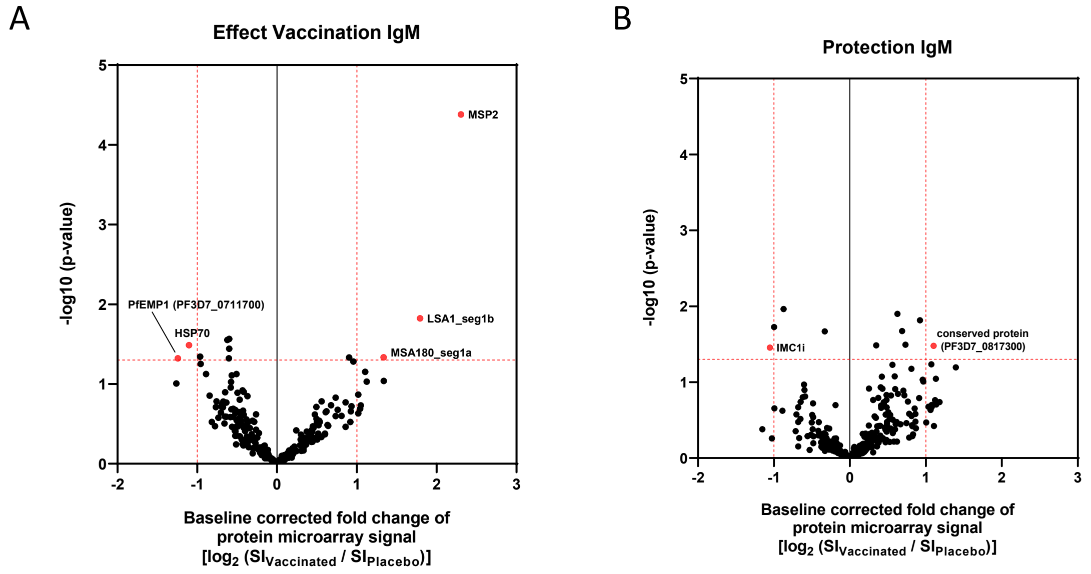

Supplementary Figure 4. IgM antibody reactivity against sporozoite, liver stage and blood stage proteins as quantified by protein microarray. Sera from all volunteers collected before immunization (baseline, D-1) and one day before challenge (C-1) were applied on protein microarrays containing 262 *Plasmodium falciparum* proteins representing 228 unique antigens. Analysis was performed on C-1 data after subtraction of the individual baseline reactivity. P-values were estimated using the two-sided Welch-corrected Student's t-test. (A) To estimate PfSPZ-CVac immunogenicity, antigen reactivity in vaccinated donors (to the right) was compared to the placebo controls (to the left). Differentially recognized antigens (p-value < 0·05 and fold change > 2) are depicted in red. (B) Protection-associated antibodies were identified by comparison of antigen reactivity in protected (to the right) and unprotected volunteers (to the left) within the vaccinated group.

Supplementary references

1. Mordmüller B, Surat G, Lagler H, et al. Sterile protection against human malaria by chemoattenuated PfSPZ vaccine. *Nature* 2017; **542**(7642): 445-9.

2. Barth S, Huhn M, Matthey B, Klimka A, Galinski EA, Engert A. Compatible-solute-supported periplasmic expression of functional recombinant proteins under stress conditions. *Appl Environ Microbiol* 2000; **66**(4): 1572-9.

3. Doolan DL, Mu Y, Unal B, et al. Profiling humoral immune responses to P. falciparum infection with protein microarrays. *Proteomics* 2008; **8**(22): 4680-94.

4. Silver JD, Ritchie ME, Smyth GK. Microarray background correction: maximum likelihood estimation for the normal-exponential convolution. *Biostatistics* 2009; **10**(2): 352-63.

5. McGee M, Chen Z. Parameter estimation for the exponential-normal convolution model for background correction of affymetrix GeneChip data. *Stat Appl Genet Mol Biol* 2006; **5**: Article24.

Study Reference: CVac-Tü3
Clinical Trial Protocol Version: Version 6.0
Date: 22 March 2018

Safety and protective efficacy of a simplified *Plasmodium falciparum* sporozoite Chemoprophylaxis Vaccine (PfSPZ-CVac) regimen in healthy malaria-naïve adults in Germany

EudraCT-Nr: 2018-004523-36

Trial Sponsor:
Universitätsklinikum Tübingen
represented by
Prof. Dr. Peter G. Kremsner

Principal Investigator:
Dr. Zita Sulyok

Modification historyVersion	Date	Authors
Version 1	03 AUG 2017	Z. Sulyok, B. Mordmüller, Peter G. Kremsner
Version 2	26 FEB 2018	S. Hoffmann, T. Richie
Version 3	13 MAR 2018	E.Saverino, A. Ruben, K. Walker
Version 4	30 NOV 2018	Z. Sulyok, M. Karnahl. B. Mordmüller, P. G. Kremsner
Version 5	18 DEC 2018	Z. Sulyok, M. Karnahl. B. Mordmüller, P. G. Kremsner
Version 6	22 MAR 2019	Z. Sulyok, M. Karnahl, B. Mordmüller, P. G. Kremsner

History of changes*

**since first execution - Protocol Version 4.0*

Version	Scope	Section	Details of changes
5.0	General Changes	Abbreviations	Unused abreviations were removed from the abbreviation list
5.0	Plasmodium Life Cycle	Figure 1	Figure 1 was replaced with a higher resolution image
5.0	Local Safety Monitor	Synopsis	Address inserted
5.0	Study Monitor,<br>SMC,<br>Pharmacovigilance	Synopsis	Person in charge and contact details inserted
5.0	**Additional Exploratory Objectives**	Synopsis<br>3.2 Trial Objectives<br>6.1.1 Squence of events<br>Table 8A,B<br>Annex 3	Additional objective inserted, Respective psychological assessment added in study procedures, Questionnaires for evaluation attached in Annex 3
5.0	AE coding	8 Assessment of Safety	Coding by MedDRA terminology defined
5.0	Safety reporting procedures were refined for AEs, SAEs and SUSARs	8.3 Reporting procedures for AEs<br>8.4 Reporting procedures for SAEs<br>8.4.1. Sponsors Assessment of the SAEs	1) Reporting extended to using email<br>2) Contact details for relevant safety departments added<br>3) Responsibilities for final AE grading were corrected: Sponsor and Sanaria will both perform second assessment<br>4) The term 'Sponsor' was extended to Sponsor or defined delegates
5.0	Pharmakovigillance	8.5.3. Pharmakovigillance	Reference to SAE manual inserted
5.0	Annual Safety Reporting	8.6. Annual Safety Report	Data lockpoint was further defined
6.0	Local Safety Monitor	Synopsis	Field of activity inserted for Local Safety Monitor
6.0	Rationale for Study Design	Synopsis	The Background information in this section was replaced by more concise information
6.0	References and Reference Numbering	2.4.1. Drugs for PfSPZ-CVac approach - chloroquine	The reference in this chapter had to be corrected, because of incorrect placement. By that the order

		and affecting all following chapters	changed and all references above number 33 were renumbered.
6.0	Figure 4 double insertion	2.6.10 Case Report	One of two identical copies of Figure 4 was removed
6.0	Treatment of *P.falciparum* infection	2.7.1. Potential risks	A dose definition of A/P treatment was inserted
6.0	Quantifciation of cardiac risk factors	5.4. Exclusion criteria	The range of this exclusion criteria was expanded from 5 to 10 years Quantification score was added to the reference list (#84).
6.0	Criteria for hospital admission	6.2.5. Antimalarial treatment	Decision about admission will be made by the investigator
6.0	Safety reporting procedures were refined for AEs, SAEs and SUSARs<br>SMC responsibility	8.4 Reporting procedures for SAEs<br>8.4.1. Sponsors Assessment of the SAEs<br>8.9. Safety monitoring committee and local safety monitor	5) Sanaria instead of Sponsor will be fully responsible for organizing and reporting to the SMC<br>6) Only related SAEs will be reported to the SMC<br>7) Timelines for reporting to the SMC were expanded
6.0	Statistical Analysis of Efficacy	9.5.4. Efficacy	Refined to control for TypeI error after second and third CHMI

Sponsor Representative Approval

Sponsor Representative: Peter G. Kremsner
Institut für Tropenmedizin

Signature	Date

Protocol Version 6.0 – Investigator agreement

"I have read this protocol and its amendment and agree to abide by all provisions set forth therein. I agree to comply with the principles of the International Conference on Harmonization Tripartite Guideline on Good Clinical Practice."

Principal Investigator: Zita Sulyok
Institut für Tropenmedizin

Signature	Date

Statistician: Benjamin Mordmüller
Institut für Tropenmedizin

Signature	Date

Confidentiality Statement

This document contains confidential information that must not be disclosed to anyone other than the trial Sponsor, the Investigator Team, and members of the Institutional Review Board. This information cannot be used for any purpose other than the evaluation or conduct of the clinical investigation without the prior written consent of the Principal Investigator.

Table of Contents

1. **General Information** 7
  1.1. Contact details 7
  1.2. Synopsis 8
  1.3. Abbreviations 12

2. **Background and Rationale** 14
  2.1. Epidemiology of malaria 14
  2.2. Lifecycle of the malaria parasite 14
  2.3. Malaria vaccines 15
    2.3.1. Malaria vaccines – general concepts 16
    2.3.2. Whole cell malaria vaccines 16
    2.3.3. Malaria chemoprophylaxis and in vivo attenuation 17
    2.3.4. PfSPZ Chemoprophylaxis Vaccine (PfSPZ-CVac) approach 17
  2.4. Drugs for PfSPZ-CVac approach 18
    2.4.1. Drugs for PfSPZ-CVac approach – chloroquine 18
  2.5. Microbial controlled infection studies of human volunteers 19
  2.6. Controlled human malaria infection (CHMI) 19
    2.6.1. CHMI – mosquito bites 20
    2.6.2. CHMI – PfSPZ Challenge (NF54 and 7G8) 21
    2.6.3. Characterization of PfSPZ Challenge (7G8) 26
    2.6.4. Conduct of CHMI trials 26
    2.6.5. Clinical presentation post CHMI 27
    2.6.6. Heterologous CHMI with PfSPZ Challenge (7G8) 28
    2.6.7. Ethical considerations of CHMI trials 29
    2.6.8. PfSPZ-CVac using PfSPZ Challenge (NF54) as the immunogen and CQ as the partner drug for chemoprophylaxis of PfSPZ 29
    2.6.9. Adverse events in malaria naïve adults 29
    2.6.10. Case Report (TÜCHMI-002; volunteer 083) 31
    2.6.11. PfSPZ-CVac immunization of malaria experienced adults in Africa. 33
  2.7. Summary of known and potential risks and benefits to human subjects of PfSPZ Challenge swith CQ for PfSPZ0-CVac 33
    2.7.1. Potential risks 33
    2.7.2. Potential benefits 35
  2.8. Rationale for conducting the trial 35
  2.9. Rationale of PfSPZ Challenge (NF54), PfSPZ Challenge (7G8), chloroquine dosing schedule 36
    2.9.1. Timing and dosing of PfSPZ Challenge (NF54) under chemoprophylaxis 36
    2.9.2. Dosing of PfSPZ Challenge (7G8) for CHMI 36
    2.9.3. Dosing of Chloroquine 36
  2.10. Rationale for route administration 36

3. **Trial objectives and purpose** 37
  3.1. Study overview 37
  3.2. Trial objectives 37
  3.3. Duration of the trial 37

4. **Trial design** 38
  4.1. Type and design of the trial 38
  4.2. Endpoints 38
  4.3. Minimization of bias 39
    4.3.1. Randomization 39
    4.3.2. Blinding and allocation concealment 39
  4.4. Treatments 39
    4.4.1. PfSPZ Challenge (NF54) for immunization 39
    4.4.2. Placebo 40

4.4.3. PfSPZ Challenge (7G8) for CHMI 40
4.4.4. PfSPZ Challenge product diluent 40
4.4.5. Minimizing environmental contamination with PfSPZ 41
4.4.6. Chloroquine 41
4.4.7. Rescue and other treatment 41
4.4.8. Supportive medications 42
4.4.9. Criteria for hospital admission 42

5. Selection and withdrawal of trial volunteers 43
5.1. Volunteers 43
5.2. Informed consent 43
5.3. Inclusion criteria 44
5.4. Exclusion criteria 44
5.5. Withdrawal of volunteers 45
5.6. Pregnancy 46

6. Trial methods and study procedures 46
6.1. Study procedures 46
6.1.1. Sequence of events 47
6.2. Observations 48
6.2.1. Blood sampling and laboratory tests 48
6.2.2. Administration of PfSPZ Challenge products 49
6.2.3. Administration of CQ 49
6.2.4. Malaria Diagnosis 53
6.2.5. Antimalarial treatment 53

7. Assessment of scientific objectives 56

8. Assessment of safety 56
8.1. Definitions 57
8.2. Causality assessment 59
8.3. Reporting procedures for AEs (Excluding SAEs) 59
8.4. Reporting procedures for SAEs 61
8.4.1. Sponsors Assessment of the SAEs 62
8.4.2. Follow-up of Initial Report 62
8.5. Reporting procedures for SUSARs 62
8.5.1. Report to the Investigator 63
8.5.2. Reporting to Safety Monitoring Committee 63
8.6. Annual Safety Report 63
8.7. Unblinding of the Medical Product 63
8.8. Procedures to be followed in the event of abnormal findings 64
8.9. Safety monitoring committee and local safety monitor 64
8.10. Holding and stopping rules 64
8.11. Examination and Report of Changes in the Risk to Benefit Ratio 65

9. Statistics 65
9.1. Sample size 65
9.2. Study population 66
9.2.1. Modified intention to treat population (mITT) 66
9.2.2. According to protocol population (ATP) 66
9.3. Randomization 66
9.4. Allocation concealment 66
9.5. Statistical methods 67
9.5.1. Demographics 67
9.5.2. Safety 67
9.5.3. Grading and causality assessment 67
9.5.4. Efficacy 67
9.6. Conduct of analyses 67
9.6.1. Blinding 67

9.6.2. Missing values 68
9.6.3. Immunological analyses 68

10. Quality control and quality assurance procedures 68
10.1. Investigator procedures 68
10.2. Monitoring 68
10.3. Modification to protocol 69
10.4. Protocol deviation 69
10.5. Audits 69
10.6. Trial progress 70

11. Ethics 70
11.1. Declaration of Helsinki 70
11.2. ICH guidelines for good clinical practice 70
11.3. Informed consent 70
11.4. Research ethics committee 70
11.5. Volunteer confidentiality 70
11.6. Volunteer consent withdrawal 71

12. Data handling and record keeping 71
12.1. Data handling 71
12.2. Record keeping 71
12.3. Use of samples, specimens and data 71
12.4. Sample/ Data storage 72
12.5. Disposition of samples, specimens and data at completion of the protocol 72
12.6. Source data and case report forms (CRFs) 72
12.7. Data protection 72
12.8. Reporting 72

13. Financing and Insurance 73
13.1. Financing 73
13.2. Insurance 73
13.3. Compensation of volunteers 73
13.4. Treatment costs 73

14. References 74

1. General Information

1.1. Contact details

**Title**	**Safety and protective efficacy of a simplified *Plasmodium falciparum* sporozoite Chemoprophylaxis Vaccine (PfSPZ-CVac) regimen in healthy malaria-naïve adults in Germany**
**Trial Sponsor study code**	**CVac-Tü3**
**EudraCT number**	2018-004523-36
**Principal Investigator**	Zita Sulyok<br>Universitätsklinikum Tübingen<br>Institut für Tropenmedizin<br>Wilhelmstraße 27<br>D-72074 Tübingen, Germany<br>Tel: +49 7071 2982188<br>email: zita.sulyok@uni-tuebingen.de
**Study Manager**	Matthias Karnahl<br>Universitätsklinikum Tübingen<br>Institut für Tropenmedizin<br>Wilhelmstraße 27<br>D-72074 Tübingen, Germany<br>Tel: +49 7071 29 82905<br>email: matthias.karnahl@uni-tuebingen.de
**Sponsor**	Universitätsklinikum Tübingen<br>Geissweg 3<br>D-72076 Tübingen
**Sponsor representative**	Peter G. Kremsner<br>Universitätsklinikum Tübingen<br>Institut für Tropenmedizin<br>Wilhelmstraße 27<br>D-72074 Tübingen, Germany<br>Tel: +49 7071 2987179<br>email: peter.kremsner@uni-tuebingen.de
**PfSPZ Challenge Manufacturer**	Sanaria Inc.<br>9800 Medical Center Dr<br>Rockville, MD 20850<br>United States of America<br>Tel: +1 301 7703222<br>email: slhoffman@sanaria.com
**Trial site**	Eberhard Karls Universität Tübingen<br>Universitätsklinikum Tübingen<br>Institut für Tropenmedizin<br>Wilhelmstraße 27<br>D-72074 Tübingen, Germany
**Local Safety Monitor**	Prof. Dr. Michael Ramharter<br>(Tropical medicine, Clinical Research Department, Bernhard Nocht Centre for Clinical Trials, BNITM)<br>Bernhard-Nocht-Straße 74<br>D-20359 Hamburg

	Tel.: 040 4281 8511<br>email: ramharter@bnitm.de
**Study Monitor**	Dr. Urs Egner<br>An den Deichwiesen 21a<br>30659 Hannover<br>Tel.: 0178 5124340<br>email: urs@origio.eu
**Safety Monitoring Committee**	**1. SMC Chair**<br>Alan Cross, MD (Professor, University of Maryland School of Medicine)<br>655 W. Baltimore Street<br>Baltimore, MD 21201<br>Email: across@medicine.umaryland.edu<br>Office Phone: (410) 706-5328<br>Cell Phone: (240) 350-5893<br>**2. SMC Member**<br>Kent Kester, MD<br>Vice President and Head, Translational Science & Biomarkers<br>Sanofi Pasteur<br>One Discovery Drive<br>Swiftwater, PA 18370<br>Email: kent.kester@sanofipasteur.com<br>Office Phone: (570) 957-1924<br>Cell Phone: (570) 350-4492<br>**3. SMC Member (Local Safety Monitor)**<br>Michael Ramharter, MD<br>(Professor, Clinical Research Department, BNITM)
**Pharmaco-vigilance**	Benjamin Mordmüller<br>Universitätsklinikum Tübingen<br>Institut für Tropenmedizin<br>Wilhelmstraße 27<br>D-72074 Tübingen, Germany<br>email: benjamin.mordmueller@uni-tuebingen.de

1.2. Synopsis

**Title**	**Safety and protective efficacy of a simplified *Plasmodium falciparum* sporozoite Chemoprophylaxis Vaccine (PfSPZ-CVac) regimen in healthy malaria-naïve adults in Germany**
**Study ID**	**CVac-Tü3**
**EudraCT number**	2018-004523-36
**Protocol version**	6.0
**Protocol date**	22 March 2019
**Trial Centre**	Eberhard Karls Universität Tübingen<br>Universitätsklinikum Tübingen<br>Institut für Tropenmedizin<br>Wilhelmstraße 27<br>72074 Tübingen, Germany

**Design**	Single center, randomized, placebo-controlled, double-blind, PfSPZ Challenge with chloroquine (PfSPZ-CVac) trial with repeat controlled human malaria infection (CHMI).
**Development phase**	1
**Population**	Healthy malaria-naïve adults aged between 18 and 45 years.
**Rationale for Study Design**	**Purpose**<br>Establish a regimen of three injections of intravenous PfSPZ Challenge (NF54) under single dose chloroquine chemoprophylaxis administered at Days 0, 5 and 28, an immunization approach called PfSPZ-Chemoprophylaxis Vaccine (PfSPZ-CVac), which:<br><br>1. Is safe and well tolerated.<br>2. Provides consistent sterile protection against repeat CHMI with PfSPZ (7G8) in healthy adult subjects 10 weeks, 26 weeks and 52 weeks following immunization.<br>3. Is a practical preventive regimen for malaria.<br><br>**Background**<br>Availability of a highly efficacious malaria vaccine would complement current malaria control programs and enable the development of large-scale elimination strategies. The only way to consistently achieve complete protection in >90% of cases against CHMI with Plasmodium falciparum (Pf) is by immunization with whole PfSPZ, attenuated either by irradiation (PfSPZ Vaccine) or with chemoprophylaxis (PfSPZ-CVac). Sanaria Inc., a biotechnology company has developed aseptic, purified cryopreserved PfSPZ products that allows an accurate and practical way of dosing sporozoites and conduct CHMI trials in a standardized manner.<br><br>In our previous PfSPZ-CVac study (TÜCHMI-002), we showed that IV administration of three doses of 5.12x104 PfSPZ of PfSPZ Challenge over 8 weeks to volunteers taking CQ chemoprophylaxis protected 100% (9/9) of study participants 10 weeks following immunization, whereas immunization with $3.2x10^3$ or $1.28x10^4$ PfSPZ resulted in 33% (3/9) and 67% (6/9) protective efficacy, respectively. However, this CQ regimen is sub-optimal for a routine setting, since it must be ensured that CQ is taken weekly for a total of at least 10 doses. Condensed immunization regimens requiring fewer doses of CQ would be more practical. With this in mind, in the TÜCHMI-002 trial, we showed that 3 doses of $5.12x10^4$ PfSPZ of PfSPZ Challenge at 5 or 14 day intervals protected 63% (5/8) and 67% (6/9) of volunteers respectively, indicating that shorter regimens with fewer doses of CQ could be used, although potentially at the cost of reduced efficacy.<br>Building on these data, the aim of this current study is to further evaluate condensed immunization regimens of PfSPZ-CVac using three concurrent administrations of PfSPZ Challenge and chloroquine at closely spaced intervals and achieve cross strain heterologous immunity. We will retest the 5-day interval, but aim to improve efficacy by (1) increasing the dose of PfSPZ, and (2) by delaying the third dose from the day + 10 to + 28. Thus we will use doses of $1.1x10^5$ PfSPZ together with 10mg/kg of chloroquine, on Days 0, 5 and 28.<br>To assess the efficacy, CHMI will be used which is a well estabilished tool in novel malaria vaccines and drug trials. As CHMI trials are carried out in a

	controlled environment, they allow unprecedented detailed evaluation of parasite growth and immunological responses, providing essential information for vaccine and drug development.
**Study design**	Single center, randomized, placebo-controlled, double-blinded trial using PfSPZ Challenge (NF54) under chloroquine chemoprophylaxis for immunization and PfSPZ Challenge (7G8) for repeat CHMI.<br><br>A total of 21 adult, healthy, malaria-naïve volunteers will receive three injections by direct venous inoculation (DVI) of either placebo (n = 7) or 1.1×10^5 PfSPZ of PfSPZ Challenge (NF54) (n = 14) under chemoprophylaxis with chloroquine (10mg/kg) on Days 0, 5 and 28. The placebo will be 0.9% NaCl (normal saline).<br><br>Ten weeks after the last immunization, volunteers will undergo CHMI with 3.2×10^3 PfSPZ of PfSPZ Challenge (7G8) and will be followed until asexual blood stage parasitemia is detected by quantitative real time PCR (qPCR) or thick blood smear microscopy. If parasitemic, they will be treated with atovaquone/proguanil (A/P) used as a standard treatment regimen. In the event of no parasitemia, volunteers will be followed until +28 days post-CHMI and will not receive A/P.<br><br>Twenty-six and fifty-two weeks after the last immunization, a second and a third CHMI with 3.2×10^3 PfSPZ of PfSPZ Challenge (7G8) will be administered by DVI to assess longevity of protection. All volunteers will be followed up to 28 days post-inoculation. Those developing parasitemia will be treated with A/P.
**Groups**	**Group A:** Verum (n = 14)<br>**Group B:** Placebo* (n = 7)<br><br>*Placebo: 0.9% NaCl (normal saline) with chloroquine regimen (3x10mg/kg)
**Holding and stopping rules**	The study may be placed on safety hold for the following reasons:<br>• On advice of the safety monitor.<br>• On advice of the investigators.<br>• On advice of the ethics committee or the safety monitoring committee (SMC).<br>• One or more participants experience an unexpected serious adverse event (SAE) that is determined to be related to study product (PfSPZ Challenge) administration.<br>• One or more participants experience an unexpected SAE that is determined to be related to an antimalarial.<br>• Two or more of the same grade 3-4 adverse events in the same group of subjects which are unexpected and definitely related to study product administration.
**Statistics and Sample Size**	The objectives of this study are to assess safety, tolerability and protective efficacy against heterologous CHMI, of the PfSPZ-CVac approach with three doses of PfSPZ Challenge (NF54) and CQ chemoprophylaxis.<br><br>Safety and tolerability data will be presented as descriptive analyses, as listings and graphically. No formal hypothesis testing for safety and tolerability will be done. The rate and magnitude of AEs in volunteers receiving PfSPZ plus CQ will be compared to the rate and magnitude of AEs in controls receiving NS plus CQ. Based on pharmacokinetic data in peripheral blood it is not expected that chloroquine will have a measurable effect on development of parasitemia during immunization, but will eliminate all asexual erythrocytic stage parasites.

	100% of 80 non-immunized American, German, and Spanish volunteers administered $3.2x10^3$ PfSPZ of PfSPZ Challenge (NF54) by DVI have become infected and 21/22 administered $3.2x10^3$ PfSPZ of PfSPZ Challenge (7G8) have become infected. Thus, all or nearly all of the normal saline controls in this study should become infected.<br>Hence, the lower border of the 95% confidence interval of infectivity is 95%. To be able to show, with a power of 80% and a two-tailed alpha of 5%, that 25% or less of immunized volunteers and 85% of controls, allocated in a 2:1 ratio become infected, 14 immunized and 7 placebo-treated volunteers per group are required.
**Follow-up duration**	105 days after last CHMI.
**Planned Trial Period**	71 weeks.<br>Approximate trial duration for individual volunteers: 71 weeks.
**Primary Objectives**	1. Establish that an immunization regimen of three injections of PfSPZ Challenge by DVI and oral chloroquine chemoprophylaxis administered on Days 0, 5 and 28, is safe and well tolerated.
**Secondary Objective**	1. Establish that an immunization regimen of PfSPZ Challenge under chloroquine chemoprophylaxis, provides protection against repeat CHMI in healthy adult subjects 10 (primary VE outcome), 26, and 52 weeks following the last immunization.
**Exploratory Objectives**	1. Identify parasitological and immunological correlates of protection against CHMI.<br>2. Characterize B- and T-cell responses to immunization and repertoire.<br>3. Assess parasite kinetics following CHMI.<br>4. Identify psychological changes of the volunteers during the study period
**Endpoints**	**Safety endpoints**<br>Number or occurrence of at least possibly related Grade 3-4 AEs and SAEs from time of first CQ administration to the end of the follow-up period.<br>**Efficacy endpoint**<br>Proportion of protected volunteers. Protection is defined as the absence of parasites in the peripheral blood for +28 days following CHMI with PfSPZ Challenge (7G8) in volunteers receiving PfSPZ-CVac. Parasitemia is defined as at least one qPCR result above 100 parasites per mL among three positive results at least 12 hours apart or as a positive thick blood smear after CHMI with PfSPZ Challenge. Statistical testing is done hierarchically: 1) protection against first CHMI, 2) protection against second CHMI, 3) protection against third CHMI.<br>**Exploratory endpoints**<br>• Time-to-parasitemia in volunteers who receive immunization using PfSPZ Challenge or placebo under chloroquine chemoprophylaxis and become parasitemic within +28 days following CHMI with PfSPZ Challenge (7G8). Statistical testing is done hierarchically: 1) time-to-parasitemia in first CHMI, 2) time-to-parasitemia in second CHMI and 3) time-to-parasitemia in third CHMI.<br>• Time-to-parasitemia in placebo recipients following second versus first CHMI and third versus second CHMI (carry-over effect).

**Investigational products**	Aseptic, purified, vialed, cryopreserved, infectious *P. falciparum* sporozoites, strain NF54, produced by Sanaria Inc. – Sanaria® PfSPZ Challenge (NF54).<br><br>Aseptic, purified, vialed, cryopreserved, infectious *P. falciparum* sporozoites, clone 7G8, produced by Sanaria Inc. – Sanaria® PfSPZ Challenge (7G8).<br><br>**Immunization:** PfSPZ Challenge (NF54) under chemoprophylaxis with chloroquine (Sanaria® PfSPZ-CVac)<br><br>**CHMI:** Sanaria® PfSPZ Challenge (7G8)
**Form**<br><br><br><br><br>**Route of Administration**<br><br><br><br><br><br><br><br><br><br>**Dose per Administration**	PfSPZ Challenge (NF54) (for PfSPZ-CVac approach): Liquid<br>PfSPZ Challenge (7G8) (for CHMI): Liquid<br>0.9% NaCl (normal saline) (placebo): Liquid<br>Chloroquine: Tablets<br><br>PfSPZ Challenge (NF54) (for PfSPZ-CVac approach): Injection into a superficial vein of the arm (direct venous inoculation = DVI)<br>PfSPZ Challenge (7G8) (for CHMI): Injection into a superficial vein of the arm (DVI)<br>NaCl 0.9% (placebo): Injection into a superficial vein of the arm (DVI)<br>Chloroquine: Oral<br><br>PfSPZ Challenge (NF54) for PfSPZ-CVac approach: $1.1x10^5$ PfSPZ<br>PfSPZ Challenge (7G8) for CHMI: $3.2x10^3$ PfSPZ<br><br>Antimalarial drugs:<br>*Chemoprophylaxis:* Tablets (155 mg chloroquine base) administered as a single dose on the days of immunization, each dose consisting of 10mg/kg chloroquine base (Bayer) to a maximum of 620 mg.<br>*Treatment following CHMI:* First line treatment after CHMI is atovaquone/proguanil as per package insert. Second line treatment is artemether/lumefantrine as per package insert.

1.3. Abbreviations

AE	Adverse Event
ALT	Alanine Aminotransferase
AST	Aspartate Aminotransferase
BUN	Blood Urea Nitrogen
CHMI	Controlled Human Malaria Infection
CBC	Complete Blood Count
CDT	Carbohydrate-Deficient Transferrin
CPS	Chemoprophylaxis with sporozoites
CRF	Case Report Form
CQ	Chloroquine base
DVI	Direct Venous Inoculation
EC	Ethics Committee
ECG	Electrocardiography
FDA	US Food and Drug Administration
GCP	Good Clinical Practice
GM	Geometric mean
GMP	Good Manufacturing Practice

HBsAg	Hepatitis B Surface Antigen
HCG	Human Chorionic Gonadotropin
HCV	Hepatitis C Virus
HIV	Human Immunodeficiency Virus
HAS	Human Serum Albumin
ICH	International Conference on Harmonization of Technical Requirements for Registration of Pharmaceuticals for Human Use
ID	Intradermal
IM	Intramuscular
ISI	Inhibition of Sporozoite Invasion
IV	Intravenous
LDH	Lactate Dehydrogenase
LSM	Local Safety Monitor
MRI	Magnetic Resonance Imaging
NaCl	Sodium chloride
PBS	Phosphate Buffered Saline
PCR	Polymerase Chain Reaction
PEI	Paul-Ehrlich-Institute
P. falciparum, Pf	*Plasmodium falciparum*
PfSPZ	*Plasmodium falciparum* sporozoites
PfSPZ Challenge (NF54)	Aseptic, purified, cryopreserved *P. falciparum* sporozoites, strain NF54
PfSPZ Challenge (7G8)	Aseptic, purified, cryopreserved *P. falciparum* sporozoites, clone 7G8
PfSPZ-CVac	PfSPZ Challenge administered with a chemoprophylactic antimalarial
PfSPZ Vaccine	Aseptic, purified, irradiated, cryopreserved *P. falciparum* sporozoites
PI	Principal Investigator
Q1, Q2, Q3, Q4	1st, 2nd, 3rd, 4th quarter of the year
qPCR	Quantitative Polymerase Chain Reaction
RTS,S	Malaria vaccine candidate
SAE	Serious Adverse Event
SMC	Safety Monitoring Committee
SmPC	Summary of Product Characteristics
SOP	Standard Operating Procedure
SPZ	Sporozoites
SUSAR	Suspected Unexpected Serious Adverse Reaction
UKT	Universitätsklinikum Tübingen
USMMVP	US Military Malaria Vaccine Program
WHO	World Health Organization

2. Background and Rationale

2.1. Epidemiology of malaria

Although recent and encouraging evidence suggests that the epidemiology of Pf malaria is changing across certain parts of Africa[1], the worldwide burden of disease from malaria remains a major public health problem, with a WHO estimate of 212 and 216 million clinical episodes and 429 000 and 445,000 deaths from malaria in 2015 and 2016 respectivley[2,3]. By far the major burden is in Africa and the enormous economic and social consequences of malaria have been well documented[4].

Despite an investment by the international community of an estimated US$ 2.7 billion in malaria control interventions in 2016, the numbers of cases and deaths reported by WHO increased in 2016 as compared to 2015. There are many factors responsible for the reduced impact of interventions on malaria. One is the development of resistance both in *Anopheles* mosquitoes to certain insecticides and of malaria parasites to chemotherapeutic agents. These and other factors have contributed to an increasing need for new, effective interventions for the prevention or treatment of malaria[5]. Malaria vaccines may have a particularly large impact on malaria epidemiology, especially if they provide sustained protection[6] and interrupt transmission[7].

2.2. Lifecycle of the malaria parasite

The lifecycle of the parasites that cause malaria in humans is complex, with stages in both human and mosquito hosts (Figure 1). The bites of infected female *Anopheles* mosquitoes transmit malaria sporozoites (SPZ) to the human host [1][a] where they travel via the bloodstream to the liver and invade hepatocytes [2, liver-stage]. Here, they mature into merozoites for 5.5 to 7.5 days [3] after which the hepatocytes release a large number of merozoites into the bloodstream [4]. Merozoites then invade erythrocytes where they multiply and after two days egress from the erythrocyte, releasing progeny merozoites that in turn invade new erythrocytes [5, blood-stage]. This results in a feed forward loop with exponential growth of the parasite population until the immune system or metabolic resource-restriction dampens amplification. A small percentage of merozoites differentiate into gametocytes [6], which, when ingested by a mosquito, mature to gametes. Subsequently, gametes of the opposite sex unite to create zygotes [7, 8]. The zygote matures to an ookinete and then an oocyst, which releases sporozoites that migrate to the mosquito's salivary glands [9] and are injected into the human host when the mosquito feeds [10]. Sporozoites, gametocytes, and liver-stage of malaria parasites do not induce pathology, symptoms or signs of malaria. It is the asexual blood-stage of infection that is associated with symptoms and potentially severe or fatal complications. The PfSPZ-CVac approach is a pre-erythrocytic vaccination strategy to induce an immune response that disrupts the cycle before asexual blood-stage [5] amplification, thereby preventing clinical illness.

[a] Numbers in square brackets correspond to labels in Figure 1.

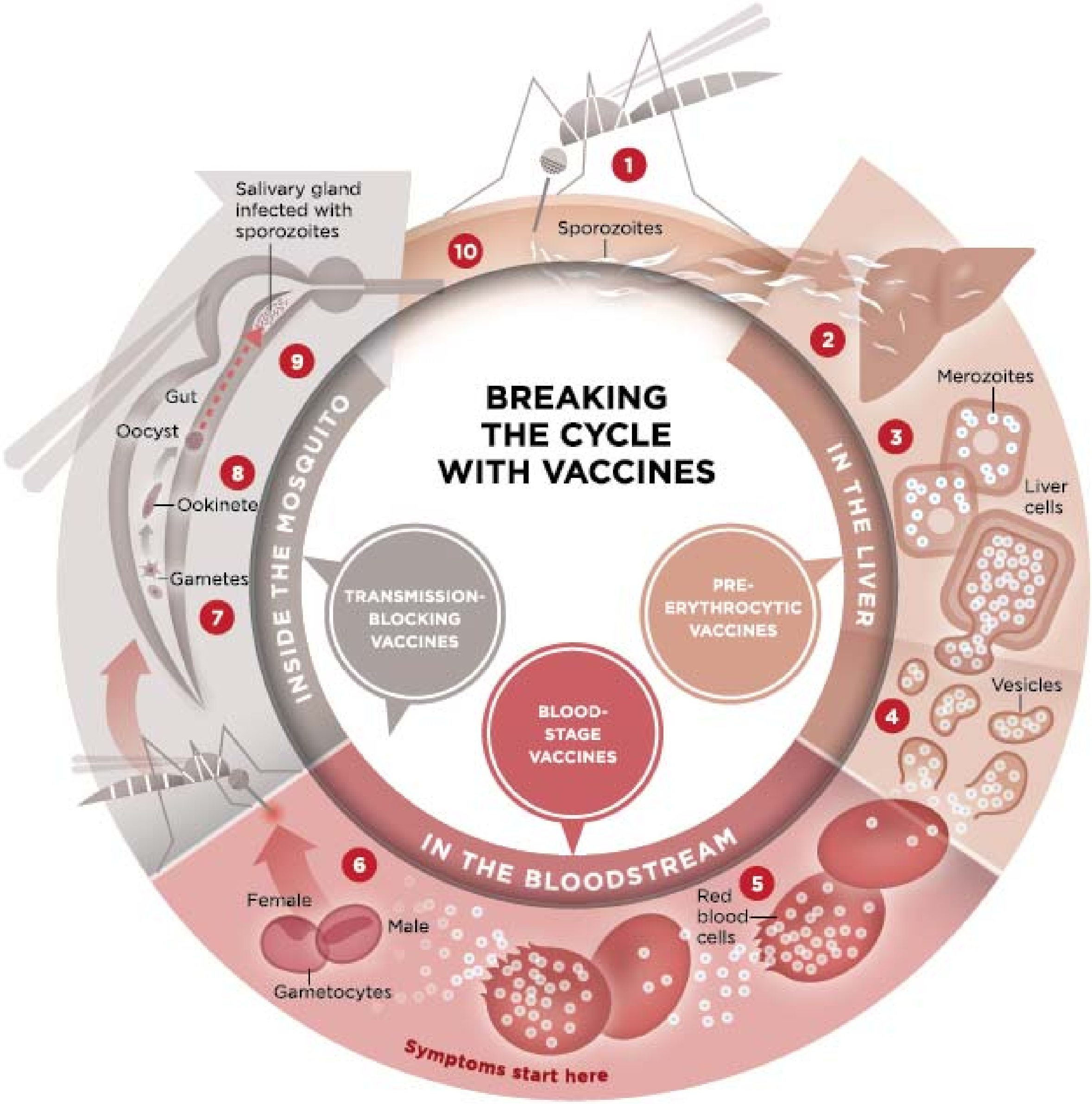

Figure 1: Lifecycle of malaria highlighting the three major stages that are targets for vaccine-development (from: malaria vaccine initiative; www.malariavaccine.org)

2.3. Malaria vaccines

A malaria vaccine would be the first anti-parasitic vaccine for human use. The history of attempts to develop such a vaccine dates back to 1910, when the first vaccination experiments using attenuated sporozoites were reported[8]. Highly efficacious vaccines may contribute enormously to disease control but for current malaria vaccine candidates an integrated approach is more likely to result in substantial health benefits[6,9] . Most malaria vaccine candidates in advanced clinical development have failed to show high efficacy or efficacy at all. A notable exception is RTS,S, a pre-erythrocytic vaccine candidate that completed phase 3 testing and has now moved to pilot implementation projects. It showed 56% clinical efficacy (time to event assessment of clinical cases prevented) in a first analysis[10] and then 31% clinical efficacy when integrated into the expanded program on immunization schedule for infants.[11] It received a positive scientific opinion by the European Medicine Agency under Article 58 on 24 July 2015. Other vaccine candidates in clinical trials in their respective target populations include those designed to mimic naturally acquired immunity against asexual blood-stage parasites. Those vaccine antigens include sequences from merozoite proteins 1, 2, and 3[12-14], GMZ2[15-17] , and apical membrane protein 1[18].

2.3.1. Malaria vaccines – general concepts

Administration of a vaccine can be considered as a way of communication with a future pathogen through a time channel. The information contained in the vaccine is coded, stored by the immune system and released, together with an anti-parasitic response upon encounter with the pathogen. To induce a successful anti-parasitic reaction, the vaccine needs to contain information about the pathogen without being pathogenic itself. One way to achieve this is to 'attenuate' the pathogen itself, either by serial passage, chemical, or physical treatment. In some cases inert pathogens or components thereof work as well. The most radical form of attenuation is to use recombinant or synthetic mimics of the pathogen. In this case additional, non-specific compounds that induce an accompanying innate immune response, so called adjuvants, are usually required. Most malaria vaccine candidates in advanced clinical development follow the strategy of combining a recombinant protein with an adjuvant. So far efficacy of such malaria vaccine candidates was not measurable or comparatively low (0 – 60%) when compared to whole cell vaccines. The updated malaria vaccine technology roadmap of the World Health Organization (WHO) defined the strategic goal to develop a "malaria vaccine with protective efficacy of at least 75% against clinical malaria suitable for administration to appropriate at-risk groups in malaria endemic areas"[19] In CVac-Tü3 a live whole cell vaccination approach that meets the criterion of at least 75% vaccine efficacy against homologous and heterologous parasite strains is pursued by multiple injections of PfSPZ Challenge (NF54) under antimalarial chemoprophylaxis. In this case, however, the vaccine is intended to induce sterile immunity, preventing any parasitemia (and not just clinical illness). By completely preventing malaria, the vaccine will also prevent ongoing transmission, and therefore would qualify as a vaccine to interrupt malaria transmission (VIMT).

2.3.2. Whole cell malaria vaccines

The first successful attempts to develop a malaria vaccine were achieved by attenuation of live parasites, both pre-erythrocytic [8,20] and erythrocytic[21] developmental stages. Pre-erythrocytic parasites were mostly attenuated by irradiation (UV, X-ray, radioactive cobalt or cesium), whereas asexual erythrocytic vaccines were completely inactivated or produced recombinantly and combined with adjuvants. Due to safety concerns about the adjuvant, development of erythrocytic whole cell vaccines with inactivated parasites was discontinued. However, it was reported that repeated very low dose infection with asexual blood-stage parasites and subsequent treatment induces a protective immune response against intravenously administered blood stage parasites[21]. After confirmation of the protective efficacy of attenuated sporozoites in various animal models[22,23]pre-erythrocytic vaccine development was quickly translated into human trials, where the principle was proven when more than 1,000 bites of -irradiated infectious mosquitoes to immunize human volunteers were used[24,25]. Subsequent experiments confirmed these findings and showed strain transcending immunity[25,26] , which is an essential feature of a vaccine to be used for malaria control.

Besides irradiation, genetic attenuation is possible and currently in early clinical development. Here, *Plasmodium* parasites are genetically manipulated, so that they can complete the normal pre-erythrocytic cycle but lack the ability to progress to the blood-stage due to inactivation of liver-stage relevant genes[27]. The current focus of research on genetically attenuated sporozoites is on better immunogenicity, improved protection[28] and securing the system from breakthrough infections[29].

Irradiation of sporozoites within mosquitoes and application of 1,000 or more bites during multiple immunization sessions is obviously very far from a marketable pharmaceutical product. To translate these findings into a product, Sanaria Inc. developed a technology to produce aseptic, purified, vialed, cryopreserved, fully infectious NF54 *P. falciparum* sporozoites (PfSPZ) that can be administered through injection (PfSPZ Challenge (NF54)) and a second product: aseptic, purified, vialed, cryopreserved, radiation-attenuated NF54 PfSPZ that can also be administered through injection (PfSPZ Vaccine) to induce protection similar to irradiated mosquitoes. PfSPZ Vaccine showed limited efficacy when administered by the intradermal and subcutaneous routes[30]. Animal

studies showed good immunogenicity of the same product upon intravenous (IV) application. Hence a second trial using this route of administration with doses up to 135,000 PfSPZ was conducted (ClinicalTrials.gov Identifier: NCT01441167), and the vaccine was shown to be highly protective against subsequent controlled human malaria infection (CHMI)[31]. PfSPZ Vaccine has subsequently been shown to be protective against CHMI with parasites the same as in the vaccine (homologous) and with parasites that are different than in the vaccine (heterologous) and against intense transmission of heterogeneous Pf parasites in the field in Africa[32,33]. Protection has been demonstrated for at least 14 months against CHMI and 6 months against natural transmission in the field[34,35] .

Another way of attenuating malaria parasites is to inoculate volunteers receiving antimalarial chemoprophylaxis with fully infectious parasites. In a first trial, volunteers received 3 immunizations with 12-15 infectious mosquito bites while concurrently receiving chloroquine (CQ). All volunteers were protected against subsequent CHMI, and 4/6 were still protected 28 months after immunization. This approach, called chemoprophylaxis with sporozoites (CPS), is the model for the PfSPZ-Chemoprophylaxis Vaccine (PfSPZ-CVac) approach.[36-39]

CHMI by PfSPZ Challenge (NF54) was safe in all 711 volunteers exposed as of January 1, 2018. 483 of these volunteers participated in the CHMI studies listed in section 2.6. 94 volunteers were administered PfSPZ Challenge (NF54) used to assess efficacy of the PfSPZ-CVac and 134 received PfSPZ Challenge (NF54) to assess efficacy of PfSPZ Vaccine. In addition, CHMI by PfSPZ Challenge (7G8) was safe in all 34 volunteers exposed so far.

The clinical picture of PfSPZ Challenge-mediated malaria is identical to mosquito-mediated CHMI and IV injection of $3.2x10^3$ PfSPZ has become the gold standard for CHMI with PfSPZ Challenge in malaria-naïve individuals in many centers.

2.3.3. Malaria chemoprophylaxis and *in vivo* attenuation

This approach towards attenuation involves infection with competent (non-attenuated) sporozoites (e.g. PfSPZ Challenge) under chemoprophylaxis that is directed against asexual blood or liver-stages only. It allows development of a successful immune response against the clinically silent pre-erythrocytic stage and inhibition of erythrocytic development through the antiparasitic action of a drug at liver-stage or early asexual blood-stage. Since parasite virulence is suppressed by chemotherapy only after inoculation, this approach is a form of *in vivo* attenuation. Proof-of-concept studies in mice [39] were followed by clinical trials more than 30 years later [36-38]which showed complete protection upon subsequent CHMI.

2.3.4. PfSPZ Chemoprophylaxis Vaccine (PfSPZ-CVac) approach

The PfSPZ-CVac approach is a new method of CPS, which uses PfSPZ Challenge instead of infected mosquito bites for immunization.

Protective efficacy of PfSPZ-CVac (CQ). In the TÜCHMI-002 trial, PfSPZ Challenge (NF54) was used for immunization under CQ chemoprophylaxis. The immunizing dose of $5.12x10^4$ PfSPZ administered intravenously three times at four-week intervals achieved 100% protection against homologous CHMI by DVI with $3.2x10^3$ PfSPZ in TÜCHMI-002 Stage A[40]. In TÜCHMI-002 Stage B, immunization schedules with $5.12x10^4$ PfSPZ administered three times at two-week and at five-day intervals were evaluated, which resulted in 67% (6/9) and 63% (5/8) protective efficacy at 10 weeks, respectively[40]. It has subsequently been shown that $5.12x10^4$ PfSPZ of PfSPZ Challenge (NF54) administered three times at four-week intervals with CQ chemoprophylaxis (same as best regimen in the TÜCHMI-002 trial) resulted in 80% (4/5) protection against homologous CHMI at 10 weeks after the last dose of PfSPZ Challenge (Healy, unpublished), that $2.0x10^5$ PfSPZ of PfSPZ Challenge also administered three times at four-week intervals with CQ chemoprophylaxis resulted in 100% (3/3) protection against heterologous CHMI at 12 weeks (Mwakingwe, unpublished), and that $1x10^5$ PfSPZ administered three times at five-day intervals with CQ chemoprophylaxis resulted in 75% (6/8) protection against homologous CHMI at 9 weeks after the last dose of PfSPZ Challenge (Jackson, unpublished). An alternative antimalarial

with liver stage (as well as blood stage) activity against *Plasmodium*, pyrimethamine, can also be used successfully at the partner drug for PfSPZ-CVac, as recently demonstrated in a trial conducted by NIH in Bethesda. Three DVI administrations of $2.0x10^5$ PfSPZ of PfSPZ Challenge at four-week intervals in combination with pyrimethamine 50 mg administered on days +2 and +3 after each injection protected 7/8 (87.5%) participants against homologous CHMI conducted at 12 weeks and 4/4 (100%) against heterologous (7G8) CHMI, also conducted at 12 weeks (Mwakingwe, unpublished).

Safety of PfSPZ-CVac (CQ) in non-immunes. The safety data from the TÜCHMI-002 trial has been published, and there were no significant differences in AEs between vaccinees and normal saline controls. However, in the subsequent studies there have been self-limited grade 3 adverse events during the period of transient parasitemia, particularly at higher doses of PfSPZ or when 7 day intervals were used between immunizations (see below). Because of these findings in an ongoing trial in Bethesda, MD in which $2.0x10^5$ PfSPZ of PfSPZ Challenge (NF54) is being administered three times at 4-week intervals with CQ chemprophylaxis, ibuprofen or naproxen have been administered for approximately 24 hours beginning on the 7th day after injection of PfSPZ Challenge. After the first immunization there were no grade 2 or 3 AEs and only 3/5 volunteers had self-limited grade 1 AEs, while 2/5 volunteers had no malaria-related AEs. NSAIDs used presumptively appear to completely eliminate the severe adverse events associated with PfSPZ-CVac (chloroquine) administered at doses of $2.0x10^5$ PfSPZ of PfSPZ Challenge.

Plans for this trial (CQ). In PfSPZ-CVac-Tü3 the same approach will be evaluated. Here, fully infectious PfSPZ Challenge (N54) will be used for immunization in adult, healthy, malaria-naïve volunteers. Injections will be given by DVI three times on Days 0, 5 and 28, using $1.1x10^5$ PfSPZ followed by repeated, heterologous CHMI with PfSPZ Challenge (7G8). Attenuation during immunization will be achieved through concurrent chemoprophylaxis with chloroquine (10mg/kg). We believe that by delaying the booster dose from the +10 days to +28 days after initiation of immunization (Sanaria, unpublished) will significantly increase vaccine efficacy (VE).

2.4. Drugs for PfSPZ-CVac approach

Only CQ, mefloquine, azithromycin, pyrimethamine and atovaquone/proguanil (A/P) have been used as drug partners for CPS or PfSPZ-CVac. [36-40] All showed acceptable tolerability and good safety, but azithromycin, pyrimethamine, and A/P may interfere with the acquisition of antiparasitic immunity (Mordmüller, unpublished data, Healy, unpublished). In fact poor protective efficacy has been associated with use of pyrimethamine and A/P as partner drugs in the PfSPZ-CVac approach, although, as described above, higher doses of PfSPZ Challenge (e.g., $2.0x10^5$ PfSPZ) administered with pyrimethamine as the partner drug appear to overcome this deficit, protecting 4/4 research subjects against heterologous CHMI at 12 weeks (ClinicalTrials.gov ID: NCT02511054, preliminary data). The high rates of AEs (especially neuropsychiatric adverse events) makes mefloquine a suboptimal candidate for large-scale use of the PfSPZ-CVac approach. Thus, only PfSPZ Challenge administered with CQ or with pyrimethamine for PfSPZ-CVac has shown high level protective efficacy after CHMI.

2.4.1. Drugs for PfSPZ-CVac approach – chloroquine

In the two published trials on immunization by CPS in humans CQ was used as the prophylactic agent[36,38]. The choice was based on experiments in rodents[39], which have been complemented recently by animal studies on alternative drugs that act before invasion of erythrocytes[41]. CQ use in CPS is rational because it has a well-studied safety profile, acts only against asexual blood-stages of the parasite, not against sporozoites or liver-stages, and NF54 blood-stage parasites are fully CQ-sensitive. Therefore, parasite multiplication during the clinically silent liver phase is not impeded and consequently the immune system is exposed to a maximum of antigen.

CQ was once the most important drug for the treatment and prophylaxis of all plasmodial species that infect humans and is still used in the treatment of some autoimmune diseases (e.g. discoid lupus erythematosus and rheumatoid arthritis). The rise in resistance of Pf against CQ, which

commenced almost immediately after market introduction but became most significant in the 1990's, led to a decrease in its importance for malaria treatment and prophylaxis. Since the beginning of this millennium, alternative antimalarials have been introduced on a large scale. Unlike many current clinical isolates from Africa, South America or South-East Asia, NF54 (the strain contained in PfSPZ Challenge (NF54)) is fully sensitive to CQ as well as all other registered antimalarial drugs.

In a proof-of-concept CPS trial[36], CQ was used over 14 weeks with once weekly administration of 300 mg chloroquine base (CQ), preceded by a loading dose of two doses of 300 mg CQ on two consecutive days one week earlier, which gives a total dose of 75 mg/kg CQ for a 60 kg person.

Although it was one of the most widely used antimalarials, CQ's toxicity and tolerability profile is not excellent. Especially, the small therapeutic range is problematic. Cardiac toxicity is a main problem following overdosage and several of the frequent adverse effects such as pruritus in Africans and gastrointestinal discomfort can lead to compliance problems, although pruritus has occurred only rarely in the two PfSPZ-CVac trials conducted in Africa, and in each case was self-limited and transient, despite continued administration of the drug (Sanaria, unpublished). Another disadvantage of CQ and mefloquine are their exclusive activity against asexual blood stage parasites. Immunization with PfSPZ induces pre-erythrocytic stage immunity and the level of protective efficacy depends on PfSPZ dose. Because CQ and mefloquine only act in the second half of the first asexual blood stage cycle following liver egress, malaria symptoms can occur. Previous trials have shown that adverse events due to parasitemia depend on PfSPZ dose. Hence the headroom for further dose escalation may be somewhat limited, although presumptive use of NSAIDs clearly increases the ceiling.

2.5. Microbial controlled infection studies of human volunteers

The deliberate infection of human volunteers with microorganisms has contributed uniquely to our understanding of the pathogenesis, immune responses and the treatment and prevention of numerous microbial diseases including influenza, cholera, typhoid fever and hepatitis[42]. A review by the UK Academy of Medical Sciences on microbial challenge studies recognized that such studies are important for providing proof of concept for therapeutic interventions and can significantly accelerate progress to Phase 3 studies[42]. CVac-Tü3 will use CHMI to assess the efficacy of immunization by the PfSPZ-CVac approach. Since PfSPZ Challenge (NF54) will be used for immunization and PfSPZ Challenge (7G8) for CHMI in this trial, background, associated risks, advantages and procedures to administer PfSPZ Challenge products will be described in the following sections.

2.6. Controlled human malaria infection (CHMI)

Pf is an infectious agent particularly well suited to CHMI studies. It has a relatively short asymptomatic incubation period, well-established quantitative diagnostic laboratory tests (thick blood smear microscopy and molecular assays), availability of highly efficacious chemotherapeutics, and no long-term sequelae or infectious state following appropriate and timely treatment. Studies using CHMI are a powerful tool for investigating malaria vaccines and prophylactic drug efficacy[43] and with an increasing number of candidate malaria vaccines being developed, the need for centers conducting CHMI trials is rising [43].

Deliberate infection of humans with malaria was first reported in 1917 by Julius Wagner-Jauregg, primarily as a therapy for patients with neurosyphilis[44]. At the time fever was thought to be beneficial for inhibiting the progress of neurosyphilis and malaria was used as a fever-inducer. 'Malaria treatment' became a standard intervention and thousands of patients were treated accordingly. Parasites were administered by the bites of infectious mosquitoes, by IV or subcutaneous inoculation of dissected salivary glands containing *Plasmodium* sporozoites suspended in media or of donor blood-stage parasites. The practice stopped with the advent of penicillin treatment for neurosyphilis.

In the 1950s and 1960s, CHMI trials were used to assess the effects of antimalarial medicines on healthy non-immune Australian army members[45,46] and male inmates in the United States[47]. Following the development of protocols for the continuous culture of Pf in 1976[48] and for the generation of mature Pf gametocytes *in vitro* in 1981[49], it became possible to produce laboratory-reared infectious mosquitoes[50], meaning that CHMI trials could be performed more routinely and standardized (e.g. using the same, well-characterized parasite isolate).

The first well-documented CHMI with laboratory-reared infectious mosquitoes was carried out in 1986 at the US Walter Reed Army Institute of Research (WRAIR), the US Naval Medical Research Institute (NMRI) and the US National Institutes of Health (NIH). Six volunteers were infected with PfSPZ by the bites of infectious *Anopheles freeborni* and *A. stephensi* mosquitoes [51]. The following year, the efficacy of the first recombinant protein and synthetic peptide Pf was tested in experimentally infected volunteers [52,53].

CHMI has now become established as a key tool to assess the efficacy of novel malaria vaccines and drugs[43]. As CHMI trials are carried out in a controlled environment, they allow unprecedented detailed evaluation of parasite growth and immunological responses, providing essential information for vaccine and drug development[43].

Since the late 1980s, the number of institutions carrying out CHMI with Pf has been growing. In 2007, data were published from a total of 532 volunteers[50]. Another report shows that a total of 1,343 volunteers were experimentally infected with Pf between 1985 and 2009[54].

2.6.1. CHMI – mosquito bites

The administration of PfSPZ by mosquito bite has been used as a method to test pre-erythrocytic stage vaccines for efficacy since the early 1970s [24,25]. Since the late 1980s, standardization of experimental sporozoite infections of humans (controlled human malaria infection – CHMI) has improved and efforts to further improve harmonization are ongoing. PfSPZ-infected mosquitoes for CHMI are currently produced at the Walter Reed Army Institute of Research, US Military Malaria Vaccine Program, USA; Radboud University Medical Centre (RUMC) in Nijmegen, the Netherlands; Seattle Biomed, USA; Sanaria Inc., USA; and (in the past) Johns Hopkins School of Public Health, USA[55]. All centers use *A. stephensi* mosquitoes that feed on either the CQ-sensitive NF54 strain of Pf or the 3D7 clone of NF54. In addition, limited numbers of volunteers have undergone CHMI with the CQ- and pyrimethamine-resistant 7G8 clone of Pf[26,56]produced at Sanaria Inc. and most recently with the multi-drug resistant NF135.C10 clone of Pf[57] produced at RUMC and Sanaria. CHMI studies are done at all of these centers, except Sanaria. Additionally, CHMI is frequently done with mosquitoes from these centers at the University of Maryland Center for Vaccine Development, USA and the University of Oxford, UK.

Fourteen to 21 days after feeding, mosquitoes are checked for infection by microscopic examination of salivary glands. Healthy human volunteers are subsequently exposed to the bites of five infectious mosquitoes for either 5 or 10-minute intervals until each volunteer has been bitten by 5 infectious mosquitoes. With this approach, virtually all volunteers develop patent parasitemia, although exceptions occur [50,58]. Infection rates drop significantly when volunteers are exposed to fewer than five infected mosquitoes[58,59].

A major strength of the sporozoite infection model is the use of infectious mosquitoes, mimicking the natural route of infection. However, mosquito bite CHMI is limited by a number of factors:

- It is impossible to control the number of sporozoites inoculated by biting mosquitoes. This number is generally thought to vary up to a maximum of several thousand sporozoites[43].
- The CHMI can only be performed in centers with access to an appropriate insectary and entomology staff, considerably limiting the number of sites that can perform these trials[43].
- If the dose of sporozoites needs to be controlled, mosquitoes need to be dissected after feeding and subsequent re-feeding may be necessary. Dosing based on the numbers of sporozoites inoculated is not possible.

- The requirement for having infected mosquitoes and screened volunteers available at the same time reduces the time-window that can be used to inoculate volunteers. This imposes difficulties on volunteer-sparing, group-sequential and adaptive study designs.

2.6.2. CHMI – PfSPZ Challenge (NF54 and 7G8)

In principle, the most accurate and practical way of dosing sporozoites is to inject them directly by needle and syringe [43]. This has the following advantages over the traditional mosquito bite CHMI:

- It allows CHMI trials to be conducted at multiple sites that currently do not have access to sporozoite-infected mosquitoes.
- The number of sporozoites with which volunteers are inoculated can be easily calculated and predefined.
- pThe potential impact of variation in infectivity of mosquito-administered sporozoites when performing parallel clinical trials at multiple sites or sequential clinical trials at the same site is eliminated. Consequently, comparability increases substantially.
- Practical advantages are provided, including ease of administration and the ability to inoculate volunteers over an extended period with different doses, routes, or concomitant medications rather than all on the same day. This enables the conduct of group-sequential and adaptive study designs, which in turn reduces the numbers of volunteers required to proceed in clinical development.

Sanaria Inc. is a biotechnology company that has developed aseptic, purified cryopreserved PfSPZ products for CHMI via injection (PfSPZ Challenge (NF54) and PfSPZ Challenge (7G8)). The salivary glands of aseptic *A. stephensi* mosquitoes infected with PfSPZ are removed by dissection and triturated to release the sporozoites. The sporozoites are purified, counted and cryopreserved at a specified concentration to produce the inoculum, which is called PfSPZ Challenge product. This process is in compliance with current Good Manufacturing Practices (cGMP) and regulatory requirements for production of a high-quality PfSPZ Challenge product suitable for parenteral injection.

The first CHMI trial using aseptic cryopreserved PfSPZ administered by needle injection was performed by RUMC between October 2010 and July 2011 (ClinicalTrials.gov Identifier: NCT01086917)[60]. It was a dose finding study to establish which dose of PfSPZ Challenge (NF54) administered intradermally (ID) would infect 100% of inoculated volunteers. Inoculation of PfSPZ Challenge (NF54) was well tolerated and safe. In all three groups (2,500, 10,000 or 25,000 PfSPZ ID), 5 out of 6 inoculated volunteers were successfully infected with Pf (infection rate: 83%). Hence infection rate was independent of PfSPZ Challenge (NF54) dose. Geometric mean and range of the pre-patent period was similar for all dose groups i.e. 13.0 (12.3; 14.0), 12.7 (11.0; 14.0), and 13.0 (12.3; 14.3) days for the groups receiving 2,500, 10,000, and 25,000 PfSPZ, respectively. Parasite densities at the time of diagnosis were similar among the groups (12,300, 11,200, and 23,400 parasites per mL in the three groups, respectively). All infected volunteers developed symptoms of Pf infection. Comparison with data from CHMI studies at the same site using mosquito bites showed that the pre-patent period was longer and parasite multiplication rates were similar when PfSPZ Challenge (NF54) was used[60,61].

It is important that any CHMI model infects all control volunteers. Why infectivity did not increase with increasing doses of PfSPZ in this study is unclear. At a meeting sponsored by the European Malaria Vaccine Development Association (EMVDA) in Amsterdam in June 2011, scientists from institutions undertaking CHMI studies around the world met to discuss optimal ways to increase infectivity with PfSPZ Challenge products.

As a consequence of this meeting, a second CHMI trial using aseptic cryopreserved PfSPZ administered by needle injection was performed in 2011 at the University of Oxford, UK[55]. This was an open label, human pilot study to optimize CHMI administered by PfSPZ Challenge (NF54) to compare ID with two different doses of intramuscular (IM) administration of PfSPZ Challenge (NF54). Inoculation of PfSPZ Challenge (NF54) by the IM route was well tolerated. Five of 6

volunteers in Group 1 (2,500 PfSPZ ID), 3 of 6 volunteers in Group 2 (2,500 PfSPZ IM), and 6 of 6 volunteers in Group 3 (25,000 PfSPZ IM) developed Pf parasitemia. However, the median pre-patent period of 12.7 days in the Group 3, the group that had 100% infection was considerably longer than the pre-patent period of 11 to 11.5 days generally seen with CHMI studies using mosquito bites for SPZ administration in the United States and Oxford.

In addition, trials of PfSPZ Challenge (NF54) were conducted in Tanzania (ID)[62], the United States (ID), Germany (IV)[63], Spain (IM and IV)[63,64], and Kenya (IM)[65] in 2012-2013:

Since 28 October 2010, 745 volunteers in 10 countries have undergone CHMI with PfSPZ Challenge.

1) Tanzania: At the Ifakara Health Institute (IHI) in Bagamoyo, Tanzania, in collaboration with the Swiss Tropical and Public Health Institute (Basel, Switzerland) 30 volunteers were inoculated ID with PfSPZ Challenge (NF54) at a dose of 10,000 (n=12), 25,000 (n=12), or saline (n=6) as control (ClinicalTrials.gov Identifier: NCT01540903)[62]. Overall the challenge by the ID route was well tolerated in all volunteers. There were no acute systemic allergic reactions or local adverse events following the injection. The occurrence of AEs was similar in all groups. Most AEs recorded following challenge were consistent with symptoms associated with clinical malaria. There were two unrelated SAEs in this trial. The first SAE involved a volunteer who was diagnosed as having gastroenteritis/dysentery of Grade 3 severity at the time of hospitalization, and the second SAE occurred in an individual who was admitted to the hospital and diagnosed with severe malaria based on disorientation on day +89 post injection of PfSPZ Challenge (NF54) (the volunteer was previously treated and successfully cleared of parasites on day +14 post CHMI). A genetic analysis of the infecting parasite showed that it was distinct from NF54, and thus must have been naturally acquired. Eleven of 12 and 10 of 11 subjects, who received 10,000 and 25,000 PfSPZ respectively, developed parasitemia. In 10,000 versus 25,000 PfSPZ groups geometric mean days from injection to Pf positivity by thick blood film was 15.4 versus 13.5 (P = 0.023). Alpha-thalassemia heterozygosity had no apparent effect on infectivity. PfSPZ Challenge (NF54) was safe, well tolerated, and infectious.
2) United States: At the University of Maryland 30 volunteers were randomized to one of three cohorts (ClinicalTrials.gov Identifier: NCT01546389). Each cohort consisted of two groups randomized to either 2 or 8 PfSPZ Challenge (NF54) ID injections of five volunteers per group with PfSPZ doses of 10,000 or 50,000 parasites in different volumes (Cohort 1: 10,000 PfSPZ given as 2 [group a] or 8 [group b] doses of 50 µL each; Cohort 2: 10,000 PfSPZ given as 2 [group c] or 8 [group d] doses of 10 µL each; 50,000 PfSPZ given as 2 [group e] or 8 [group f] doses of 10 µL each). Overall, the challenge by the ID route was well tolerated in all volunteers. Twenty-three (79%) of the 30 enrolled subjects developed Pf parasitemia. The range for time to parasitemia was 12 to 16 days. Five subjects (17%) did not develop malaria. Two subjects were treated before parasitemia could develop. One SAE was reported to the FDA by the Sponsor (Division of Microbiology and Infectious Diseases, National Institute of Allergy and Infectious Diseases, NIH). However, the principal investigators, the internal safety monitor, the Safety Monitoring Committee (SMC), and the investigational review board at the University of Maryland did not consider this event to be a SAE. Briefly, a volunteer, who developed Pf parasitemia and was successfully treated with A/P, was shown to have parasite DNA (the equivalent of approximately 290 parasites/mL) in his blood on Day 28 after CHMI, and 11 days after completing treatment with A/P. The volunteer was asymptomatic and the blood smear was negative on Day 28 after CHMI. Five days later, on Day 33 after CHMI, the volunteer was asymptomatic and blood smear negative but the PCR was positive at 31 parasite equivalents per mL of blood. On Day 35 the blood smear was negative and the volunteer was asymptomatic, but it was decided to treat the volunteer with an additional course of CQ. PCR was negative before treatment on Day 35. In total, 35 adverse events were reported. There were no events with a severity of Grade 3 (severe) and only one SAE (described above). There were 32 unsolicited adverse

events recorded that were deemed unrelated to the study challenge. Thirteen adverse events were reported during the seven days following injection of PfSPZ Challenge (NF54), Days 0–7. Twenty-two adverse events were reported during the malaria illness period, Days 8–14, at which time malaria was anticipated.

3) Germany: In June 2012, the first volunteer received cryopreserved PfSPZ by IV administration of PfSPZ Challenge (NF54) at the Institute of Tropical Medicine Tübingen, Germany (TÜCHMI-001; ClinicalTrials.gov Identifier: NCT01624961). In a sequential dose-escalation trial 50, 200, 800, and $3.2x10^3$ PfSPZ were administered IV via an indwelling catheter until a dose that infected 9 out of 9 volunteers was established and, as a secondary endpoint, the geometric mean pre-patent period was 12 or less days. The identified dose that consistently infects all volunteers serves as a reference for dosing of other PfSPZ products and comparison with mosquito bite mediated infection since IV administration is the most direct way of inoculation. A control group received 2,500 PfSPZ by intradermal injection to assess infectivity of the PfSPZ Challenge (NF54) lot and to compare parasitological and clinical outcomes between sites. Four out of six volunteers became slide positive after ID injection of 2,500 PfSPZ between 12.3 and 15.3 days after injection (geometric mean: 13.6) and 9 out of 9 volunteers turned positive following injection of $3.2x10^3$ PfSPZ IV with a geometric mean pre-patent period of 11.2 days (Table 1 and Figure 2), which is comparable to the pre-patent period achieved with mosquito bites. PfSPZ Challenge (NF54) IV was well tolerated in all 24 volunteers at all doses and no SAE occurred. Volunteers with parasitemia developed mild or moderate symptoms of malaria and were cured with standard antimalarial treatment. Follow-up of the volunteers has been completed and no SAEs occurred. Subsequently, another 6 volunteers received $3.2x10^3$ PfSPZ Challenge (NF54) by IV injection at the Barcelona Centre for International Health (BACHMI-01) (CRESIB; ClinicalTrials.gov Identifier: NCT01771848) – see below for a full description of this study. The experiment was done to assess if $3.2x10^3$ PfSPZ can reproducibly infect volunteers at a different site with different investigators and setting. In addition, PfSPZ were administered by direct injection in the vein instead of through an intravenous catheter (direct venous inoculation – DVI). All 6 volunteers became infected with a geometric-mean pre-patent period of 11.4 (range 10.4–12.3) days. Hence, IV injection of PfSPZ Challenge products is the preferred way of infecting human volunteers reproducibly and in a most standardized way.

Table 1: TÜCHMI-001 and BACHMI-01 (IV) infection rate and pre-patent periods

Group	Inoculated (N)	Parasitemic (N)	Prepatent period in days* (days)	Incubation period‡ (days)
ID 2500	6	4	13.6 (12.3 – 15.3)	14.1 (13.0 – 16.0)
IV 50	3	1	13.3 (NA)	7.5 (NA)
IV 200	3	1	13.9 (NA)	15.0 (NA)
IV 800	9	7	11.7 (10.9 – 12.5)	11.8 (11.0 – 12.5)
IV 3200	9	9	11.2 (10.5 – 12.5)	9.4 (7.0 – 12.5)
DVI 3200	6	6	11.4 (10.4 – 12.3)	10.6 (10.0 – 12.0)

* Time from inoculation to first positive thick blood smear, given as geometric mean (min–max)
‡ Time from inoculation to first symptom of malaria, given as geometric mean (min–max)
NA: not applicable (n = 1), ID: intradermal, IV: intravenous, DVI: direct venous inoculation (verification group)

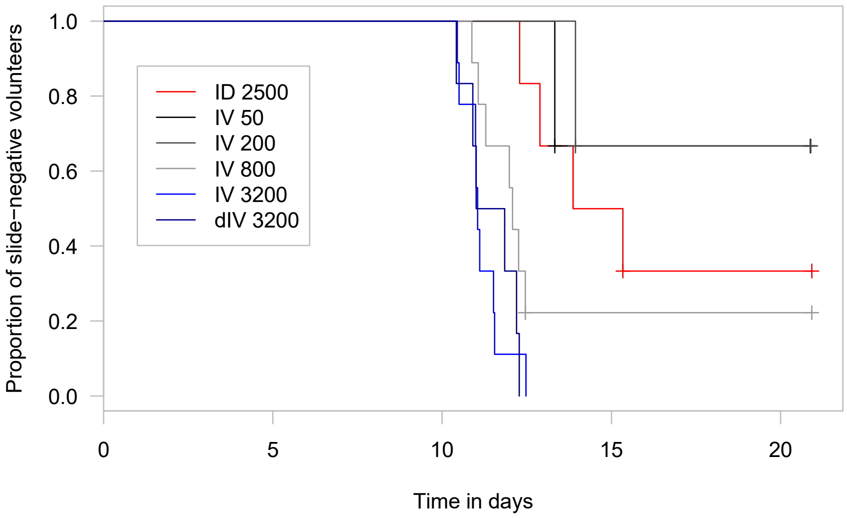

Figure 2: Kaplan Meier Plot for time to parasitemia. dIV: direct venous inoculation

TÜCHMI-002 (ClinicalTrials.gov ID NCT02115516) is a dose-escalation and regimen optimization trial to identify the best regimen for infection under chemoprophylaxis using PfSPZ Challenge (NF54) and chloroquine; the PfSPZ-CVac approach. A total of 67 volunteers received at least one immunization with 3.2×10^3 (n = 9), 12,800 (n = 9) or 51,200 (n = 28) PfSPZ Challenge (NF54). In general, all immunizations were well tolerated and no serious adverse events occurred so far. In Stage A, 100% protection was achieved with 51,200 PfSPZ administered at four-week intervals. Two week and five-day intervals led to 67% (6/9) and 63% (5/8) protective efficacy, respectively. In addition, it was shown that 2 g azithromycin given as an extended release formulation (Zithromax Uno) on the day of PfSPZ inoculation does not prevent parasite egress from the liver and is therefore not a suitable alternative to CQ chemoprophylaxis.

4) Spain: As described above, a PfSPZ Challenge (NF54) study was conducted in Barcelona, Spain. The study had two parts. In the first part (Part A) 18 volunteers were inoculated with PfSPZ Challenge (NF54) by intramuscular injection. Volunteers were divided into three groups of 6 volunteers. All groups received the same dose of 2,500 PfSPZ but administered in different volumes: 10 µL, 50 µL and 250 µL. Thus, in Part A of the study, Group 1 received 2,500 PfSPZ in 10 µL, Group 2 received 2,500 PfSPZ in 50 µL, Group 3 received 2,500 PfSPZ in 250 µL, all in two intramuscular injections, one in each arm into the deltoid muscle. Once Part A data were available, a preliminary analysis was performed in order to determine the optimal volume of inoculation. Thereafter, the second part of the study was initiated (Part B) with 18 new volunteers included and inoculated by IM or IV injection. The objective of Part B was to assess the effect of the optimal volume of inoculation determined in Part A when administered with different PfSPZ doses (25,000 PfSPZ and 75,000 PfSPZ) by IM administration, compared to the IV administration of 3.2×10^3 PfSPZ (regimen that achieved a 100% infective rate in the CHMI carried out at the University of Tübingen, Germany, with a geometric mean pre-patent period of 11.2 days; see above). Thus, in Part B of the study, Group 4 received 3.2×10^3 PfSPZ in 500 µL by intravenous injection in an arm vein. All (6/6) became parasitaemic with a geometric mean of 11.4 days. Group 5 received 25,000 PfSPZ in 10 µL and Group 6 received 75,000 PfSPZ in 10 µL in two intramuscular injections, one in each arm over the deltoid muscle. There were no acute systemic allergic reactions. Only one volunteer presented a local adverse event following the injection and no SAEs occurred. Most AEs recorded following challenge were consistent with symptoms associated with clinical malaria.

5) Kenya: An open label non-randomized controlled human malaria infection (CHMI) pilot study in 28 healthy volunteers with varying exposure to malaria was carried out in Kenya (Pan African Clinical Trials Registry No. PACTR201211000433272) [65].The trial aimed to assess parasite growth and its relationship with functional immunity to Pf. CHMI was carried out with inoculation of PfSPZ Challenge (NF54) intramuscularly (IM) by needle and syringe as outlined in Table 2 below.

Table 2: Study Design Kenya

Group No	No. Volunteers	Prior exposure to malaria	Sporozoite doses	Total volume administered per Injection site	No injection sites
1	2	Nil	25,000	50 µL	2
2	2	Extensive	25,000	50 µL	2
3	2	Nil	75,000	50 µL	2
4	2	Extensive	75,000	50 µL	2
5	2	Nil	125,000	50 µL	2
6	2	Extensive	125,000	50 µL	2
7	8	Nil	125,000	50 µL	2
8	8	Extensive	125,000	50 µL	2

All volunteers were successfully infected and symptomatic, with the exception of one volunteer who was asymptomatic, despite positive qPCR confirming blood stage infection. No unexpected or serious AEs occurred.

6) Gabon, Kenya and The Gambia (CHMI to study immunity to malaria): In 2014, at the Centre de Recherches Médicales in Lambaréné, Gabon, 25 healthy, adult volunteers were inoculated with 3.2×10^3 PfSPZ Challenge (NF54) by DVI (LACHMI-001; ClinicalTrials.gov ID: NCT02237586). Five volunteers were malaria-naïve and 20 had lifelong heavy exposure to malaria. Of those, 9 were heterozygous for the sickle cell gene mutation (HbAS) and 11 were wildtype (HbAA). No unexpected or serious AE occurred and CHMI was safe in all volunteers. Mild and moderate malaria symptoms occurred in all malaria-naïve volunteers, but were much reduced in malaria-exposed volunteers. Four of the malaria-exposed volunteers (two with HbAA and two with HbAS) did not develop malaria or parasitemia by thick blood smear or qPCR[66].

In 2016, the KEMRI-Wellcome Trust Unit in Kilifi initiated a similar study of naturally acquired immunity to malaria (ClinicalTrials.gov ID: NCT02739763). This study was restricted to volunteers with AA hemoglobin. So far, PfSPZ Challenge (NF54) has been administered to more than 100 malaria-exposed Kenya adults in this study, to evaluate the mechanisms underlying naturally acquired immunity. The study has documented similarly favorable safety and tolerability profiles. As in Gabon, not all volunteers developed parasitemia by TBS or PCR, interpreted as reflecting anti-parasite immunity developed through natural exposure.

Most recently, a similar study of about 20 research subjects was conducted at the Medical Research Unit in Banjul, The Gambia.

7) Vaccine and Drug Efficacy Trials: As of January 1, 2018, PfSPZ Challenge (NF54) has been used at the established infective dose (3.2×10^3 PfSPZ administered by DVI) to test the efficacy of PfSPZ-CVac or PfSPZ Vaccine in Tübingen, Seattle, and at NIH in 193 malaria-naïve adults, and Tanzania, Mali, Equatorial Guinea, and Gabon to test the efficacy of PfSPZ-CVac, PfSPZ Vaccine, pr GMZ2 vaccine in 199 malaria-exposed adults [34,40]. In these studies, all malaria-naïve, non-immunized volunteers have developed

parasitemia, but not all recipients in Africa have developed parasitemia[66]. Similar studies have been done to assess the efficacy of the antimalarial drug DSM265 in Tübingen and Seattle in 37 malaria naïve subjects[67] . Finally, 5 volunteers were injected with PfSPZ Challenge (NF54) at the University of Maryland to serve as NF54 controls for a study of CHMI induced by DVI of PfSPZ Challenge (7G8). These trials using PfSPZ Challenge to assess efficacy have included 434 study subjects, and CHMI has been safe and well tolerated in each case.

2.6.3. Characterization of PfSPZ Challenge (7G8)

In CVac-Tü3, a heterologous strain will be used for repeat CHMI of volunteers immunized with three doses of 100,00 PfSPZ. PfSPZ Challenge (7G8) is manufactured and characterized by essentially the same methods as is PfSPZ Challenge (NF54) except that it is comprised of PfSPZ from the 7G8 clone of Pf. The 7G8 clone of Pf (Pf7G8) was derived from a Brazilian isolate, IMTM22, obtained from a 12-year old male near Manaus, Brazil on 12 March 1980. The isolate was cryopreserved and adapted to continuous culture. After 19 weeks of continuous culture, limiting dilution was used to produce clones. The clone 7G8 was selected for ability to produce microgametes, exflagellate, and infect *A. freeborni* resulting in oocysts and sporozoites. It was tested for drug sensitivity by the Division of Experimental Therapeutics, Walter Reed Army Institute of Research (WRAIR) and was found to be resistant to CQ, and sensitive to mefloquine[68]. Additional testing by the University of South Florida confirms resistance to chloroquine and sensitivity to mefloquine, artemether-lumefantrine and A/P. All infections after CHMI with the 7G8 clone have been treated successfully with mefloquine or with atovaquone/proguanil[33,69] (Mordmüller unpublished, Epstein and Lyke unpublished, Laurens unpublished).

Pf 7G8 has a different genomic sequence than does Pf NF54 and a distinct microsatellite map[70,71]. Using PCR-based typing with primers flanking a polymorphic microsatellite (PfRRM) within a known multicopy PfRR, or rif, repetitive element of Pf, parasite isolates can be distinguished[70,71,72]. Thus, if Pf 7G8 will be used to assess protective immunity by CHMI in subjects immunized with the PfSPZ-CVac approach (using PfSPZ Challenge (NF54), it will assess the capacity of PfSPZ-CVac to protect against heterologous parasites.

PfSPZ Challenge (7G8) has been assessed for infectivity as part of another trial (MAVACHE, NCT02704533) performed at the center in Tübingen. Here, volunteers received 800 (n = 3), 1,600 (n = 3) or $3.2x10^3$ (n = 3) PfSPZ Challenge (7G8) by DVI. In the two low-dose groups 2/3 volunteers developed malaria, whereas $3.2x10^3$ PfSPZ induced malaria in all three volunteers with similar kinetics as PfSPZ Challenge (NF54) and CHMI by mosquito bite. CHMI with PfSPZ Challenge (7G8) was well-tolerated and responded to treatment with A/P. PfSPZ Challenge (7G8) has also been studied at the University of Maryland Center for Vaccine Development, at doses of $0.8x10^3$, $1.6x10^3$, $3.2x10^3$and $4.8x10^3$ PfSPZ and infected 3/7 (43%), 4/7 (57%), 8/9 (89%) and 2/2 (100%) volunteers, respectivel (Laurens unpublished).

2.6.4. Conduct of CHMI trials

Following a collaborative consensus process involving investigators from the USMMVP, Sanaria Inc., University of Maryland, University of Oxford, RUMC, The Seattle Biomedical Research Institute and the KEMRI-Wellcome Kilifi Research Programme, a consensus document; 'Standardization of Design and Conduct of *P. falciparum* Sporozoite Challenge Trials' was developed, and provides a comprehensive guide to the appropriate conduct of malaria challenge studies[68,73]. Although there remain minor differences between centers in follow-up procedures in CHMI trial conduct, there is consensus on the following key points:

- All volunteers should have a medical assessment no longer than 48 hours before CHMI, including an interim medical history, directed physical examination, and pregnancy test for female volunteers.
- Follow-up visits should be scheduled at least once daily. At all visits, volunteers should be questioned about the occurrence of AEs and use of medication.

- In the event that a volunteer does not attend for a scheduled follow-up visit it is imperative that investigators find the volunteer as quickly as possible and assess for patent parasitemia and malaria. Should the volunteer withdraw consent from further follow-up prior to receipt of antimalarial drugs, it may be appropriate to administer a course of antimalarial chemotherapy under close supervision.
- Grading and reporting of AEs should be performed using international and local guidelines. It should be noted that the occurrence of a low frequency of Grade 3 AEs, of short duration and with no long-term sequelae, is not unexpected in clinical CHMI. A minority of those undergoing CHMI are known to experience Grade 3 systemic AEs and this fact should be included in the informed consent form.
- Vital signs should be recorded at least once daily and at any subsequent visits for medical attention. Directed physical examination should be performed when necessary.
- It is critical that every volunteer must receive every dose of antimalarial therapy. In some settings, fully directly observed treatment will be essential. Where directly observed treatment is not used, investigators must follow volunteers closely to ensure compliance with the treatment regimen and resolution of parasitemia.
- After CHMI, all volunteers should be followed until they have completely finished antimalarial treatment.
- Volunteers should be evaluated at least two weeks after finishing treatment.
- A local safety monitor and an independent SMC should be established to act as independent experts in evaluating AEs. The safety monitor or monitoring committee may advise the investigators on initiating antimalarial treatment for a specific volunteer or volunteer group. While SMCs are not a requirement for Phase 1 trials, they should be considered a requirement for CHMI trials which have an efficacy component and which have major potential safety concerns.

2.6.5. Clinical presentation post CHMI

Nearly all malaria-naïve volunteers in CHMI studies develop symptoms of malaria; approximately one-fifth of volunteers temporarily develop symptoms graded severe (symptoms that prevent daily activities) but serious or life-threatening malaria has never occurred[50]. The most common symptoms are fatigue and headache, and severe symptoms can include fever, headache, fatigue, malaise, chills, myalgia, rigors, nausea and vomiting. Clinical symptoms generally coincide with the detection by microscopy of thick film blood smears of blood-stage parasites at densities of 10 to 20 parasites per μL of blood [50]. This corresponds to a parasitemia of approximately 0.0004% infected erythrocytes [43]. Severe malaria is extremely unlikely unless parasitemia is at least 1000-fold higher than the peak parasitemia in CHMI trials and such parasitemia is allowed to progress without treatment. After the start of antimalarial treatment, symptoms can temporarily increase in severity but subside quickly within approximately 2 to 3 days [43]. Routine laboratory checks generally show a moderate decrease in leucocyte and platelet numbers during infection, with no change in hemoglobin concentration [58]. Bleeding or thrombogenic complications have never been described[50,58]. Abnormalities of liver enzymes have been observed, but these abnormalities did not result in clinical manifestations and they resolved after a few days to weeks[50,58].

Immediate treatment of volunteers at the earliest phase of microscopically detectable blood-stage infection ensures that the potential risks of complications associated with severe malaria are minimized to the greatest extent possible. Indeed, CHMIs have been shown to be safe in all volunteers treated since 1985 when the first volunteers were infected by exposure to mosquitoes that had fed on cultures containing Pf gametocytes [50,54,58,68,74]

Several years ago, safety concerns were raised because of a cardiac event in a young volunteer who underwent CHMI by mosquito bite at RUMC in the Netherlands shortly after initiation of antimalarial treatment for Pf infection. The adverse event resolved following treatment and did not re-occur in the one-year follow-up period. A relationship between the cardiac event and the experimental infection or its treatment was not established[75]. In addition, there was a cardiac related

SAE in an ongoing phase I trial at RUMC where PfSPZ Challenge (NF54) has been administered to volunteers in combination with the antimalarial drug, chloroquine (TIP5, ClinicalTrials.gov ID: NCT01728701, see above). Here, one subject experienced an episode of myocarditis (considered an SAE) following treatment for malaria. Specifically, the SAE occurred in Volunteer 1641-27, who underwent CHMI on Day 124 using five Pf NF54-infected mosquitoes. The CHMI occurred 60 days after he had received the last injection of PfSPZ Challenge (NF54) for immunization. On Day 9 post-CHMI the volunteer had a sore throat. On Day 11 post-CHMI (16th February 2013), the volunteer's thick film was positive for malaria, and treatment with A/P was initiated. The subject was asymptomatic, but due to elevated Troponin T levels (maximum: 299 ng/L), which are routinely assessed at RUMC on a daily basis, the volunteer was hospitalized on 18th February 2013 (Day 13 after CHMI). On 19th February 2013 the volunteer experienced chest pain for 10 minutes and received sublingual nitroglycerin spray. He was diagnosed with myocarditis based on a magnetic resonance imaging (MRI) and minor repolarization disturbances on electrocardiogram. The volunteer had no further symptoms on follow-up, and the Troponin T levels returned to normal by 5th March 2013. The volunteer was discharged on 22nd February 2013, and the electrocardiogram was normal on 8th March 2013. A follow-up cardiac MRI (8th July 2013), performed approximately 5 months after the start of the SAE, demonstrated good left ventricular function with mild hypokinesia in a few segments and some remaining mid-wall delayed enhancement. An etiology was not established, but the volunteer's throat swab was positive for rhinovirus on 19th February 2013. Furthermore, 14 days after the third immunization with PfSPZ Challenge (NF54), the volunteer received immunizations against six pathogens in preparation for travel. Nevertheless, it has been generally agreed that volunteers with an increased risk of cardiac disease should be excluded from such trials [70]. The spectrum of adverse events induced CHMI with by PfSPZ Challenge products is very similar to CHMI using infectious mosquitoes or injection of blood-stage parasites.

2.6.6. Heterologous CHMI with PfSPZ Challenge (7G8)

CVac-Tü3 will be the fourth study (after MAVACHE, the aforementioned study performed at the University of Maryland, and the study perfomed at NIH clinical center referenced in section 2.3.4), where PfSPZ Challenge (7G8) will be used to infect humans with malaria using the DVI route. We assume a similar phenotype for the two strains based on the fact that they have shown nearly identical characteristics when administered by mosquito bite and DVI. This includes consistent infection following 5 mosquito bites or $3.2x10^3$ PfSPZ by DVI, similar pre-patent periods, and a similar spectrum of malaria-related clinical symptoms associated with parasitemia. In the case of strain NF54, the dose of $3.2x10^3$ PfSPZ has been shown to duplicate the infectivity (~100%) and pre-patent period (GM of 10.5 to 11.5 days) seen when CHMI is conducted by the bite of 5 PfSPZ (NF54)-infected mosquitoes. The two clinical trials in the literature in which volunteers (n = 17) were infected by the bite of 5 PfSPZ (7G8)-infected mosquitoes, the infectivity rate was also 100% and the pre-patent was 9-12 days [56,76]. We have recently confirmed these findings: in a just-completed clinical trial of PfSPZ Vaccine (protocol WRAIR 2080) where four non-immunized control volunteers were infected by the bite of 5 (n = 3) or 4 (n = 1) PfSPZ (7G8)-infected mosquitoes produced by Sanaria Inc. (under a Biologics Master File filed with US FDA). The geometric mean pre-patent period was 11.7 days and the geometric mean parasite density at time of thick smear diagnosis was 3.4 parasites/µL blood. There were no Grade 3 or 4 adverse events (AEs) in these volunteers. The clinical manifestations of malaria were no different than those found in subjects who underwent CHMI by exposure to the bite of 5 PfSPZ (3D7)-infected mosquitoes in the same clinical trial (WRAIR 2080, ClinicalTrials.gov ID: NCT02215707) and who underwent CHMI in the same timeframe (3D7 is a clone of NF54); geometric mean pre-patent period was 11.9 days and GM parasite density at time of thick blood smear diagnosis was 4.0 parasites/µL blood.

2.6.7. Ethical considerations of CHMI trials

For any clinical trial, the risk for potential volunteers should be compared with the benefit. As no direct benefit accrues to CHMI trial participants, indirect benefits are considered in the context of possible public health gains that may occur as a result of scientific advances made through CHMI. This places a burden on the CHMI trial investigators both to exercise all possible safeguards for volunteer safety (primary consideration) and to ensure that maximal scientific benefit accrues from each CHMI trial (secondary consideration). Key ethical considerations agreed by consensus of the field include[73];

- Safety is the paramount consideration in conduct of CHMI trials. When CHMI trials are conducted at existing and new centers, volunteer safety is the main focus of practical considerations.
- Investigators are required to follow both international and local guidelines with respect to ethical considerations and in accordance with the Declaration of Helsinki and should fulfill all local regulatory and ethics committee requirements.
- CHMI trials should be conducted according to Good Clinical Practice Guidelines (either ICH or WHO). The scientific benefit should be maximized whilst minimizing risk, discomfort or distress to individuals. From this perspective, it is important that the results of CHMI trials enable comparative evaluation and collection of as much information as is reasonably possible, both in terms of the load of merozoites that emerges from the liver ('the liver-to-blood inoculum') and the blood-stage parasite growth rate prior to initiation of drug treatment as well as the proportion of volunteers completely protected. The availability of data to the scientific community also attains an ethical dimension in this perspective, with importance attached to access to data that may inform design of future CHMI trials and design of malaria vaccines.
- The raw data (both microscopy and PCR where available) from CHMI trial datasets should be made publicly available to facilitate scientific benefit to the community.
- If an unexpected SAE, which is possibly related to CHMI occurs at a CHMI trial center, recognizing legal restrictions, every effort should be made to communicate information on this SAE to the community of CHMI trial centers within 90 days of the occurrence of the SAE. This is in addition to the usual reporting requirements to ethical committees, sponsors and regulatory authorities. SAEs that are unambiguously not related to the challenge procedure are excluded, e.g. hospitalizations for clearly coincidental events such as trauma. Where there is any doubt community-wide notification should occur. This is because safety of participants at other centers may be affected by occurrence of an SAE at one center.

2.6.8. PfSPZ-CVac using PfSPZ Challenge (NF54) as the immunogen and CQ as the partner drug for chemoprophylaxis of PfSPZ

There have been six clinical trials of PfSPZ-CVac in malaria naïve, healthy males in the U.S. and Europe. Five of the six trials have at least one group (arm) in which CQ was the only drug for chemoprophylaxis. These clinical trials are listed chronologically by first injection date in Table 3, and the results of vaccine efficacy are also shown. In total 141 malaria-naïve adults have been immunized with PfSPZ-CVac in these trials, and the majority of volunteers have received CQ as the partner drug. The results of the first two trials outlined in Table 3 have been published [40,77].

2.6.9. Adverse events in malaria naïve adults

Most AEs following PfSPZ-CVac immunization in malaria-naïve individuals have been mild or moderate in severity. No grade 3 AEs were recorded days 7-9 after PfSPZ Challenge administration in the TÜCHMI-002 study or the CQ-only group of the 15-I-0169 study. Both studies involving the administration of 5.12×10^4 PfSPZ of PfSPZ Challenge as the maximum dose. Detailed data are available in the Investigator's Brochure (edition 7.0, 21 November 2017) for PfSPZ-CVac.

Surprisingly, several grade 3 AEs were recorded in the follow-on 11-0042 trial in Seattle, also using CQ as the partner drug and the same dose of PfSPZ Challenge. These occurred in 4/9 volunteers, and included grade 3 fatigue, malaise, headache, chills, myalgia and arthralgia. Six volunteers had temperatures of 100.0 degrees F or higher, the highest 102.3°F. The number of grade 3 adverse events in the 11-0042 trial was statistically significantly higher when compared to the TÜCHMI-002 and 15-I-0169 trials, which used the same dose of PfSPZ, where none occurred. One hypothesis to explain this difference is that the increased severity in cohort 1 of the 11-0042 trial related to the 7-day interval used between immunizations in that trial. This interval superimposed administration of PfSPZ Challenge on the transient parasitemia resulting from the prior PfSPZ Challenge administration, potentially altering cytokine profiles and affecting reactogenicity. Because of the statistically significant increase in grade 3 adverse events in the 11-0042 trial, the 7-day interval will not be used in PfSPZ-CVac studies again in the future. The 5-day interval used in the Part B of the TÜCHMI-002 trial might appear to be similar to the 7 day interval used in the 11-0042 trial, but in marked contrast, the 5 day interval avoids the superimposition of newly injected PfSPZ on parasitemia caused by earlier injections, as shown in Figure 3. The fact that 0/8 volunteers were protected against homologous CHMI in Cohort 1 in the Seattle trial (7-day intervals) while 6/8 were protected against homologous CHMI in Cohort 3 in Seattle (5-day intervals) indicates that the superimposition of a new PfSPZ Challenge on the parasitemia induced by a prior immunization led to qualitatively different results than a 5-day, 14-day or 28-day interval, each of which avoids administration on top of existing parasitemia.

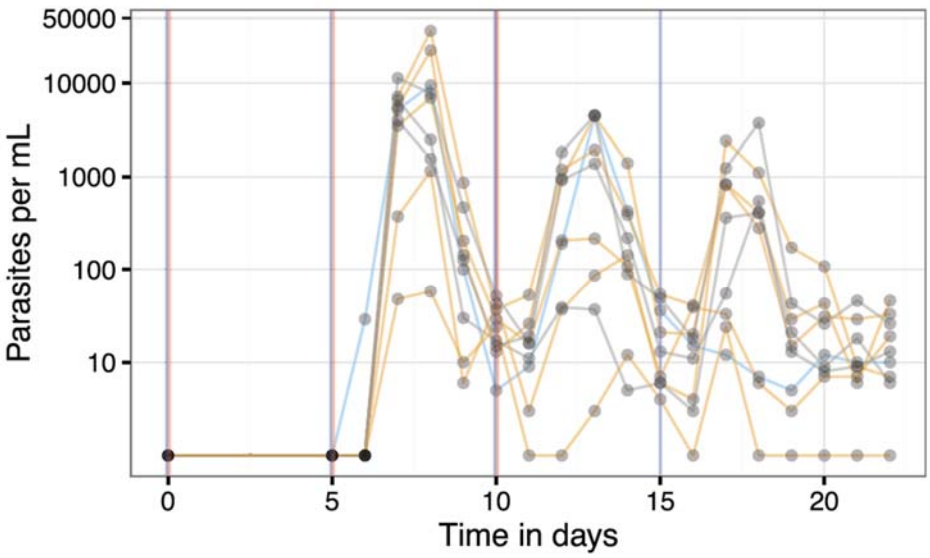

Figure 3: **Parasitemia Curves in the TÜCHMI-002 Trial using a Condensed (every 5 day) Regimen.** Vertical red lines: DVI of 5.12×10^4 PfSPZ of PfSPZ Challenge; vertical blue lines: CQ administration. Note that rising and peak parasitemias are avoided when immunizing using 5-day intervals (Mordmüller, 2017).

Due to the Grade 3 AEs in Cohort 1, a pilot group (Cohort 2a) was immunized next in Seattle, receiving 1.024×10^5 PfSPZ of PfSPZ Challenge at 7-day intervals. Although they were expected based on the results of Cohort 1, no grade 3 AEs occurred in these three subjects. When the poor results of protective efficacy (0/8) after CHMI of Cohort 1 became known, it was decided not to conduct CHMI for Cohort 2a. Discussions and review of data led to the hypothesis that there was a clear difference between 5 and 7 day intervals, based primarily on the dramatic difference in protection, but also considering the differences in reactogencity. Based on this discussion, and in consultation with the DSMB, it was decided that Cohort 3 would receive the same dose as Cohort 2a, 1.024×10^5 PfSPZ. but at 5 day intervals. Cohort 3 was then immunized. Although 1 volunteer experienced one grade 3 AE, a fever of 103°F, and was withdrawn from the study prior to the third immunization, overall the immunizations appeared better tolerated in the remaining 8 volunteers,

all of whom completed immunizations and underwent CHMI. 6/8 (75%) were protected, supporting the hypothesis that superimposition of a PfSPZ injection during parasitemia was deleterious to the induction of protective immunity. This marked the completion of the Seattle study.

Grade 3 AEs were subsequently recorded during transient parasitemia in the next study of PfSPZ-CVac in malaria-naive volunteers, trial 17-I-0067. Four volunteers in the pilot study were administered 1 dose of $2x10^5$ PfSPZ (a 4-fold higher dose of PfSPZ than just discussed for the TÜCHMI-002 trial (Parts A and B), the 15-I-0169 trial (CQ only group), and Cohort 1 of the Seattle trial, and a 2-fold higher dose of PfSPZ than just discussed for the Cohorts 2a and 3 in the Seattle trial), and 2 of 4 experienced transient grade 3 signs or symptoms. These grade 3 adverse events resolved in less than 12 hours, and the participants were able to promptly resume the activities of daily living. Three of 4 volunteers had a positive thick blood smear. These AEs presumably reflected the higher dose of PfSPZ (parasite densities as measured by PCR not yet available). As in all prior studies, the parasitemia appeared to clear quickly and no additional treatment was needed.

After completing the pilot study, the data were reviewed by the DSMB, which gave the team a green light to proceed to the main 17-I-0067 trial, which began in January, 2018. Five more volunteers were administered $2x10^5$ PfSPZ of PfSPZ Challenge. Based on the occurrence of the grade 3 AEs in the pilot group, the investigators instituted presumptive treatment with ibuprofen (400 mg q 6-8 hours) or naproxen (500 mg q 12 hours) starting the morning of day 7. This successfully limited to grade 1 or prevented altogether symptoms associated with parasitemia in the 5 immunized volunteers. Second and third doses of $2x10^5$ PfSPZ will be administered at 4-week intervals. Within a few months, it should be possible to tabulate the data from this trial and provide a more comprehensive report on the success of presumptive treatment for preventing grade 2 and grade 3 AEs, and also to report protection results.

No SAEs deemed related to PfSPZ Challenge have occurred in PfSPZ-CVac trials. However, two noteworthy AEs have occurred, the second of which was deemed possibly related to CQ administration (not to PfSPZ Challenge). The first noteworthy AE occurred in the TIP5 trial where PfSPZ Challenge was administered by intradermal injection. Several hours after the fourth CQ dose, one participant experienced transitory urticaria at multiple sites of the body lasting for 3 days (corresponding to days 5–8 after PfSPZ Challenge injection). The subject did not receive any treatment for the urticaria, continued in the study, received two more immunizations with PfSPZ-CVac (PfSPZ Challenge + CQ) and underwent CHMI, but did not develop recurrent urticaria or any other indication of an allergic reaction. The etiology of the urticaria is unclear. The second case was an SAE that occurred 3 days after immunization #3 in the 15-I-0169 trial. A 23-year-old participant in a group receiving both pyrimethamine and CQ presented with the acute onset of nausea, vomiting, headache, tinnitus, mental "fogginess" and confusion worsening over the course of a week. The participant was hospitalized for 4 days to facilitate evaluation and management, and treated with IV acyclovir for presumed herpes simplex virus encephalitis. Cerebrospinal fluid was not obtained, because the volunteer had a Chiari malformation noted on brain MRI scan. Symptoms improved over time with the main symptom of "fogginess" fully resolving in 11 days. Convalescent viral titers were unrevealing. Final diagnosis was encephalopathy of unknown etiology deemed possibly related to CQ, which has been reported to cause a similar clinical picture.

2.6.10. Case Report (TÜCHMI-002; volunteer 083)

The one exception to successful treatment by CQ occurred in the second part of the TÜCHMI-002 trial, during follow-up after the first immunization. The Group B1 volunteer received CQ as the partner drug. The research subject experienced parasitemia documented by PCR on day 7 after PfSPZ Challenge injection, with the parasite burden peaking on day 8 at a density by qPCR (15.7 parasites/uL) that was typical for the study. Parasitemia then fell progressively on days 9 and 10, consistent with natural cycling of blood stage parasite growth and/or partial killing by CQ. However, there was increasing parasitemia on day 11 by qPCR (Figure 4), and the subject was

found to be thick blood smear positive on day 13, and experienced mild symptoms of malaria (headache, sweating), suggesting that sufficient CQ was either not ingested or not absorbed adequately. Parasitemia peaked on day 13 (1271.2 parasites/uL). The volunteer was treated with atovaquone/proguanil on days 13 to 15 and parasitemia cleared promptly. A retrospective review of the day 11 thick blood smear (density of 92.8 parasites/uL by PCR) with more intensive searching revealed trace parasites.

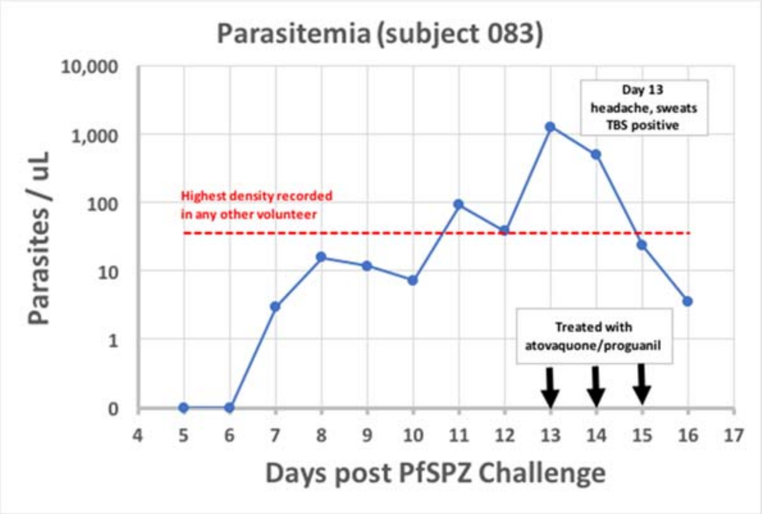

Figure 4: Parasitemia Curve for Volunteer 083

CQ was detectable in this participant's plasma on day 13, but was less than 2 ng/mL (limit of assay quantification 5 ng/mL). On questioning, the volunteer stated that he did swallow the CQ tablets. Notably, oral inspections post CQ ingestion were not performed in this trial. The volunteer initially agreed but later refused to undergo a pharmacokinetic study of CQ absorption and metabolism to rule out poor bioavailability as the cause of the very low CQ levels. However, since bioavailability of CQ is generally excellent (75-100%), poor bioavailability in this healthy volunteer seems unlikely as the cause. The most likely explanation is surreptitious avoidance of CQ ingestion – hiding the tablets in the cheek and spitting them out later, for example, or self-induced emesis after swallowing. Brief exposure of the oral or gastric mucosa to the drug in this fashion and subsequent absorption of small amounts would explain the trace levels measured in the blood and the initial drop in parasitemia on days 9 and 10. The parasite from this volunteer was isolated on day 13 and tested in vitro for CQ susceptibility, and was found to be highly sensitive, consistent with the testing that has been performed on Sanaria's master and working cell banks for PfSPZ Challenge. Since this event, Sanaria has recommended an oral examination after every CQ administration, to make sure the tablets have been swallowed. This can be combined with a period of observation after administration, to reduce opportunities for self-induced emesis. This case was reported to the US FDA.

In summary six clinical trials of PfSPZ-CVac have been completed (Table.3) or are underway (Table 4). PfSPZ-CVac (CQ) has shown 75%-100% protective efficacy against homologous CHMI conducted 9-10 weeks after the last dose of PfSPZ Challenge in the 3 clinical trials in which CQ PfSPZ Challenge was administered by DVI, CQ was the partner drug, and there was a CHMI. Protective efficacy was low when PfSPZ Challenge was administered ID (TIP 5) and when the partner drug was A / P (MALACHITE). There was not yet been a CHMI in 17-I-0067.

Table 3: PfSPZ-CVac Studies in Malaria- Naïve Adults

**Name**<br>**NCT No.**	**Site**<br>**Institution**	**First Injection**	**N**	**Route**	**Partner Drug**	**# of injections [Highest dose PfSPZ]**	**Best protection**[a]
TIP 5<br>NCT01728701	Nijmegen Radboud UMC	Sep 2012	20	ID	CQ	3 to 4 [$7.5x10^4$]	0% (0/20)

Name<br>NCT No.	Site<br>Institution	First<br>Injection	N	Route	Partner Drug	# of injections<br>[Highest dose PfSPZ]	Best protection[a]
TÜCHMI-002<br>NCT02115516	Tübingen<br>Inst for Trop Med	Apr 2014	45	DVI	CQ, or CQ + azithromycin	3 [5.12×10^4]	100% (9/9)<br>(CQ alone)
15-I-0169<br>NCT02511054	Bethesda<br>NIAID / LMIV	Nov 2015	20	DVI	CQ, or CQ + pyrimethamine	3 [5.12×10^4]	80% (4/5)<br>(CQ alone)
MALACHITE<br>NCT02858817	Tübingen<br>Inst for Trop Med	Nov 2016	21	DVI	Atovaquone / proguanil	3 [1.5×10^5]	25% (2/8)
11-0042<br>NCT02773979	Seattle<br>Group Health	Jan 2017	21	DVI	CQ	3 [1.024×10^5]	75% (6/8)
17-I-0067<br>NCT03083847	Bethesda<br>NIAID / LMIV	Jun 2017	14[b]	DVI	CQ, or pyrimethamine	3 [2×10^5]	pending
		Total subjects:	141				

[a] Percent protected (# protected/# undergoing CHMI)
[b] 37 more volunteers still to be immunized

2.6.11. PfSPZ-CVac immunization of malaria experienced adults in Africa.

There are two ongoing clinical trials in Mali and Equaotorial Guinea (Table 4). These two trials are both randomized, double blind, placebo-controlled trials, and the studies have not yet been unblinded. However, administration of investigational product has been well tolerated and safe in both trials with minimal numbers of adverse events.

Table 4: PfSPZ-CVac Studies in Malaria-Exposed Adults

Name	Site Institution	First Injection	N	Route	Partner Drug	# of Injections [Highest Dose PfSPZ]	Best Protection
15-0052<br>NCT02996695	Bougoula Hameau, Mali<br>U of Bamako, UMB-CVD, DMID	April 2017	62	DVI	CQ	3 [2.048×10^5]	Pending[a]
EGSPZV2<br>NCT02859350	Baney, Equatorial Guinea, EG Malaria Vaccine Initiative	Jan 2017	25	DVI	CQ	3 [1.0×10^5]	Results waiting for unblinding
		Total subjects:	87				

[a] Natural transmission

2.7. Summary of known and potential risks and benefits to human subjects of PfSPZ Challenge with CQ for PfSPZ0-CVac

2.7.1. Potential risks

Phlebotomy:

The maximum volume of blood drawn over the study period (≤800 ml over a 64 week period) is an accepted standard for healthy individuals that should not compromise the donor and is reflected in the German guidelines for blood donation [78]. According to these guidelines, the total blood volume sampled should not exceed 2000 mL (3000 mL for men) within 12 months and single donations should not exceed 500 mL. There may be minor bruising, local tenderness or pre-syncopal symptoms associated with venipuncture, which will not be documented as AEs if they occur. In rare cases, arteries or nervous tissue may be injured or a punctured vessel may occlude and induce inflammation of the surrounding tissue.

Administration of PfSPZ Challenge products and PfSPZ-CVac:

Serious allergic reactions including anaphylaxis have not been observed in the 860 individuals who have been inoculated with PfSPZ Challenge products as of January 1, 2018 (n =745 for CHMI and n =209 for PfSPZ-CVac approach, with 94 volunteers receiving both).

PfSPZ Vaccine has been given to 1130 volunteers, with maximum IV/DVI doses of $2.7x10^6$ and IM doses of $2.2x10^6$ radiation-attenuated PfSPZ per injection[27,28,(Seder & Lyke, unpublished data)].

Nevertheless, volunteers of CVac-Tü3 will be inoculated in a clinical area where physicians trained in Advanced Cardiac Life Support are present. Equipment and drugs are immediately available for the management of any severe or serious adverse reaction. Intravenous injection of up to 4,800 PfSPZ Challenge (7G8) was not associated with any injection-associated moderate, severe, or serious AE in any prior study. Injection of 51,200 PfSPZ Challenge (NF54) as part of PfSPZ-CVac approach has been associated with grade 3 symptoms in 3/8 volunteers in the Seattle study and in 0/28 in other studies, including TÜCHMI-002 and the trial conducted at the NIH Clinical Center by the Laboratory for Malaria Immunology and Vaccinology (LMIV). Injection of $1.0x10^5$ PfSPZ of PfSPZ Challenge (NF54) in malaria-exposed adults in Equatorial Guinea and of $2.048x10^5$ PfSPZ of PfSPZ Challenge (NF54) in malaria-exposed adults in Mali did not result in any symptoms reflecting the low levels of parasitemia developed by these semi-immune individuals. These studies have been done using chloroquine as the partner drug, in some cases supplemented by a second drug (pyrimethamine at NIH, long acting azithromycin at Tübingen). In CVac-Tü3, if some parasites do emerge from the liver despite CQ administration, early detection of parasitemia using PCR and use of NSAIDS in case will prevent the occurrence of any severe malaria symptoms (see Section 2.9.1 below).

Chloroquine:

Oral CQ intake in therapeutic or prophylactic doses is usually well tolerated. Reversible visual disturbances, tinnitus, loss of appetite, psychotic reactions, abdominal pain, nausea, and laboratory abnormalities may occur. Very rarely, drug induced hypersensitivity, cardiomyopathy, and acute severe reactions have been reported. During pregnancy and lactation CQ should not be given unless a high risk of malaria is present. In all other cases pregnancy and lactation constitute a contraindication for CQ use. The cumulative dose of CQ in the study will be well below dosing schedules of CQ or its derivatives used for autoimmune diseases[78] and similar to antimalarial chemoprophylaxis in medium-term travelers. The CQ-dose will be weight-adapted and will not exceed 10 mg/kg or 620 mg CQ base per dosing.

***Plasmodium falciparum* infection:**

Volunteers may develop symptoms of malaria during immunization by the PfSPZ-CVac approach, because blood-stages parasites can leave the liver and develop for a limited time until CQ is active.

In our previous studies one patient following immunization with PfSPZ under chloroquine developed a breakthrough infection (Mordmüller, unpublished – see section 2.7.2. – Case Report above). As described, the extensive investigations revealed that the chloroquine serum concentrations were under the detection limit. The patients refused to participate in further pharmacokinetical investigations. According to the investigators, the most likely cause of this infection is the previously undetected psychiatric intention to experience malaria, and the consequent probable non-compliance in taking chloroquine as prescribed. This was technically possible despite directly observed treatment (DOT), as detailed in the Case Report.

After inoculation with PfSPZ Challenge for CHMI, those not protected are likely to develop malaria. Symptoms and signs of malaria may include fever, tachycardia, hypotension, chills, rigors, sweats, headache, anorexia, nausea, vomiting, diarrhea, myalgia, arthralgia, low back pain, thrombocytopenia, and lymphopenia. Thirty to 50 percent of volunteers are likely to experience at least one severe (Grade 3) adverse event related to Pf infection[61]. Unmonitored and untreated, Pf infection can be fatal – however – only after several additional replication cycles of 48 hours each after the onset of symptoms. For this reason, volunteers will be enrolled in the study only if they are deemed reliable and capable of complying with the intensive follow-up schedule (section 6). If necessary, volunteers can be admitted for in-patient care at the University Hospital in Tübingen.

Treatment of *P. falciparum* infection:

After CHMI, volunteers will be treated with a registered, oral, proven, and highly efficacious treatment under supervision of a study team member. First-line treatment will be A/P. Patients will be treated with a once daily dose of 1000mg Atovaquon/400 mg Proguanilhydroclhorid for 3 days. The PfSPZ Challenge parasite strain Pf 7G8 is the second most used CHMI strain, although numbers are much smaller than for NF54/3D7 [26,56]. 7G8 retains full sensitivity to the first and second line drugs of this trial (atovaquone-proguanil and artemether-lumefantrine, respectively) but it is CQ, pyrimethamine, cycloguanil and sulfadoxine resistant.

Despite the fact that all well documented volunteers since 1985 were successfully treated, a small probability of drug failure remains (for 1,343 successfully treated volunteers the 97.5% binomial exact confidence interval is 0 to 0.27%). In case of drug failure or inability to receive oral medication, an alternative parental treatment will be given according to German guidelines for the treatment of malaria[79].

2.7.2. Potential benefits

Volunteers will not benefit directly from participation in this study. Information about their general health status may be a potential indirect benefit. Volunteers will be treated as malaria-naïve in case they travel to malaria endemic areas afterwards. However, it is hoped that the information gained from this study will contribute to the development of a safe and effective antimalarial intervention. Translation of CPS into the PfSPZ-CVac approach with a simple protocol and optimized drug administration will be the basis for further product development of a highly efficacious malaria vaccine candidate, which would improve conditions for inhabitants of malaria-endemic countries as well as travelers and migrants to malaria-endemic countries.

2.8. Rationale for conducting the trial

No highly efficacious malaria vaccine (>75% protective efficacy over at least 6 months) is yet available. The most advanced malaria vaccine candidate is RTS,S, with a protective efficacy against clinical malaria of between 56% in children and 31% in infants using time-to-event analysis [10,11]. Proportional efficacy against malaria is much lower. In contrast, infection with live, fully infectious PfSPZ under CQ chemoprophylaxis provides high-level protection against subsequent challenge over an extended time period[37,38,40].

TÜCHMI-001 previously showed that aseptic, purified, vialed, cryopreserved PfSPZ (PfSPZ Challenge (NF54)) consistently infect malaria-naïve individuals at a dose of 3.2×10^3 PfSPZ administered IV.[63] Hence, PfSPZ Challenge products are a prime candidate for replacement of mosquito bites in CPS to establish a vaccine. This approach to vaccination is called PfSPZ-CVac (PfSPZ Chemoprophylaxis Vaccine). The dose of 5.12×10^4 PfSPZ of PfSPZ Challenge (NF54) that is administered three times in 4 week-intervals with CQ chemoprophylaxis achieved protective immunity in 100% of study participants in TÜCHMI-002 trial[40]. However, a chemoprophylactic regimen with a simplified regimen is required.

The rationale of the trial is to optimize the chemoprophylaxis of the immunization regimen of PfSPZ-CVac and to evaluate protection against heterologous CHMI.

Chemoprophylactic regimen selection

To develop the PfSPZ-CVac approach into a product for application for vaccination, the prophylactic regimen needs to be safe, easily administered, and able to prevent clinical manifestations of malaria. CQ has previously shown protective efficacy against the development of malaria (parasitemia, detected by microscopy) in CHMI model[40] and can be given concurrently with the DVI administration of PfSPZ.

2.9. Rationale of PfSPZ Challenge (NF54), PfSPZ Challenge (7G8), chloroquine dosing schedule

2.9.1. Timing and dosing of PfSPZ Challenge (NF54) under chemoprophylaxis

In our previous study, data from the trial in Barcelona, Spain and trials with PfSPZ Vaccine (which is the same product as PfSPZ Challenge (NF54) but not replicating due to irradiation before cryopreservation) a strong association between dose and vaccine efficacy as well as immunological correlates of protection was observed[63,64]. We hypothesize a dose-response relationship between PfSPZ dose and the level of protection, moving from minimal protection to long-term protection, even against heterologous infection. TÜCHMI-002 Stage A showed 100% protective efficacy using $5.12x10^4$ PfSPZ IV at 4 week intervals[40]. In TÜCHMI-002 Stage B shorter immunization intervals (2 weeks and 5 days) using three times $5.12x10^4$ PfSPZ were assessed.–Preliminary results indicate that i) 2 g extended release azithromycin (Zithromax Uno) does not prevent parasite egress form the liver, ii) efficacy of shortened immunization regimen decreases compared to the 8 week schedule of Stage A, iii) a ten day immunization schedule is still efficacious (63% vaccine efficacy). In CVac-Tü3, PfSPZ Challenge (NF54) will be administered three times at Days 0, 5 and 28. This immunization schedule would be an acceptable regimen for use in travel medicine practice[36,38] and in endemic regions. The initial dosage of PfSPZ Challenge (NF54) for each administration will be $1.1x10^5$ PfSPZ, double the dose that led to high-grade protection in TÜCHMI-002 when administered at 4 week intervals, but only 63% to 67% protection when administered at shorter intervals[40]. When 3 doses of $1.0x10^5$ were administered at 5 day intervals in the Seattle trial (see above), protection against homologous CHMI was 75%. We believe that delaying the third dose until 28 days will further increase protection, but that protection against Pf7G8 will require 3 doses at least of $1.0x10^5$ PfSPZ, but may require more. However, first we need to determine if $1.1 x10^5$ PfSPZ is adequate to achieve cross-protection against other *Plasmodium* strains and species and longevity of immunity.

2.9.2. Dosing of PfSPZ Challenge (7G8) for CHMI

To assess immunization success, volunteers will be inoculated with $3.2x10^3$ PfSPZ Challenge (7G8), the fully infective (>95%) dose for 21, malaria-naive research subjects undergoing CHMI. All volunteers will have CHMI with PfSPZ Challenge (7G8). The first CHMI will be done 10 weeks, the second CHMI will be done 26 weeks and the third CHMI will be done 52 weeks after the third immunization.

2.9.3. Dosing of Chloroquine

CQ will be administered orally, 30 to 60 minutes prior to each of three PfSPZ immunizations at a dose of 10 mg/kg. The schedule is designed to achieve optimal systemic CQ concentration at the time when asexual parasites leave the liver and the first blood-stage cycle starts. The pharmacokinetic profile of CQ has been investigated extensively[80],[81] and provides good evidence for the proposed regimen.

2.10. Rationale for route administration

CQ will be given orally according to the summary of product characteristics. PfSPZ Challenge products will be administered intravenously by DVI. This is the route that generates the least variation in the number of infected liver cells, works with the lowest number of PfSPZ, and gives the shortest time to parasitemia (pre-patent period) compared to other routes of administration. CHMI to assess immunization success is performed by DVI of $3.2x10^3$ PfSPZ Challenge (NF54). This dose and mode of administration were shown to infect 56/56 non-immunized, malaria-naïve or minimally malaria-exposed volunteers as of January 1, 2018, and this method for CHMI is technically robust, safe, and tolerable. It has become the established infection model in interventional trials used to test vaccines as well as drugs against malaria.

3. Trial objectives and purpose

3.1. Study overview

CVac –Tü3 is a single center, randomized, placebo-controlled, double-blinded, trial using PfSPZ Challenge (NF54) under CQ chemoprophylaxis for immunization and PfSPZ Challenge (7G8) for sequential controlled human malaria infection (CHMI), designed to establish a PfSPZ-CVac regimen with CQ that:

1. Is safe and well tolerated.
2. Provides consistent sterile protection against repeat CHMI with heterologous PfSPZ in healthy adult subjects 10 weeks, 26 weeks and 52 weeks following immunization
3. Is a practical preventive regimen for malaria.

Volunteers will receive three immunizing PfSPZ Challenge (NF54) DVI injections of 1.1×10^5 PfSPZ per dose while taking oral chemoprophylaxis. Immunizations are given under a chemoprophylactic regimen with CQ 10 mg/kg administered simultaneously on days of PfSPZ immunization. Control groups will receive normal saline (0.9% NaCl) IV as placebo under the respective chemoprophylactic regimen.

3.2. Trial objectives

Primary Objective:

1. Establish an immunization regimen of three injections of intravenous PfSPZ Challenge (NF54) by DVI and oral CQ chemoprophylaxis administered on Days 0, 5 and 28, which is safe and well tolerated.

Secondary Objective:

Establish an immunization regimen of PfSPZ Challenge (NF54) under CQ chemoprophylaxis, which provides protection against repeat CHMI with heterologous PfSPZ Challenge (7G8) in healthy adult subjects 10, 26 and 52 weeks following the last immunization.

Explorative Objectives:

1. Identify parasitological and immunological correlates of protection against CHMI.
2. Characterize B- and T-cell response and repertoire to immunization with PfSPZ-CVac.
3. Assess parasite kinetics following CHMI.
4. Identify psychological changes of the volunteers during the study period

3.3. Duration of the trial

The duration of participation for a single volunteer is approximately 16 months (71 weeks) after first immunization. The date of signature of the informed consent by the first volunteer defines the study start. The date of last visit of the last volunteer defines the end of the follow-up period.

Total trial duration: approx. 713 days

- Trial duration for individual patient: approx. 497 days
- Study treatment: 420 days (4 phases with 28 days of intensive follow-up)
- Post CHMI Follow-up: 56 days
- Number of visits: 97
- FSI (First Subject In): Day -28
- LSI (Last Subject In): Day -3
- LSO (Last Subject Out): Day 497
- DBL (Data Base Lock): 4 months after LSO
- Statistical Analyses Completed: 5 months LSO
- Trial Report Completed: 6 months after LSO

4. Trial design

4.1. Type and design of the trial

CVac-Tü3 is a single center, randomized, placebo-controlled, double-blinded, PfSPZ-CVac trial with repeated CHMI. PfSPZ Challenge (NF54) has an established infective dose of $3.2x10^3$ PfSPZ. PfSPZ Challenge (NF54) contains the Pf strain NF54, and when used both for immunization and CHMI, the CHMI is considered homologous. PfSPZ Challenge (7G8) contains the Pf clone 7G8. It has an infective dose of $3.2x10^3$ PfSPZ. When PfSPZ Challenge (NF54) is used for immunization and PfSPZ Challenge (7G8) for CHMI, the CHMI is considered heterologous.

Placebo or PfSPZ Challenge (NF54) with $1.1x10^5$ PfSPZ (n = 14) will be given by DVI at Days 0, 5 and 28. On the days of immunization, 10 mg/kg CQ will be administered orally to all participants 30 to 120 minutes before injection with PfSPZ Challenge (NF54). Control volunteers (n=7) receive normal saline (0.9% NaCl) by DVI instead of PfSPZ. Normal saline is indistinguishable from PfSPZ formulations when supplied as a charged syringe for injection. Treatment allocation is random and blinded with a ratio of 2:1 (PfSPZ: placebo). There will be a total of 14+7 volunteers, 21 in total.

Ten weeks after the third immunization (at week 14), the first CHMI will be made. The second CHMI will be made 26 weeks after the last immunization (at week 30). 52 weeks after the third immunization (at week 56), the third CHMI will be made (at week 56). All CHMIs will be heterologous, done with $3.2x10^3$ PfSPZ of PfSPZ Challenge (7G8) given by DVI, respectively.

Table 5: Immunization and CHMI scheme

Group	Day 0	Day 5	Day 28	Week 14	Week 30	Week 56
	$1.1x10^5$ PfSPZ	$1.1x10^5$ PfSPZ	$1.1x10^5$ PfSPZ	CHMI I	CHMI II	CHMI III

All PfSPZ injections are given intravenously; CHMI: controlled human malaria infection.

Volunteers will be healthy adults between 18 and 45 years of age. Safety and tolerability data will be collected for all volunteers. Volunteers and the clinical team will be blinded to allocation of volunteers to placebo or PfSPZ Challenge (NF54) for immunization.

4.2. Endpoints

Safety endpoint

Number or occurrence of at least possibliy related Grade 3-4 AEs and serious adverse events SAEs from time of first administration of CQ until the end of the study.

Efficacy endpoint

Proportion of protected volunteers.

Evaluative Measure: Protection is defined as the absence of parasites in the peripheral blood for 28 days following first, second and third CHMI with PfSPZ Challenge (7G8) in volunteers receiving PfSPZ-CVac with CQ. Parasitemia as a CHMI endpoint resulting in treatment is defined as at least one qPCR result above 100 parasites per mL among three positive results at least 12 hours apart or as a positive thick blood smear.

Parasitemia is monitored by daily thick blood smears and qPCR between +6 and +21 days, and +28 days during the period following DVI for CHMI. Statistical testing is done hierarchically: 1) protection against first CHMI, 2) protection against second CHMI, 3) protection against third CHMI.

Exploratory efficacy endpoints

- Time-to-parasitemia in volunteers who receive immunization using PfSPZ Challenge (NF54) or placebo under CQ chemoprophylaxis and become parasitemic within +28 days following CHMI with PfSPZ Challenge (7G8).
- Time-to-parasitemia in placebo recipients following second versus first CHMI, and third versus second CHMI (carry-over effect).

4.3. Minimization of bias

4.3.1. Randomization

Randomization will be done on the day of first immunization (I) prior to injection. Seven volunteers will be allocated randomly to get placebo (NaCl 0.9% for injection) instead of PfSPZ immunizations (n = 14). A dedicated member of the formulation team, who is not involved in volunteer management or diagnostic activities, will keep the randomization envelopes and dosing schedule. A third party outside the study team and sponsor will generate and distribute the randomization list using a random number generator (Mersenne-Twister implemented in R; www.r-project.org).

4.3.2. Blinding and allocation concealment

The study is double-blinded (neither volunteer nor clinical and diagnostic teams are aware of intervention allocation). Only the PfSPZ formulation team will be aware of the intervention allocation. No individual of the PfSPZ formulation team will be involved in clinical or diagnostic activities. Syringes will be labeled with the volunteer ID (VID), date and number of injection, but not with a label that indicates its content. Delivery of syringes from the PfSPZ preparation team to the clinical team will be done via a shielded box. PfSPZ Challenge (NF54) and placebo are both clear fluids and not distinguishable by appearance.

4.4. Treatments

4.4.1. PfSPZ Challenge (NF54) for immunization

The PfSPZ-CVac approach consists of two components: PfSPZ Challenge (NF54) and an antimalarial (CQ).

PfSPZ Challenge (NF54) is a suspension of aseptic, purified, live and infectious, cryopreserved Pf sporozoites (PfSPZ) formulated in cryoprotectant and dispensed in vials. PfSPZ Challenge product is stored in liquid nitrogen vapor phase (LNVP) at -150°C to -196°C, thawed, diluted in phosphate buffered saline (PBS) with human serum albumin (HSA) to achieve the correct dosage and administered by DVI. Sanaria's quality program meets regulatory requirements and adheres to U.S. FDA Good Manufacturing Practice (GMP) regulations (21 CFR 211).

PfSPZ Challenge (NF54) product is stored at Sanaria or Sanaria's designated storage site in LNVP at -150°C to -196°C until shipped to the clinical study site. Shipment is in compliance with all US FDA, US Department of Transportation, German, and United Nations transport guidelines for shipping bio-hazardous materials like malaria parasites in LNVP. Transfer of PfSPZ Challenge (NF54) from its storage site to the clinical trial site will follow relevant Sanaria's SOPs, and will be under constant temperature monitoring. The interior chamber temperature of the LNVP dry shipper will be continuously monitored and recorded, with documentation of a full chain of custody on a 'Shipping Log'. Receipt of PfSPZ Challenge (NF54) product is documented at the study site on a 'Tracking Log' by study staff.

Qualified and trained vaccine dilution and preparation staff will prepare the test article for each dose just prior to administration as described in the relevant SOPs. Immediately before use, cryopreserved vials will be thawed per SOP. PfSPZ Challenge (NF54) is administered according to an SOP by direct intravenous inoculation (DVI). During administration of PfSPZ Challenge (NF54), Advanced Cardiac Life Support drugs and resuscitation equipment will be immediately available for the management of anaphylaxis or other severe or serious AEs.

4.4.2. Placebo

In CVac-Tü3, immunization by PfSPZ Challenge (NF54) will be controlled using a placebo injection. Since PfSPZ Challenge (NF54) dilutions are clear, non-viscous fluids, 0.9% NaCl for injection is used as placebo. 0.9% NaCl for injection is purchased locally from a pharmacy. CQ treatment is administered as in the respective *verum* group.

4.4.3. PfSPZ Challenge (7G8) for CHMI

CHMI is given +10 weeks, +26 and +52 weeks after the last PfSPZ Challenge (NF54) immunization or placebo administration. PfSPZ Challenge (7G8) are prepared as described above (4.4.2).

PfSPZ Challenge (7G8) product is administered by DVI, at a dose of $3.2x10^3$ parasites. Volunteers will be treated either when parasitemic by microscopy or based on a positive qPCR reading for three consecutive timepoints at least 12 hours apart (with one sample more than 100 parasites/microliter). Treatment will be the same as in our previous study of PfSPZ Challenge (7G8) (MAVACHE) using A/P or artemether/lumefantrine as first line treatment for Pf infection. Antimalarial drugs are given according to German guidelines for the treatment of uncomplicated Pf malaria[79]. 7G8 parasites contained in PfSPZ Challenge (7G8) are sensitive to artemether/lumefantrine, A/P and mefloquine, and these regimens have been used to successfully treat volunteers infected with PfSPZ Challenge (7G8) (Tables 3 and 4).

PfSPZ Challenge (7G8) is manufactured, shipped, formulated and injected exactly as PfSPZ Challenge (NF54). The Pf7G8 clone is not sensitive to CQ and pyrimethamine but highly sensitive to artemether-lumfantrine and atovaquone-proguanil.

All antimalarial treatments are given under direct observation of the clinical team. In case a volunteer cannot take oral antimalarial treatment a standard parenteral treatment will be given (e.g. artesunate IV). If volunteers become parasitemic, two consecutive negative slide readings one day apart are required in order to document cure.

Table 6: Generic and brand names of first and second line drugs and their use in previous CHMI trials

Generic name	Brand name	Manufacturer	ClinicalTrials.gov ID
Atovaquone/Proguanil	Malarone	GlaxoSmithKline	NCT01086917, NCT01465048
Artemether/Lumefantrine	Riamet	Novartis Pharma	NCT01086917, NCT01624961
Mefloquine	Lariam	Roche	[61,79]
Chloroquine	Resochin	Bayer Schering Pharma	NCT01546389

4.4.4. PfSPZ Challenge product diluent

The diluent for both PfSPZ Challenge products is composed of phosphate buffered saline (PBS) and human serum albumin (HSA). Vials of PBS and HSA will be shipped to the clinical site, where diluent composed of PBS and HSA will be prepared.

PBS is manufactured in compliance with good manufacturing practice (GMP). A certificate of analysis is supplied for each lot of PBS. The PBS is stored at ambient temperature in a controlled room.

Sanaria purchases HSA (25%) approved for parenteral, intravenous administration to humans. Every purchased lot of HSA is supplied with a Certificate of Analysis that is reviewed and approved upon receipt at Sanaria Inc. before release for clinical use. HSA is aliquoted and repackaged at Sanaria, and released for clinical use with a Certificate of Analysis. HSA vials are stored at ambient temperature in a controlled room.

4.4.5. Minimizing environmental contamination with PfSPZ

Material used to prepare, administer, and manipulate the site of injection (dressings, needles, catheter) of PfSPZ Challenge products will be disposed of according to relevant guidelines to handle potentially infectious material[82]. The risk of infecting *Anopheles* mosquitoes in the study area through biting of study participants is theoretically possible after PfSPZ Challenge (NF54) or PfSPZ Challenge (7G8) administration for CHMI but probability is extremely low since gametocyte maturation is not detectable even by highly sensitive molecular methods in CHMI and CPS experiments[83]. During immunization, parasites are not expected to circulate and hence cannot infect mosquitoes.

4.4.6. Chloroquine

CQ is a registered drug and is obtained via the hospital pharmacy. It is provided as tablets of CQ phosphate (Resochin, Bayer Schering Pharma). All doses are calculated in terms of the absolute value of CQ base using a dosing table (Table 7). Each tablet contains 155 mg of CQ base (250 mg CQ phosphate). Dosing is 10 mg/kg 30 to 120 minutes prior to PfSPZ Challenge injection. The maximal dose per day will be 620 mg CQ base (4 tablets) irrespective of body weight (Table 7). Resochin is the CQ brand available and registered in Germany and dosing according to the proposed scheme will minimize CQ-related side effects.

Table 7: Chloroquine dosing table using Resochin, Bayer Schering Pharma

Body weight (kg)	10 mg/kg (number of tablets)
16–30	1
31–46	2
47–62	3
63–77	4
78–93	4
>94	4

Drug Accountability; Therapy Compliance and Disposal

The IP or placebo, and corresponding Resochin prophylaxis will be dispensed to the subjects by the investigator/s. The investigator or delegate will document the date of dispensary, subject identification, batch/ serial numbers or other identification of IP, placebo and Resochin. The investigator will also keep accurate records of the quantities of each administered to each subject. The site monitor will periodically check the supplies of trial medication held by the investigator to ensure the correct accountability of all IP, placebo and Resochin used. At the end of the trial, all unused IP will be returned to Sanaria Inc.

4.4.7. Rescue and other treatment

In addition to planned treatment during immunization and after CHMI, administration of an antimalarial is given:

- Based on recommendations of the clinical team, the local safety monitor or the SMC.
- All volunteers who receive $1.1x10^5$ PfSPZ of PfSPZ Challenge are expected to have parasitemia detectable by PCR. Some volunteers will have parasitemia detectable by microscopy and symptoms consistent with malaria between +7 and +11 days after administration of PfSPZ Challenge. It is expected that CQ in the blood will be adequate to clear the parasitemia and the symptoms will be self-limited. However, at the discretion of the clinical team such volunteers could be treated. Two scenarios where treatment is likely to be initiated are,
  - If a volunteer has microscopically detectable parasitemia and grade 3 or 4 symptoms consistent with malaria for 2 days during the immunization period, the clinical team may opt for treatment.

- If a volunteer has persistent symptoms and the parasitemia level (by thick blood smear or qPCR) is rising over several days during the immunization period, the clinical team may opt for treatment.

- Following CHMI, treatment can be initiated based on qPCR results (positive reading three consecutive timepoints at least 12 hours apart, with one sample more than 100 parasites/microliter) or on positive thick blood smear reading.
- If a volunteer withdraws or is withdrawn from the study after receiving a PfSPZ Challenge product at one or more of the three immunizations or at CHMI; in this case a full, appropriate, curative course of antimalarial therapy must be completed. The timing of treatment should be decided prospectively with the SMC, and the Sponsor and needs to be accompanied by a full report explaining reason, timing, and compounds used for treatment. A chemotherapeutic treatment should not be started earlier than 7 days after PfSPZ Challenge product administration to expose asexual erythrocytic stage parasites that are known to be sensitive to the antimalarials (Tables 3 and 4).
- If a patient is unable to tolerate an oral antimalarial, the volunteer will have appropriate parenteral antimalarial therapy.
- In some volunteers highly sensitive qPCR is able to detect low levels of parasite nucleic acids beyond one week following treatment which slowly decrease until +56 days after inoculation. In order to discriminate low-grade gametocytemia from circulating nucleic acids, the PI may decide to recommend a gametocytocidal course of primaquine.

4.4.8. Supportive medications

On development of symptoms, provided there are no contraindications, volunteers will be permitted to take antipyretic medication (e.g. ibuprofen) and other medications such as anti-nausea medication at the discretion of the clinical investigators.

4.4.9. Criteria for hospital admission

The decision regarding admission will be made by the investigators in conjunction with the physician on call at the University hospital. Nevertheless, any physician may decide upon hospital admission at his/her discretion. Any non-malaria related symptoms will be treated according to German and international guidelines. Treatment of severe malaria will be done according to national and international guidelines[82] and after consultation with infectious diseases and tropical medicine specialists. Severe malaria has never occurred in a CHMI experiment so far and will automatically lead to hospitalization. We follow the definition of the WHO[82] that defines severe malaria as the occurrence of Pf parasitemia and at least one of the following clinical or laboratory criteria adapted to be appropriate for an adult malaria-naïve population:

- Impaired consciousness or unarousable coma.
- Prostration, i.e. generalized weakness so that the patient is unable to walk or sit up without assistance.
- Failure to feed (be able to eat).
- Multiple convulsions – more than two episodes in 24 hours.
- Deep breathing, respiratory distress (acidotic breathing).
- Circulatory collapse or shock, systolic blood pressure <70 mm Hg.
- Clinical jaundice plus evidence of other vital organ dysfunction.
- Hemoglobinuria.
- Abnormal spontaneous bleeding.
- Pulmonary edema (radiologically confirmed).
- Hypoglycemia (blood glucose <2.2 mmol/L or <40 mg/dL).
- Metabolic acidosis (plasma bicarbonate <15 mmol/L).
- Hyperparasitemia (>2% parasitemia or 1.0×10^5 parasites per µl).

- Hyperlactatemia (lactate > 5mmol/L).
- Renal impairment (serum creatinine >265 μmol/L).

In addition, if any of the following criteria are met, hospital admission will be considered:

- Failure of Grade 3 or 4 symptoms to improve within 48 hours of starting antimalarial therapy.
- Unable to tolerate oral treatment (e.g. repeated vomiting).
- Dehydration requiring intravenous fluid therapy.
- Clinical signs or symptoms suggestive of pulmonary edema.
- Any sign or symptom of neurological dysfunction.
- Any sign, symptom or laboratory evidence of significant renal dysfunction.
- Unanticipated concern about subject's home circumstances. In this case hospital admission is not considered a serious adverse event.
- Any other significant finding which the Investigator feels warrant inpatient admission.

5. Selection and withdrawal of trial volunteers

5.1. Volunteers

Volunteers may be recruited by use of an advertisement form approved by the ethics committee and distributed or posted in the following places:

- In public places with the agreement of the owner or proprietor.
- In newspapers or other literature for circulation.
- As a press release through the press department of the Institute of Tropical Medicine and the University of Tübingen.
- On a website operated by our group or with the agreement of the owner or operator.
- On stalls or stands at exhibitions or fairs.
- Via interviews and presentations (e.g. presentations at lectures or invited seminars).
- Via university mailing list.

5.2. Informed consent

All volunteers will sign and date the informed consent form before any study specific procedures are performed. An information sheet will be made available to the volunteer at least 24 hours prior to the screening visit. At the screening visit, the volunteer will be fully informed of all aspects of the trial, the potential risks and their obligations. The following general principles will be emphasized:

- Participation in the study is entirely voluntary.
- Refusal to participate involves no penalty or loss of medical benefits.
- The volunteer may withdraw from the study at any time.
- The volunteer is free to ask questions at any time to allow him or her to understand the purpose of the study and the procedures involved.
- There is no direct benefit from participating.

The aims of the study and all tests to be carried out will be explained. The volunteer will be given the opportunity to ask about details of the trial, and will then have time to consider whether or not to participate. If they do decide to participate, volunteers will be asked to complete a questionnaire testing their understanding of the trial. This helps to ensure that individuals understand the trial sufficiently to give informed consent. Provided the volunteer answers all questions in the questionnaire correctly, she/he will be asked to sign and date two copies of the consent form, one for them to take away and keep, and one to be stored at the site together with the case report form

(CRF). These forms will also be signed and dated by the Investigator. Volunteers who fail to answer all questions correctly on their first attempt will be allowed to re-take the questionnaire once following further discussion with the investigator. Provided they subsequently answer all questions in the quiz correctly they may then complete the consent form and be screened for the trial.

5.3. Inclusion criteria

The volunteer must satisfy all the following criteria to be eligible for the study:

- Healthy adults aged 18 to 45 years.
- Able and willing (in the Investigator's opinion) to comply with all study requirements.
- Willing to allow the investigators to discuss the volunteer's medical history with their general practitioner if required.
- Residence in Tübingen or surroundings for the period of the trial.
- Women only: Must agree to practice continuous effective contraception for the duration of the study (a method which results in a low failure rate; i.e. less than 1% per year).
- Agreement to refrain from blood donation during the course of the study and after the end of their involvement in the study according to the local and national blood banking eligibility criteria.
- Provision of written informed consent to receive PfSPZ Challenge products for immunization and subsequently for CHMI.
- Reachable (24/7) by mobile phone during the immunization and CHMI period.
- Willingness to take CQ during immunization and a curative antimalarial regimen following CHMI.
- Agreement to stay overnight for observation during the period of intensive follow-up post-challenge if required.
- Answer all questions on the informed consent quiz correctly.
- A body mass index <35.

5.4. Exclusion criteria

The volunteer may not enter the study if any of the following apply:

- History of *P. falciparum* malaria.
- Residence in a malaria endemic area for 5 or more years continuously.
- Prior receipt of malaria vaccine.
- Planned travel to malaria endemic areas during the study period.
- Use of drugs with known antimalarial activity within 30 days of study enrollment (e.g. trimethoprim-sulfamethoxazole, doxycycline, tetracycline, clindamycin, erythromycin, fluoroquinolones, or azithromycin).
- Receipt of an investigational product in the 90 days preceding enrollment, or planned receipt during the study period.
- HIV infection.
- Any confirmed or suspected immunosuppressive or immunodeficient state (e.g. repeated and/or unusual infections), history of infection caused by opportunistic organisms any infection or combination of infections that suggest underlying immunodeficiency, history of meningitis, encephalitis, septic shock, life-threatening soft tissue infection, more than one pneumonia, asplenia and/or chronic (more than 14 days) immunosuppressant medication within the past 6 months (inhaled and topical steroids are allowed)).
- Use of immunoglobulins or blood products within 3 months prior to enrollment.
- Known (or signs consistent with) sickle cell anemia, sickle cell trait, thalassemia or thalassemia trait, glucose-6-phosphate dehydrogenase deficiency.
- Pregnancy, lactation or intention to become pregnant during the study.

- Contraindications to the use of the following antimalarial medications: A/P, artemether-lumefantrine, CQ, artesunate.
- History of cancer (except basal cell carcinoma of the skin and cervical carcinoma in situ).
- History of serious psychiatric condition that may affect participation in the study.
- Any other serious chronic illness requiring hospital specialist supervision.
- Suspected or known current alcohol abuse as defined by an alcohol intake of greater than 60 g (men) or 40 g (women) per day or a carbohydrate deficient transferrin (CDT) level ≥2.5%.
- Suspected or known injected drug abuse in the 5 years preceding enrollment.
- Positive for hepatitis B surface antigen (HBs-antigen).
- Seropositivity for hepatitis C virus (antibodies to HCV)
- Falling in moderate risk or higher categories for fatal or non-fatal cardiovascular event within 10 years (>10%) determined by non-invasive criteria for cardiac risk (SCORE[84]).
- Abnormal electrocardiogram on screening: pathologic Q wave and significant ST-T wave changes, left ventricular hypertrophy, clinically significant arrhythmias, left bundle branch block, secondary or tertiary AV block
- A QT/QTcB interval >450 ms.
- Retinal abnormalities.
- Known or suspected porphyria.
- Volunteers unable to be closely followed for social, geographic or psychological reasons.
- CrCL <30ml/min.
- History of seizure (except uncomplicated febrile convulsion at childhood)
- Immunization with more than 3 other vaccines within four weeks.
- Any other significant disease, disorder, finding at medical history, biochemistry, hematology tests, urine analysis results or at clinical examination which, in the opinion of the investigator, may significantly increase the risk to the volunteer because of participation in the study, affect the ability of the volunteer to participate in the study or impair interpretation of the study data.

In case of inconclusive results of laboratory tests or other diagnostic procedures, the test will be repeated. If doubts about the results persist, volunteers will be considered ineligible.

5.5. Withdrawal of volunteers

In accordance with the principles of the current revision of the Declaration of Helsinki (updated 2013) and any other applicable regulations, a volunteer has the right to withdraw from the study at any time and for any reason, and is not obliged to give his or her reasons for doing so. The Investigator may withdraw the volunteer at any time in the interests of the volunteer's health and well-being. In addition, the volunteer may withdraw/be withdrawn for any of the following reasons:

- Administrative decision by the Investigator.
- Ineligibility (e. g. arising during the study).
- Significant protocol deviation.
- Volunteer non-compliance with study requirements.
- An AE, which requires discontinuation of the study involvement or results in inability to continue to comply with study procedures.

The reason for withdrawal will be recorded on the CRF. If withdrawal is due to an AE, appropriate follow-up visits or medical care will be arranged, with the agreement of the volunteer, until the AE has resolved, stabilized or a non-trial related causality has been assigned. Any volunteer who is withdrawn from the study may be replaced, if that is possible within the specified time frame. The Local Safety Monitor (LSM) may recommend withdrawal of volunteers.

If a volunteer withdraws/is withdrawn from the study after receiving PfSPZ Challenge products but before a potential malaria diagnosis, a complete, appropriate, curative course of antimalarial therapy must be completed during (early) blood-stage of the infection, which means that treatment is initiated not earlier than +7 days after inoculation of a PfSPZ Challenge product. This is done to ensure that blood-stage parasites are exposed to the antimalarial drug and not insensitive pre-erythrocytic stages. The importance of this will be emphasized to volunteers at screening.

If a volunteer withdraws from the study, blood samples collected before their withdrawal from the trial will be used/stored unless the volunteer specifically requests otherwise. Data from volunteers withdrawn from the study before fulfilling the criterion for malaria diagnosis will be excluded from the analysis of results relating to the study's primary objective. Data from volunteers withdrawn from the study after fulfilling the criterion for malaria diagnosis will be included in analysis of results relating to the study's primary objective. All volunteers can decide at any time if samples and data may be used later or will be permanently destroyed. This will be explained and documented in a separate data protection form.

5.6. Pregnancy

Should a volunteer become pregnant during the trial, she will be followed up as other volunteers and in addition will be followed until pregnancy outcome. No further injection of a PfSPZ Challenge product (neither for immunization nor for CHMI) will be done on such volunteers and blood sampling will be reduced to the clinically relevant minimum, e.g. to prove complete parasite clearance. The management of any volunteers found to be pregnant at the point of diagnosis of malaria will be done according to national and international guidelines [85] and in agreement with the infectious disease consultant. All pregnancies that occur during the trial will be reported to the Sponsor as soon as they are diagnosed.

6. Trial methods and study procedures

Details on PfSPZ Challenge products, placebo, and antimalarial treatments are given in section 4.4.

6.1. Study procedures

Procedures will be performed at the time points indicated in the schedule of procedures (Tables 8A-B). The three immunization days are labeled as follows: First immunization (I), second immunization (II) and third immunization (III). Within the schedule of procedures CHMI is abbreviated as 'C'. Any numbers following these capital letters indicate the days from injection of a PfSPZ Challenge product (e.g. I-1: one day prior to first immunization; III5: +5 days after the third immunization). Additional procedures or laboratory tests may be performed at the discretion of the investigators if clinically necessary.

In addition to frequent contacts at the study center, participants will be provided with a diary and a thermometer on the day of first immunization injection and asked to take notes on any observation at any time point they make regarding their health status during the course of the trial. These observations include body temperature, reactions at the injection site as well as general symptoms such as headache, diarrhea, vomiting or chills.

Participant flow

All participants will receive CQ 30 to 120 minutes prior to administration of PfSPZ Challenge (NF54) or 0.9% NaCl, and will be assessed to insure that they have swallowed and not vomited the CQ. Participants will be randomized to receive either PfSPZ Challenge (NF54) for immunization or 0.9% NaCl by DVI. Volunteers will remain in the clinic for at least 30 minutes after their injection. For CHMI, PfSPZ Challenge (7G8) is administered at a dose of $3.2x10^3$ PfSPZ, by DVI followed by an observational period of 30 minutes before leaving the clinic.

All samples for exploratory downstream analyses will be anonymized by allocation of random numbers and investigated without knowledge of group assignment or who the individual is. All participants will be followed and sampled using the same schedule.

If there are safety concerns where the clinical team feels independent advice or review is important, an SMC meeting will be convened prior to continuation of the trial.

6.1.1. Sequence of events

Screening (SCR)

All potential volunteers who give informed consent will undergo a screening visit, which may take place within 28 days prior to first immunization. In any case, all laboratory values need to be present and reviewed before the start of immunization. Information and consenting will be done before screening as described. If consent is obtained, the screening procedures indicated in the schedule of procedures will be undertaken. Abnormal clinical findings from the medical history, physical examination or blood tests at any point in the study, will be assessed using established reference intervals of the laboratory in charge of the Universitätsklinikum Tubingen. If a laboratory test is out of range it may be repeated to ensure it is not a single occurrence. If an abnormal finding is deemed to be clinically significant, the volunteer will be informed and appropriate medical care arranged with the permission of the volunteer. Decision to exclude the volunteer from enrolling in the trial or to withdraw a volunteer from the trial will be at the discretion of the Investigator following procedures for AE management as described in section "Adverse event assessment and management". Cardiac risk is assessed by published non-invasive criteria. Volunteers who do not belong to the two lowrisk categories are not eligible. In addition, a 12-lead electrocardiogram is performed in each volunteer on screening. The level of carbohydrate-deficient transferrin will be measured to assess chronic alcohol abuse. Volunteers are only eligible if they pass all in- and exclusion criteria.

One day before first PfSPZ Challenge Product administration (I-1)

In- and exclusion criteria will be reviewed, updates to the medical history will be recorded and all volunteers will be examined clinically. Any new medical issues or symptoms that have arisen since screening will be assessed. As for the other visits, sufficient time will be given to pose questions about the study, the vaccine and all other matters that may have arisen.

Additional analyses include urine dipstick, β-HCG test in female volunteers, complete blood count and biochemistry. Day I-1 will be used as baseline for subsequent analyses and samples for parasite qPCR, immunology will be taken. One serum aliquot (5 ml) is stored in -80°C until the end of the trial to serve as a baseline sample if unexpected AEs, which require extended laboratory analyses, occur. For psychological assessment, Big Five Inventory- 10 will be used.

Day before second and third PfSPZ Challenge product administration (II-1, III-1)

The day before second and third immunization volunteers will be examined clinically and AEs reviewed. In female volunteers a β-HCG test will be done. Blood and urine will be sampled for hematology, biochemistry, parasite qPCR, immunology.

Day of PfSPZ Challenge product administration (I, II, III, CI, CII, CIII)

All volunteers will have physical examinations performed prior to injection of PfSPZ Challenge products.

After the first immunization a participation (medic-alert) ID card will be issued to each volunteer with information including antimalarial sensitivity of the malaria parasite strains in use over the study (immunization and CHMI), study physician contact details, a diary card and a request that the research team be contacted immediately in the event of illness/accident. Each subject will also be issued with a digital thermometer. If the subject does not have their own mobile telephone they will be issued one for the duration of the study, and counseled about the importance of keeping it switched on or checking the messages regularly. In addition, full contact details for each subject

will be verified, including home address, home and work land-line telephone numbers where available and next-of-kin address, electronic mail address and telephone numbers. Subjects must also provide the investigators with the name and 24-hour telephone number of two emergency contacts. At least one of these should be a close friend, relative, or housemate who lives nearby and will be kept informed of their whereabouts for the duration of the study.

Following DVI volunteers remain in clinic for approximately 30 minutes.

Day after administration of a PfSPZ product (I1, II1, III1, CI1, CII1, CIII1)

All volunteers will be reviewed in clinic +1 day following administration of PfSPZ Challenge products and AEs assessed. Physical observations will be performed and reactogenicity to PfSPZ Challenge product administration is assessed.

Days 2 to 21 after administration of a PfSPZ product for immunization (I2–21, II2–II21, III2–III21)

Volunteers will be contacted daily via telephone, SMS, or email until + 6 days after each immunization.

Between + 7-21 days after immunizations, investigators will review clinical symptoms, AEs of all volunteers. Blood sampling for parasitology will be performed +7-11 on a daily basis then every second day: at +13, +15, +17, +19 and +21 days post the second and third vaccination

One day before administration of a PfSPZ product for CHMI (CI-1, CII-1, CIII-1)

Before each CHMI, a detailed clinical and laboratory work-up will be done. It includes physical examination, AE review, urine dipstick, β-HCG (female volunteers), virology (HBsAg, HCV, HIV), hematology, biochemistry, parasite qPCR, immunology.

Days 6 to 21 or until treatment (T) following CHMI (CI6-21 or to T, CII6-21 or to T, CIII6-21 or to T)

Volunteers will be seen in the clinic once daily to collect AEs. Blood will be sampled to measure parasitemia.

Treatment (T)

When asexual blood stage parasites are detected in the blood, treatment is initiated. In addition, volunteers are physically examined, an AE review is completed and samples for hematology, biochemistry, thick blood smear, parasite qPCR, immunology are taken. In case that parasitemia is present, parasites are cryopreserved. First line drug for antimalarial treatment is atovaquone-proguanil. In case that no parasitemia is detectable until +28 days, volunteers will not be treated but receive the same clinical and laboratory panel as volunteers on the day of parasitemia. For psychological assessment, Big Five Inventory- 10 will be used.

Follow up after CHMI (CI28, CI56, CII28, CII56, CIII28, CIII56)

All follow up visits include a physical examination, AE review and laboratory investigations (hematology, biochemistry, parasite qPCR, immunology).

Late follow up after the last CHMI (CIII105)

Volunteers will be seen in the clinic. The visit includes a physical examination, discussing medical history and AE rewiev. For psychological assessment, Big Five Inventory- 10 will be used.

6.2. Observations

6.2.1. Blood sampling and laboratory tests

A series of blood samples will be drawn to perform different tests. Types of tests to be performed include molecular biology, biochemistry, hematology, immunology, and parasitology. Details of these tests are as follows:

Parasites

These tests are used to determine parasitemia and parasite phenotype (thick blood smear, and qPCR *in vitro* culture).

After the second and third PfSPZ Challenge (NF54) injection for immunization parasites will be sampled daily between +7 and +11 days following vaccination then every second day: at +13, +15, +17, +19 and aditionnaly +21 after the third vaccination. If parasitemia develops, daily visits (as supplementary visits) are done till PCR results are three negative on three consecutive days than the volunteer joins the regular scheduling. An additional sample as negative control for PCR will be taken -1 days before inoculation the first immunizations and before each CHMI. During the CHMI period samples will be also obtained on a daily basis from +6 days until +21 days.

After CHMI, the same sampling method will be used, with the addition of follow up visits at +28 and +56 days post CHMI.

Selected blood samples obtained between +6 and +28 days post CHMI will be cryopreserved for later *in vitro* culture of Pf blood-stages.

Turn over time for thick blood smear microscopy will be < 6 hours to ensure timely treatment in case of a positive result.

Safety laboratory

Laboratory safety studies include a complete blood count (CBC), liver enzymes (ALT, AST, GGT), bilirubin, alkaline phosphatase, LDH and creatinine, blood urea nitrogen (BUN). These measurements will be done -1 days before, and +28 days after PfSPZ Challenge product administration. On the first day of asexual blood stage parasitaemia samples will be taken and may repeated and extended at the discretion of the investigators if clinically necessary.

One baseline serum aliquot (5 ml), drawn at -1 days of first PfSPZ administration, is preserved in -80°C until the end of the trial to serve as a baseline sample if unexpected AEs, which require extended laboratory analyses occur. In case of breakthrough infection, CQ level measurements can be taken according to the decision of the investigators.

Immunology

Tests to be performed include early antigen detection patterns and determination of antibodies as well as B-cell responses to plasmodial asexual blood-stage and pre-erythrocytic antigens by antibody quantification, immunofluorescence assays, and inhibition of sporozoite-invasion of hepatocytes assay (ISI), single cell analysis of antibody producing cells and T-cell responses. To detect early immunological responses to infection, complex response patterns and antigen sensing cells will be typed. In addition, sera will be assessed by protein microarray for antibodies to an array of Pf proteins.

Cellular responses of the adaptive immune system (B-cell and T-cell) will be analyzed -1 day before, and +28 days after each PfSPZ administration Additional sampling will occur on selected timepoints after the third immunization (III1, III15).

6.2.2. Administration of PfSPZ Challenge products

PfSPZ Challenge products will be administered by DVI according to standard operating procedures. On the day of PfSPZ Challenge product administration, volunteers are clinically examined, interviewed, and an AE review is performed after each PfSPZ Challenge product injection.

6.2.3. Administration of CQ

A member of the clinical team will prescribe and supervise CQ administration. An additional member of the clinical team will verify dosing and correct administration. All volunteers will receive 10 mg/kg CQ on the days of immunizations, prior to injections.

Table 8A: Study procedures immunization

	Screening	I-1	I	I1	I2-I3	II-1	II	II1	II2-6	II7-11	II13	II15	II17	II19
Day of Study	-	-1	0	1	2,3	4	5	6	7-11	12-16	18	20	22	24
Clinic visit	X	X	X	X		X	X	X		X	X	X	X	X
Daily contact (via phone call/SMS/email)					X				X					
Full History	X													
Eligibility Criteria Review	X	X												
Chemoprophylaxis			X[2]				X[2]							
DVI of PfSPZ Challenge product			X				X							
Physical examination	X	X	X	X		X	X	X						
Quiz, ICF	X													
ECG, cardiac risk assessment	X													
Randomization		X												
ID card, diary and thermometer			X											
Urine and urine β-HCG		X[1]				X[1]								
HIV, HBsAg, HCV	X[1]													
CDT	X[1]													
AE review			X	X		X	X	X		X	X	X	X	X
Reactogenicity of PfSPZ			X X	X			X X	X						
**Blood sampling**	X	X								X	X	X	X	X
Blood count	X	X[1]				X[1]						X		
Biochemistry[3]	X	X[1]				X[1]						X		
Serum banking		X												
Thick blood smear										X	X	X	X	X
Parasite qPCR		X								X	X	X	X	X
Immunology		X				X								
Psychological assessment		X												

	III-1	III	III1	III2-6	III7-11	III13	III15	III17	III19	III21	III28	III56
Day of study	27	28	29	30-34	35-39	41	43	45	47	49	56	84
Clinic visit	X	X	X		X	X	X	X	X	X	X	X
Daily contact (via phone call/SMS/email)				X								
Full History												
Eligibility Criteria Review												
Chemoprophylaxis		X[2]										
DVI of PfSPZ Challenge product		X										
Physical Examination	X	X	X									
Quiz, ICF												
ECG, cardiac risk assessment												
Randomization												
ID card, diary and thermometer												
Urine and urine □ -HCG	X[1]											
HIV, HBsAg, HCV												
CDT												
AE review	X	X	X		X	X	X	X	X	X	X	
Reactogenicity of PfSPZ		X X	X									
Blood sampling	X		X		X	X	X	X	X	X	X	
Blood count	X[1]		X								X	
Biochemistry[3]	X[1]										X	
Serum banking												
Thick blood smear	X				X	X	X	X	X	X	X	
Parasite qPCR	X				X	X	X	X	X	X	X	
Immunology	X		X				X				X	
Psychological assessment												

Safety relevant procedures (red) may be made out of schedule if a member of the study team or the local safety monitor decides; 1: results of tests are required before PfSPZ Challenge product injection and antimalarial administration (I); 2: 10 mg/kg CQ 3: LDH, ALT, AST, GGT, creatinine, bilirubin, alkaline phosphatase; BUN

Table 8B: Study procedures for CHMI (C). CI is performed 10 weeks following III. CII CHMI is done 26 weeks after last immunization and CIII is administer 52 weeks after the third immunization using exactly the same procedures.

	CI-1<br>CII-1<br>CIII-1	CI<br>CII<br>CIII	CI1<br>CII1<br>CIII1	CI2-CI5<br>CII2-CII5<br>CIII2-CIII5	CI6-CI21<br>CII6-CII21<br>CIII6-CIII21	Malaria (T)	CI28<br>CII28<br>CIII28	CI56<br>CII56<br>CIII56	CIII105
Day of Study	97<br>209<br>391	98<br>210<br>392	99<br>211<br>393	100-103<br>212-215<br>394-397	104-119<br>216-231<br>398-413		126<br>238<br>420	154<br>266<br>448	497
Clinic visit	X	X	X		X	X	X	X	X
Daily contact (via phone call/SMS/email)				X					
Full History									X
Eligibility Criteria Review	X								
Treatment with antimalarial						X			
DVI of PfSPZ Challenge product		X							
Physical Examination	X	X	X			X	X		X
Quiz, ICF									
Randomization									
Urine and urine β-HCG	X[1]								
AE review		X	X	X	X	X	X	X	X
Reactogenicity of PfSPZ		X X	X						
**Blood sampling**	X				X	X	X	X	
Blood count	X[1]					X	X		
Biochemistry[3]	X[1]					X	X	X	
Thick blood smear					X	X	X		
Parasite qPCR	X				X	X	X	X	
Serum banking									
Immunology	X								
Psychological assessment						X			X

Safety relevant procedures (red) may be made out of schedule if a member of the study team or the local safety monitor decides;
1: results of tests are required before PfSPZ Challenge product injection and antimalarial administration (I); 2: 10 mg/kg CQ 3: LDH, ALT, AST, GGT, creatinine, bilirubin, alkaline phosphatase; BUN

6.2.4. Malaria Diagnosis

Blood for malaria diagnosis will be collected on +6-11, +13,+15,+17,+19+21 days of each immunization and +6-21 days after CHMI. When asexual blood stage parasitemia occurs and treatment is initiated, sampling will be continued until two thick blood smears (drawn at least 12 hours apart) are negative. In the case of a symptomatic subject, blood smears may be collected more frequently (up to 6-hourly), based upon the clinical judgment of the investigator. A thick film is considered positive if at least 2 parasites are seen in 0.5 µl of blood (4 parasites per µl) and if an additional microscopist confirms this finding. An experienced and qualified microscopist must do at least one of the readings. The procedure is detailed in a SOP of the study site. Treatment may be initiated upon parasite-detection by qPCR without microscopic confirmation. PCR-confirmed parasitemia is defined as positive qPCR reactions on three samplings at least 12 hours apart with at least one measurement above 100 parasites per mL using qPCR protocols and conditions as given in the respective SOP. During immunization only qPCR is used for parasite detection.

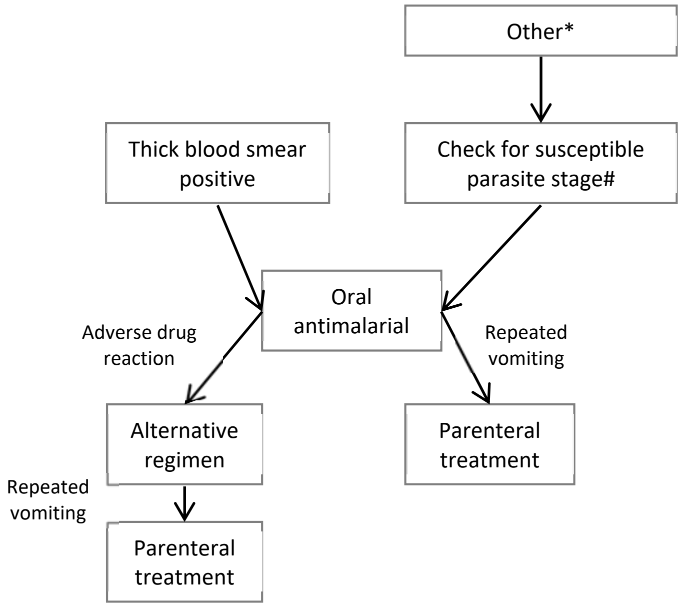

Figure 5: Malaria treatment decision tree. *E.g. volunteer decides to discontinue participation after PfSPZ Challenge product administration. #Evidence for asexual blood-stage (e.g. time from PfSPZ injection, PCR or thick blood smear). Parenteral treatment is given in an inpatient setting.

6.2.5. Antimalarial treatment

Confirmed *P. falciparum* infections will be treated promptly. Subjects who develop blood-stage *P.falciparum* infection following CHMI will be treated with a standard dose of a registered and effective antimalarial. In this trial atovaquone-proguanil is the first line treatment regimen. Both the NF54 strain and 7G8 clone of *P. falciparum* are highly sensitive to atovaquone-proguanil. When a case of malaria is diagnosed, each subject will have a clinical evaluation by one of the investigators (a physician) with appropriate history and physical examination where deemed to be necessary. Intake of the antimalarial will be directly observed and volunteers will remain in the clinic for at least 30 minutes after dosing. If vomiting of the drug occurs within 30 minutes, it may be re-dosed once. If necessary and according to a malaria treatment decision tree (Figure 5), they can be admitted to a ward of the internal medicine department for observation and parenteral treatment or other supportive measures if required. A subject will only be considered cured of *P. falciparum* infection when two consecutive blood smears, at least 12 hours apart are negative. The investigators may decide to treat any volunteer for malaria regardless of the thick film microscopy or qPCR

result if they are clinically concerned (and have discussed the case with the PI), or when a volunteer wishes to withdraw from the study. Antimalarial treatment will be given according to the following standards:

- Under observation
- Within 24 hours once a positive malaria diagnosis is made
- Based on recommendations of study physicians and Safety Monitoring Committee
- On request of volunteers or withdrawal of consent

Volunteers who remain undiagnosed with malaria at Day C+28 or +28 following CHMI are protected and will not be treated with an antimalarial. If a volunteer is unable to tolerate an oral antimalarial, the volunteer will be admitted forinpatient care and an appropriate parenteral antimalarial therapy will be prescribed. If a volunteer withdraws or is withdrawn from the study after receiving PfSPZ Challenge but before reaching the criterion for malaria diagnosis, a complete, appropriate, curative course of antimalarial therapy must be completed. Consequently, if a volunteer withdraws or is withdrawn from the study during CHMI, a course of antimalarial treatment will be given no sooner than 7 days after the last injection of PfSPZ Challenge, so as to begin after the expected liver-stage has ended. The importance of this will be emphasized to volunteers at screening and during the consent process. Volunteers who discontinue participation in the trial before CHMI do not need to be treated with an antimalarial. In case that another treatment other than atovaquone-proguanil has to be given after first CHMI during verification phase (e.g. due to intolerance), no second CHMI will be performed. Other treatment options following CHMI are those recommended by the German guidelines for the treatment of *P. falciparum* malaria. Chloroquine will not be used for treatment because 7G8 is chloroquine resistant. Prior to starting antimalarial treatment volunteers will be screened for drug interactions and contraindications to the respective drug(s). Volunteers will be reminded of the potential side effects of the antimalarial and receive a copy of the patient information sheet for their respective treatment regimen together with a schedule when their doses should be taken. Study personnel will directly observe intake of the drugs.

Criteria for hospital admission

Decisions regarding admission will be made by the investigators. Any non-malaria related symptoms will be treated according to international and German guidelines[79,82]. Treatment and hospital admission for severe malaria as well as non-improving uncomplicated malaria will be done according to international guidelines detailed in section 4.4.9.

Follow-up after malaria diagnosis

Volunteers will be reviewed in the clinic approximately 24, 48, and 72 hours after the first positive thick blood smear (and the start of antimalarial therapy). Volunteers will be examined in the clinic at least once a day for three days. Blood is sampled for blood film and qPCR daily and blood counts as well as blood chemistry are done at least on the day of first parasitemia and +28 days after inoculation. Additional blood samplings may be done after completion of the treatment or to follow-up any abnormal findings. If blood films taken at 48 and 72 hour post diagnosis are negative for parasites and the patient is asymptomatic or has mild, resolving symptoms, the volunteer will may not be seen again in clinic until +28 days post-CHMI. If not, the volunteer will continue to be reviewed in clinic daily until two consecutive negative blood films at least 12 hours apart following start of antimalarial treatment are negative, and all symptoms are mild or resolving. In case of continuing symptoms beyond the treatment period volunteers may be contacted by telephone, SMS, or electronic mail to document the end of any outstanding malaria symptoms on-going between parasitological cure and +28 days post CHMI.

Safety measures for the trial

Volunteer safety is of paramount importance. The following measures are in place to safeguard volunteer safety:

- Volunteers' understanding of the trial information will be tested by means of a questionnaire at screening. This provides further evidence that fully informed consent has been obtained.
- If the subject does not have their own mobile telephone they will be issued one for the duration of the study and counseled about the importance of keeping it switched on or checking the messages regularly.
- Before first immunization, full contact details for each subject will be documented, including home address, electronic mail address and mobile telephone numbers. Mobile telephone numbers will be verified prior to first vaccination to ensure the volunteers are easily contactable. Home and work landline telephone numbers where available will be documented and next of kin identified. At least two emergency contact numbers will be confirmed and verified as authentic for each subject who will participate in the trial. At least one of these emergency contacts should be a close friend, relative or housemate who lives nearby and will be kept informed of their whereabouts for the duration of the study.
- Prior to immunization and CHMI, the clinical study team will review subject adherence to the safety follow-up schedule to date. This review will attempt to identify any likelihood of unreliability on the part of the subject during the study. In the event that a subject shows a pattern of missed follow-up visits, the clinical team will discuss and emphasize the importance of compliance with all follow-ups to ensure safety throughout the trial period. Any subject expressing inability to comply with study requirements may be deemed unsuitable and will be excluded from further study-related interventions but will remain part of the safety cohort that continues to be followed-up for the study duration.
- On the day of first immunization volunteers will be provided with an identification card containing contact details for the study team, brief details of the study and the drug sensitivities of PfSPZ Challenge products.
- In the week after PfSPZ Challenge product administration either for immunization or for CHMI volunteers who do not have scheduled clinic visits will be contacted daily by telephone, email or SMS.
- Volunteers will be able to contact a medically qualified member of the study team 24 hours a day throughout the study period and will be instructed to contact the investigator immediately should they manifest any signs or symptoms they perceive as serious.
- If necessary, the study team will visit volunteers in their own homes if they are unable to attend the clinic for review.
- A member of the study team will directly observe administration of any antimalarial.
- Volunteers will be counseled that should they fail to return for treatment having been infected with Pf they could become very unwell and potentially die, even during the immunization phase. They will be instructed to remain in Tübingen and the immediate surrounding area for the duration of the intensive follow-up schedule (+21 days post PfSPZ Challenge product administrations)They will be informed that should they fail to attend a scheduled clinic visit post injection of PfSPZ Challenge products and they cannot be reached by telephone or email, their nominated contacts and next of kin will be informed, and a search started. If the volunteer cannot be identified appropriate authorities may be informed to help find the volunteer.

Measures to be taken if a volunteer goes missing post injection of PfSPZ Challenge products

In the unlikely event that a volunteer should (a) fail to show up for a scheduled clinical visit and (b) be unreachable by telephone or other means after being inoculated with PfSPZ Challenge products and before completion of an appropriate course of antimalarial therapy, the following will be informed;

- All investigators.
- The volunteer's nominated contact and next of kin.
- The trial Sponsor.
- The local safety monitor.
- The EC.
- The competent authority.

If this is unsuccessful the following may be informed;

- The local police department.
- Local accident and emergency departments.
- Local, national and international media.

All efforts will be made to locate the volunteer. While all parties will aim to preserve the volunteer's confidentiality, if necessary, details of the volunteer's identity and participation in the study may be passed to authorities and the media in order to help locate the missing individual. Volunteers will be informed of this possibility during screening and are only eligible when they give consent to communication of personal information to authorities and media in case they go missing.

7. Assessment of scientific objectives

The objectives of the study are to establish that an immunization regimen of three injections by DVI of 1.1x 10^5 PfSPZ of PfSPZ Challenge (NF54) and oral CQ chemoprophylaxis both administered on Days 0, 5, 28

1) is safe and well tolerated,
2) provides protection against repeat CHMI in healthy adult subjects 10, 26 and 52 weeks following the last immunization (Protective efficacy will be calculated based upon the proportion of volunteers infected after CHMI by injection of PfSPZ Challenge (7G8)).

Data will be collected on paper case report forms (CRF), which will be transcribed to an electronic data capture system. A qualified statistician will perform the analysis according to a pre-specified statistical analysis plan (SAP). A second, independent statistician, who is not involved in execution of the trial, will verify the statistical analysis plan and execution of data analysis.

8. Assessment of safety

Safety will be assessed through capture of adverse events (AE). The allocation ratio of 2:1 between immunized and placebo treated volunteers is used to maximize the amount of safety/tolerability data related to PfSPZ Challenge (NF54) with chemoprophylaxis. AE accrual starts one to three days before first PfSPZ Challenge (NF54) immunization (I-3 to I-1). AE (and as part of this adverse reactions [AR]) are graded according to a pre-defined scale (Annex 1 and 2), whereas a study physician does the grading of unexpected AEs. Clinical AEs are elicited by spontaneous reporting and a defined set of open questions to improve discriminative capacity. Lab abnormalities are reported as AE when outside the reference range. The clinical team may judge small deviations from the reference range as 'non-clinically significant'. Only clinically significant abnormalities are regarded as AE. A laboratory safety analysis is done -1 day before PfSPZ Challenge product

administartion, on the day of first parasitemia after CHMI for those who become positive, on +28 days post CHMI, and at anytime the clinical team decides that a laboratory analysis is required. A pre-defined list of reference intervals and severity grading of the most common laboratory measurements is attached (Annex 2). Verbatim-recorded AEs will be coded using the MedDRA terminology.

Documentation and Reporting of Adverse Events by the Investigator

All AEs will be documented in the CRF including the information listed below.

- Date of onset and resolution
- Severity
- Causal relationship with IMP / study treatment (antimalarial, PfSPZ Challenge product, malaria)
- Seriousness
- Interruption or withdrawal of study treatment and other measures taken

8.1. Definitions

Adverse Event (AE): An AE is any untoward medical occurrence in a volunteer, including a dosing error, which may occur during or after administration of PfSPZ Challenge products, during immunization or CHMI, and does not necessarily have to have a causal relationship with the intervention. An AE can therefore be any unfavorable and unintended sign (including an abnormal laboratory finding), symptom or disease temporally associated with the study intervention, whether or not considered related to the study intervention.

An AE may be:

- New symptoms/ medical conditions.
- New diagnosis.
- Changes of laboratory parameters.
- Diseases and accidents.
- Worsening of medical conditions/ diseases existing before clinical trial start.
- Recurrence of disease.
- Increase of frequency or intensity of episodical diseases.

A pre-existing disease or symptom will not be considered an AE unless there will be an untoward change in its intensity, frequency or quality. This change will be documented by the investigator. Surgical procedures themselves are not AEs; they are therapeutic measures for conditions that require surgery. The condition for which the surgery is required may be an AE. Planned surgical measures permitted by the clinical trial protocol and the condition(s) leading to these measures are not AEs, if the condition leading to the measure was present prior to inclusion into the trial. AEs are classified as "non-serious" or "serious".

Adverse Reaction (AR): An AR is any untoward or unintended response to PfSPZ Challenge products or any of the antimalarial chemotherapeutics (CQ) during immunization. This means that a causal relationship between PfSPZ Challenge product or the antimalarial treatment (given during immunization as part of the PfSPZ-CVac approach) and an AE is at least a reasonable possibility, i.e., the relationship cannot be ruled out. All cases judged by the sponsors as having a reasonable suspected causal relationship to PfSPZ Challenge products or antimalarial chemotherapy based on consistent clinical experience will qualify as adverse reactions.

Unexpected Adverse Reaction: An unexpected adverse reaction is where the nature, frequency or severity of the adverse reaction is inconsistent with that expected for the intervention as described in the protocol, investigator's brochure, other study documents or in consideration of the characteristics of the subject population being studied.

Serious Adverse Event (SAE): To ensure no confusion or misunderstanding of the difference between the terms 'serious' and 'severe', which are not synonymous, the following note of clarification is provided: The term 'severe' is often used to describe the intensity (severity) of a specific event (as in mild, moderate, or severe myocardial infarction); the event itself, however, may be of relatively minor medical significance (such as severe headache). This is not the same as 'serious,' which is based on patient/event outcome or action criteria usually associated with events that pose a threat to a volunteer's life or functioning. Seriousness (not severity) serves as a guide for defining regulatory reporting obligations.

An SAE is an AE that results in any of the following outcomes, whether or not considered related to the study intervention.

- Death (i.e., the AE caused or led to the fatality).
- Life-threatening event (i.e., the volunteer was, in the view of the investigator, at immediate risk of death from the event that occurred). This does not include an AE that, if it occurred in a more serious form, might have caused death.
- Persistent or significant disability or incapacity (i.e. substantial disruption of one's ability to carry out normal life functions).
- Hospitalization for at least 24 hours irrespective of relatedness to the AE. Admission to hospital or prolongation of hospitalization as a result of the AE. Hospitalization (including hospitalization for an elective procedure) for a pre-existing condition that has not worsened unexpectedly does not constitute a SAE. Hospitalization due to concerns about a subject's home circumstances does not constitute a SAE.
- An important medical event (that may not cause death, be life threatening, or require hospitalization) that may, based upon appropriate medical judgment, jeopardize the volunteer and/or require medical or surgical intervention to prevent one of the outcomes listed above. Examples of such medical events include allergic reaction requiring intensive treatment in an emergency room or clinic, blood dyscrasias, or convulsions that do not result in inpatient hospitalization.
- Congenital anomaly or birth defect.

Medical and scientific judgment should be exercised in deciding whether expedited reporting is appropriate in other situations, such as important medical events that may not be immediately life-threatening or result in death or hospitalization but may jeopardize the patient or may require intervention to prevent one of the other outcomes listed in the definition above. These should also usually be considered serious.

Serious Adverse Reaction (SAR): An adverse event (expected or unexpected) that is both serious and, in the opinion of the reporting investigator or sponsors, believed to be possibly, probably or definitely due to PfSPZ Challenge products, or an antimalarial used during immunization, based on the information provided.

Suspected Unexpected Serious Adverse Reactions (SUSARs): A SUSAR is a SAE that is unexpected and thought to be possibly, probably, or definitely related to PfSPZ Challenge products or an antimalarial used in the immunization phase of the study. SAEs have to be assessed by the Sponsors / CI whether they are both suspected, i.e. possibly related to IMP and 'unexpected', i.e. the nature and / or severity of which is not consistent with the applicable product information. They are then to be classified as Suspected Unexpected Serious Adverse Reactions (SUSARs).

In case, if either the investigator or the Sponsors / CI classify the SAE as 'suspected' i.e. either as related or probable, or possible, or unlikely-related to IMP and the SAE is unexpected as assessed by the Sponsors it will be categorized as a SUSAR.

All SUSARs are subject to an expedited reporting to the responsible ethics committee(s), the competent higher federal authority (i.e. either BfArM or PEI, and US FDA (through Sanaria)), Sanaria, and to all participating investigators.

Foreseeable Adverse Drug Reactions: PfSPZ Challenge products are expected to cause Pf clinical disease (malaria). The foreseeable AEs of clinical malaria include; fever, tachycardia, tachypnea, hypotension, feverishness, chills, rigor, sweats, headache, anorexia, nausea, vomiting, myalgia, arthralgia, chest wall pain, low back pain, fatigue, lymphopenia, and thrombocytopenia.

The foreseeable AEs following administration of the licensed medications CQ, A/P, artemether-lumefantrine, are listed in the Summary of product characteristics (SmPC) for these medications.

Foreseeable Serious Adverse Events: Volunteers developing clinical Pf disease may develop AEs that require in-patient admission and so would be deemed SAEs. All SAEs, foreseeable or not, will be notified to the Sponsor, who in turn will notify Sanaria. The Sponsor will notify the SMC as described below.

8.2. Causality assessment

For each AE, an assessment of the relationship of the AE to the study intervention(s) will be undertaken. The relationship of the adverse event with the study procedures will be categorized as unrelated, unlikely to be related, possibly related, probably related, or definitely related. An intervention-related AE refers to an AE for which there is a possible, probable or definite relationship to the study intervention. The investigator will use clinical judgment to determine the relationship. Alternative causes of the AE, such as the natural history of pre-existing medical conditions, concomitant therapy, other risk factors and the temporal relationship of the event to administration of PfSPZ Challenge products and antimalarials used during immunization phase will be considered and investigated.

8.3. Reporting procedures for AEs (Excluding SAEs)

All AEs occurring during the study observed by the investigator or reported by the patient will be recorded in the CRF. All AEs will be followed until they have abated, or until a stable situation has been reached. Depending on the event, follow up may require additional tests or medical procedures as indicated, and/or referral to the general physician or a medical specialist.

Clinically significant laboratory abnormalities will be followed up until they have normalized, or until an alternative explanation that is not related to the study has been provided.

AEs that result in a patient's withdrawal from the study or that are present at the end of the study will be followed up (if volunteer's consent to this) until a satisfactory resolution or stabilization occurs, or until a non-study related causality is assigned.

The severity of AEs will be assessed according to a scale with 4 grades (Table 9).

The investigator must document all adverse events that occur during the observation period set in this protocol. Additional instructions may be provided in the investigator file and in the case report form itself.

The following approach will be taken for documentation:

All adverse events (whether serious or non-serious) must be documented on the "adverse event" page of the case report form. If the adverse event is serious the investigator must complete, in addition to the "adverse event" page in the case report form, a "serious adverse event report form" at the time when the serious adverse event is detected. The investigator will document the date when he/she or any employee was first aware of the report and fax or email all SAE reports (initial and follow-up reports), even if they are incomplete, within one working day upon receipt to the safety department, the sponsor or its representative and to Sanaria:

Sponsor representative (Sponsor):

Prof. Dr. med. Peter G. Kremsner
Institut für Tropenmedizin
Wilhelmstraße 27, 72074 Tübingen
Telefon: +49 (0)7071/29-87179
Fax: +49 7071 29 5267
E-mail: peter.kremsner@uni-tuebingen.de

Safety department:

Prof. Dr. Benjamin Mordmüller
Institut für Tropenmedizin
Wilhelmstraße 27, 72074 Tübingen
Telefon: +49 (0)7071/29- 85446
Fax: +49 7071 29 4684
E-Mail: benjamin.mordmueller@uni-tuebingen.de

Prof. Dr. Michael Ramharter
Bernhard Nocht Institute für Tropenmedizin
Bernhard-Nocht-Straße 74
D-20359 Hamburg
Tel.: 040 4281 8511
E-Mail: ramharter@bnitm.de

Sanaria

Sanaria Inc.
9800 Medical Center Drive,
Rockville, MD 20850
United States of America
Tel: +1 301 7703222
SAE/SUSAR Fax: +1 240 3060596

Email: slhoffman@sanaria.com; trichie@sanaria.com; tmurshedkar@sanaria.com

The investigator should also assess the severity and the causal relationship between the event and the trial medication (PfSPZ Challenge product or antimalarial) or trial-related malaria. The investigator must determine also the relationship between the administration of IMP and the occurrence of an AE/SAE as defined below:

- Definitely Related: There is a certainty that the event was caused by IMP. The AE has a **strong temporal relationship** and an alternative cause is unlikely.
- Probably Related: There is a reasonable possibility that the event may have been caused by IMP. The AE has a **timely relationship** and **follows a known pattern of response**, but a potential alternative cause may be present.
- Possibly Related: There is a reasonable possibility that the event may have been caused by IMP. The AE has a **timely relationship** to the IMP; **however, the pattern of response is untypical**, and an alternative cause seems more likely or there is significant uncertainty about the cause of the event.
- Unlikely Related: Only a remote connection exists between the IMP and the reported AE. Other conditions including concurrent

illness, progression or expression of the disease state or reaction of the concomitant medication could explain the reported adverse event.

- Definitely not Related: An AE that does not follow a reasonable temporal sequence related to IMP and is likely to have been produced by the subject's clinical state, other modes of therapy or other known aetiology.

Table 9: Grading of Adverse Events.

**Grade 1**	Mild: Transient or mild discomfort (< 48 hours); no medical intervention/therapy required.
**Grade 2**	Moderate: Mild to moderate limitation in activity - some assistance may be needed; no or minimal medical intervention/therapy required.
**Grade 3**	Severe: Marked limitation in activity, some assistance usually required; medical intervention/therapy required, hospitalization possible.
**Grade 4**	Potentially life threatening

8.4. Reporting procedures for SAEs

In order to comply with current regulations on serious adverse event reporting to health authorities, the event will be documented accurately and notification deadlines respected as detailed in ICH-GCP and the German Medicines Law.

1. The PI or medical sub-investigator will notify the internal safety department, the Sponsor and Sanaria **within 24 hours** via email or phone from the time he learns about the event. Any SAE reports will be sent via fax or email to the Sponsor and to Sanaria.
2. Each SAE will be rated by the investigator for seriousness and relationship to IMP and study drug and by a Sponsor delegate giving a second rating and categorization, according to expectedness (SAE or SUSAR). Sanaria may also rate the SAE with regard to seriousness and relationship to IMP and study drug.
3. Sanaria will subsequently call or email the SMC **within 24 hours** for possibly, probably or definitely related SAEs after initial receipt of the information (or as soon as possible, if deadline cannot be met) to notify them of the event.
4. The PI or medical sub investigator will send a written report to the Sponsor and Sanaria **within 5 days via email or fax.**
5. The sponsor or its delegate will forward the PI's written SAE reports within the appropriate timelines to:
    a. The Paul-Ehrlich-Institute (PEI).
    b. The EC.
6. Sanaria will forward the PI's written SAE reports within the appropriate timelines to:
    a. The United States Food and Drug Administration (US FDA).
    b. The SMC. The SMC may request a meeting to discuss the SAE if needed.
7. The Sponsor or its delegate will forward any subsequent communication **from** PEI to the PI and Sanaria. Sanaria will forward any subsequent communication **from** US FDA to the PI and the sponsor. The sponsor or its delegate will then be responsible for forwarding documents from PEI or the US FDA to the EC, if required.
8. The same responsibilities outlined above applicable to SAEs will be applicable to the reporting of SUSARs.
9. Sanaria will forward any pertinent safety signals to other clinical centers studying PfSPZ products, in cases where the safety of volunteers in those trials could be affected.

All SAEs will be reported in the annual Development Safety Update Report.

Related SAEs will be reported to the EC by the PI or subinvestigators within 7 days via email or phone or fax.. In addition, the Sponsor will report any SAEs relating to licensed products used in the trial (e.g. CQ A/P, artemether-lumefantrine) to the relevant manufacturer.

The PI will provide Sanaria with study udpates and safety data on an ongoing basis, at least once per year, to enable Sanaria to comply with its reporting obligations. SAEs will be reported by Sanaria in accordance to the reporting requirements of the US FDA.

For contact details of Sponsor and Sanaria see chapter 8.3, for SMC see chapter 8.9.

8.4.1. Sponsors Assessment of the SAEs

All SAE will be subject to a second assessment as outlined above by Sanaria and by the trial Sponsor, or its delegate, which will be documented for each SAE in a 'Second Assessment Form'. The 'Second Assessment Form' will contain the following information:

- Assessment of relationship between SAE and IMP.
- Assessment of expectedness of SAE.
- Statement if the benefit / risk assessment for the trial did change as a result of SAE.

The responsibilities of second assessment will be further defined in an SAE manual, before study initiation.

8.4.2. Follow-up of Initial Report

All patients who have SAEs, whether considered associated with the use of the investigational products or not, must be monitored to determine the outcome. The clinical course of the SAE will be followed up according to accepted standards of medical practice even after the end of the period of observation, until a satisfactory explanation is found or the investigator considers it medically justifiable to terminate follow-up. Should the adverse event result in death, a full pathologist's report should be supplied, if possible.

The Sponsor will identify missing information for each SAE report and will require follow up information in regular intervals from the investigators until all queries are resolved or no further information can be reasonably expected. All responses to queries and supply of additional information by the investigator should follow the same reporting route and timelines as the initial report.

8.5. Reporting procedures for SUSARs

The Sponsor or its delegate will report all SUSARs according to national legislation to the competent authorities and the EC within 15 days or, if the SUSAR was life threatening or fatal, within 7 days. Authorities to be informed by the Sponsor or Sponsor's delegate are PEI and, via Sanaria, the US FDA. Sanaria will also inform all investigators of other Sanaria product related trials of relevant information about SUSARs that could adversely affect the safety of participants. In addition, the Sponsor will report any SUSARs relating to licensed products used in the the trial (e.g. CQ, A/P) to the relevant manufacturer.

For all deaths, available autopsy reports and relevant medical reports will be made available for reporting to the relevant authorities.

In addition to the reporting of SUSARs, the PI will submit a report at the end of the study (end of study report) to the EC, the Sponsor and Sanaria. The Sponsor will submit the report to the PEI and the responsible ethics committee. Sanaria will submit the report to the US FDA and SMC as required. Reporting to PEI and the ethics committee may be delegated from the Sponsor to the PI.

The following SAEs may not be considered as SUSAR:

1. Related to the following constitutional symptoms of malaria:
    a. fever
    b. headache
    c. diarrhea
    d. anorexia
    e. weakness
    f. myalgia
    g. arthralgia
    h. nausea
    i. vomiting
    j. chills/rigors

8.5.1. Report to the Investigator

Sanaria will inform investigators of all related SAEs or SUSARs occurring in other related trials including all relevant further information within the periods set by the authority. If new information becomes known that is different from the scientific information given to the investigator, all investigators will be informed of this by Sanaria.

Report to the Product Licence Holder

The Sponsor /CI will also inform the marketing authorization holder about all SUSARs including information reported to the competent supreme authority and ethics committee in accordance with contractual agreements.

8.5.2. Reporting to Safety Monitoring Committee

The SMC will be informed of all safety-relevant events by Sanaria. The SMC also has to assess whether any stopping rule as defined per protocol is reached or reasons for premature termination of the clinical trial exist. Based on its review the SMC will provide Sanaria and the Sponsor with recommendations regarding trial modification, continuation or termination.

8.5.3. Pharmakovigillance

Pharmakovigillance procedures and person (s) in charge will be defined in an SAE manual according to local SOPs, before study initiation.

8.6. Annual Safety Report

Once per year, the Trial Sponsor will supply a report on the safety of trial subjects with all available relevant information concerning patient safety during the reference period to the competent authorities. This report will also be supplied to the responsible ethics committee and Sanaria.

The annual safety report will be compiled according to the corresponding ICH guideline E2F "Development Safety Update Report – DSUR" and will include any modifications needed to make it suitable for complying with US FDA requirements.

The data lock point for the patient data to be included and analyzed is the day of the approval of the clinical trial by the Ethics committee.

The Trial Sponsor will supply the report within 60 days of one year after the reference date (data-lock point).

8.7. Unblinding of the Medical Product

If it is medically imperative to know which trial medication the subject is receiving, the investigator or authorized person should open the randomization envelope. The investigator or the person who breaks the blind must record the date and the reasons for doing so in the CRF, in the subject's

medical record and on the randomization envelope. Whenever possible, the Sponsor and Sanaria should be contacted before the blind is broken.

8.8. Procedures to be followed in the event of abnormal findings

Abnormal clinical findings from medical history, examination or blood tests will be assessed as to their clinical significance using the table in Appendix 1. If a test is deemed clinically significant, it may be repeated to ensure it is not a single occurrence. If a test remains clinically significant, the volunteer will be informed and appropriate medical care arranged as appropriate with the permission of the volunteer. Decisions to exclude the volunteer from enrolling in the trial or to withdraw a volunteer from the trial will be at the discretion of the Investigator.

8.9. Safety monitoring committee and local safety monitor

A Safety Monitoring Committee (SMC) will be convened to provide real-time safety oversight. The Sponsor will delegate Sanaria to establish and charter the SMC and coordinate the submission of reports, documents and requests to the SMC, as well as the dissemination of requests and recommendations from the SMC back to the principal investigator and Sponsor. The SMC will review all possibly, probably or definitely related SAEs. The SMC will be notified within 24 hours of Sanaria being aware of their occurrence via fax, email or telephone, (or as soon as possible if that deadline cannot be met), with receipt being acknowledged by email or fax. The SMC has the power to recommend termination of the study to Sanaria and to the Sponsor, if deemed necessary following a study intervention-related SAE.

There will be a minimum of two appropriately qualified committee members. Sanaria may convene the SMC *ad hoc* and will inform the PI and the Sponsor of all communications between Sanaria and the SMC.

The local safety monitor will be an experienced clinician qualified to evaluate safety data from clinical studies with malaria infections. He/she will be independent of the investigator team. The local safety monitor will serve as a voting member of the SMC.

8.10. The specific responsibilities of the SMC will be defined in a study-specific SMC Charter, together with the names and contact details of the SMC members before the study is initiated. Holding and stopping rules

The study may be placed on safety hold for the following reasons:

- On advice of the safety monitor.
- On advice of the investigators.
- On advice of the EC or the safety monitoring committee (SMC).
- One or more participants experience an unexpected serious adverse event (SAE) that is determined to be related to study product administration.
- One or more participants experience an unexpected SAE that is determined to be definitely related to administration of an antimalarial.
- Two or more of the same grade 3 or 4 adverse events in the same group of subjects which are unexpected and definitely related to the study product administration.

If the study is placed on safety hold it may only be restarted following agreement between EC, SMC, Sanaria, Sponsor, and PI about the conditions and need to continue the study.

The study will be stopped for the following reason:

- One or more participants experience a Suspected Unexpected Serious Adverse Reaction (SUSAR) that is related to PfSPZ Challenge (NF54) or an antimalarial used during immunization in the study.

A stopped study can only be re-initiated following approval by the regulatory authorities. In addition, it is required that PI, the Trial Sponsor and EC reach documented consensus about the conditions and need to continue the study.

8.11. Examination and Report of Changes in the Risk to Benefit Ratio

Without delay, and at the latest within 15 days of the decision for the need to do so, Sponsor will inform the PI (and the PI inform the Sponsor, the competent authority and the EC) of any events or factors that could result in a review of the risk-benefit ratio of the IMP.

These consist of especially:

- Individual reports of expected SAEs considered at least possibly related to IMP
- A clinically relevant increase in the rate of occurrence of at least possibly related AEs.
- SUSARs in trial subjects who have already completed the follow-up period of the clinical trial (”end-of-trial visit”).
- Factors emerging in connection with trial conduct or the development of the IMP that may affect the safety of persons concerned.

9. Statistics

Safety and tolerability data are presented as descriptive analysis, as listing and graphically. No formal hypothesis testing is done. Protective efficacy is calculated by comparison of proportions between immunized and placebo-treated volunteers using an unconditional exact test (Boschloo’s test) and time-to-parasitemia using a log-rank test. The level of significance is set at a two-tailed type 1 error alpha <5%. Statistical testing is done hierarchically: 1) protection against first CHMI, 2) protection against second CHMI, 3) protection against third CHMI. This means that in case that no significant protection against first CHMI is observed, formal testing of the of second and third CHMI will not be done. It also means that the protection against the first CHMI is the primary efficacy outcome variable.

Safety and tolerability data will be continuously monitored and the investigators will remain blinded on allocation to PfSPZ Challenge (NF54) immunization at least until + 28 days following third CHMI. The study is exploratory, nevertheless power calculations on the efficacy endpoint will allow detection of protective efficacy >75% (see below), the current target of efficacy set by the WHO malaria vaccine technology roadmap.

Interim analyses are planned after the first CHMI to asses efficacy, investigators remaining blinded to individuals. Reports on safety and tolerability data will be generated upon request from the SMC for information. Unless unblinding is required (Section8.7.), all reports will present un-blinded data, only.

Based on previous trials (TÜCHMI-001, TÜCHMI-002, PAMVAC, DSM265, MAVACHE, MALACHITE) recruiting a similar target population, it is expected that the amount of missing data will be minimal (<1%). In in case that missing data is present in endpoint relevant variables, sensitivity analyses using the last observation carried forward (LOCF) method will be done. Based on blind review of the data, alternative methods of imputation may be used as specified in the statistical analysis plan, which may be updated until locking of database and un-blinding of allocation.

9.1. Sample size

The objective of this study is to assess safety, tolerability and protective efficacy upon CHMI of the PfSPZ-CVac approach with three doses of PfSPZ Challenge (NF54) and CQ chemoprophylaxis. Safety and tolerability data are presented as descriptive analysis, as listing and graphically. No formal hypothesis testing is done. Placebo is used to control infectivity of PfSPZ

Challenge (7G8) following PfSPZ-CVac with CQ chemoprophylaxis. As of 1 July 2018, 100% of 85 malaria naïve volunteers became infected by DVI of 3200 PfSPZ Challenge (NF54), and 21/22 volunteers became infected by DVI of 3200 PfSPZ Challenge (7G8). Hence, the lower border of the 95% confidence interval of infectivity is 95%. To be able to show, with a power of 80% and a two-tailed alpha of 5%, that 25% or less of immunized volunteers and 95% of controls, allocated in a 2:1 ratio become infected, 14 immunized and 7 placebo-treated volunteers per group are required.

The sample size was calculated using the function nBinomial of package gsDesign of R version 2.15.1, which estimates the sample size required to detect a difference between two rates[86].

9.2. Study population

9.2.1. Modified intention to treat population (mITT)

The intention to treat population (ITT) is defined as all randomized volunteers. In CVac-Tü3 a modified ITT (mITT), which includes all volunteers who received at least one PfSPZ Challenge (NF54) injection or CQ or placebo will be used. Analysis of the mITT population will be done by allocation to the intervention.

All safety analyses will use the mITT population.

9.2.2. According to protocol population (ATP)

All volunteers who received three immunizations and at least one CHMI. Efficacy will be calculated using the according to protocol population (ATP). Sensitivity analysis will be done using the mITT population and assuming that all volunteers not receiving CHMI develop malaria (worst-case-scenario). This will be the primary analysis.

9.3. Randomization

14 volunteer will be randomly allocated to receive immunizations with PfSPZ and 7 volunteers will receive placebo (placebo:PfSPZ Challenge – 1:2).

A third party outside the study team will generate and distribute the randomization list.

A dedicated member of the formulation team, who is not involved in volunteer management or diagnostic activities, will keep the randomization envelopes and dosing schedule.

9.4. Allocation concealment

Volunteers will be allocated to placebo or PfSPZ Challenge (NF54) one day before the first PfSPZ inoculation. Adherence to the allocation sequence is ensured by provision of sequentially numbered sealed envelopes with the allocated intervention for each volunteer. After breaking the seal, volunteer ID, PfSPZ Challenge (NF54) dose, date and time are noted on the allocation form. Formulator and on-site pharmacist are responsible for provision of the correct product and sign and date the allocation form.

Only the PfSPZ formulation team will be aware of allocation to PfSPZ Challenge (NF54) or placebo for PfSPZ-CVac. No individual of the PfSPZ formulation team will be involved in clinical or diagnostic activities. Syringes will be labeled with the volunteer ID (VID), date and number of injection, but not with a label that indicates its content. Delivery of syringes from the PfSPZ preparation team to the clinical team will be done via a shielded box. PfSPZ Challenge (NF54) and placebo are both clear fluids and not distinguishable by appearance. One member of the formulation team transports the syringe and associated documentation (which does not include information about the allocation) to the clinical team with the volunteer who reside in a separate room.

9.5. Statistical methods

9.5.1. Demographics

A study flow chart (CONSORT flow chart) will be used to present number of volunteers screened, randomized, immunized and challenged.

Baseline characteristics (age, gender, height, weight, laboratory variables) will be tabulated.

Categorical variables will be presented as count and percentage. Numerical variables will be summarized as mean and standard deviation or median and interquartile range, as well as minimum and maximum.

9.5.2. Safety

Adverse events (AE) will be recorded from the screening visit until the last visit (CIII56) of the trial. Safety data of ITT population will be presented. Adverse events will be tabulated and presented as a heat map of at least possibly related adverse events over time and color to code for the maximal AE grade present at the time. Verbatim-recorded AEs will be coded using the MedDRA terminology and the proportion of subjects with grade 3 or 4 AE and SAE classified by MedDRA preferred term level, will be tabulated.

9.5.3. Grading and causality assessment

AE grading – AE are graded as Grade 1, 2 or 3. Where applicable, using predefined but non-binding grading schemes (laboratory abnormalities and solicited AE).

Relationship to intervention – The clinical team using five pre-defined levels to assign causality to the study interventions: unrelated, unlikely, possible, probable and definite relationship to the intervention.

9.5.4. Efficacy

Efficacy will be calculated using the efficacy ATP population after 1st CHMI, 2nd CHMI and 3rd CHMI, using a hierarchical testing procedure. Successful infection is defined as detection of asexual blood stage parasites within +28 days following CHMI, irrespective of symptoms. If no parasitemia occurs the volunteer is considered protected. The proportion of protected volunteers against first, second and third CHMI with PfSPZ Challenge (7G8) to immunized versus placebo treated volunteers will be used for primary efficacy analysis. Statistical testing will be done using Boschloo's test, an unconditional exact test. Vaccine efficacy will be expressed as 1 – relative risk (RR).

As exploratory analysis, time to parasitemia will be analysed using Kaplan-Meier plots and a log-rank test.

Further correlation and regression analysis (e.g. Cox regression) will be used for the analysis of exploratory endpoints.

9.6. Conduct of analyses

9.6.1. Blinding

In CVac-Tü3 neither the investigators (clinical and laboratory) nor the volunteers are aware of treatment allocation (double-blind). Data monitors and staff at the clinical site and the sponsor, except for the formulation team, are unaware of treatment allocation. The data capture system will also not contain any information about treatment allocation. Statistical analyses on primary and secondary endpoints will be coded before the blind is lifted using random group assignments. After the blind is lifted, any changes or amendments to this SAP are no longer permissible.

All analyses will be coded using R and SAS.

9.6.2. Missing values

The study team will undertake all effort to avoid missing data or blanks in the CRFs. Reasonable imputation methods will be used where necessary for complete case analysis. All methods for handling missing data will be specified and recorded in detail during blind review of the data. Sensitivity analyses may help to justify the choice of the particular method applied. In case of a drop out of the trial, full reporting of all reasons for discontinuation will be given where possible.

9.6.3. Immunological analyses

All immunological analyses will be considered exploratory. For core immunological assays using samples from placebo and PfSPZ Challenge (NF54)-immunized volunteers, laboratory investigators will be blinded to group allocation.

10. Quality control and quality assurance procedures

10.1. Investigator procedures

Approved site-specific and study-specific SOPs will be used at the trial site.

10.2. Monitoring

Monitoring will be performed according to ICH Good Clinical Practice (GCP) by an external monitor. Following a separate monitoring plan, the monitor will verify that the clinical trial is conducted and data are generated, documented and reported in compliance with the protocol, GCP and applicable regulatory requirements. The clinical site will provide direct access to all trial related source data/documents and reports for the purpose of monitoring and auditing by the Sponsor and inspection by local and regulatory authorities.

The monitoring activities will include at least , a pre-study or study initiation visit, regular monitor visits during the course of the trial, as well as a close out visit. Monitoring will end with the last visit after full documentation of the last patient enrolled (close out visit).

The aims of the monitoring visits are as follows:

- To check the informed consent documents
- To monitor subject safety (occurrence and documentation/reporting of Adverse Events (AEs) and Serious AEs).
- To check the completeness and accuracy of entries on the CRFs.
- To validate the entries on the CRFs against those in the source documents (source data verification (SDV).
- To evaluate the progress of the trial.
- To evaluate compliance with the trial protocol.
- To assess whether the trial is being performed according to GCP at the trial site.
- To discuss with the investigator aspects of trial conduct and any deficiencies found.
- A monitoring visit report is prepared for each visit describing the progress of the clinical trial and any problems.
- Informed Consent
- Inclusion/exclusion criteria (also for screening failure)
- Visit dates
- Dosing details and administration of chemoprophylactic regimen, CHMI and antimalarial treatment
- Primary stopping

- Volunteer status
- AEs
- Major protocol deviations

SDV will include 100% for

- Informed Consent
- Inclusion/exclusion criteria (also for screening failure)
- Visit dates
- Dosing details and administration of chemoprophylactic regimen, CHMI and antimalarial treatment
- Primary stopping
- Volunteer status
- AEs
- Major protocol deviations

and at least 20% of other source documents.

Reports will be provided by the monitor after each monitoring visit with receiption being acknowledged by the Sponsor via signature. The study site will endeavor to rectify any findings by the monitor before the next monitoring visit, or within a timeframe deemed appropriate by the monitor.

Once the monitoring plan (including visit schedule) has been approved and agreed upon by the monitor and the Sponsor it will be filed in the Trial Master File along with a confidentiality agreement which must be signed in advance of the initiation visit.

10.3. Modification to protocol

No amendments to this protocol will be made without consultation with, and agreement of, the Sponsor. Any amendments to the trial that appear necessary during the course of the trial must be discussed by the Investigator and the Sponsor concurrently. If agreement is reached concerning the need for an amendment, it will be prepared in writing by the Principal Investigator and will be made a formal part of the protocol following ethical and regulatory approval.

The Investigator is responsible for ensuring that any major changes to an approved trial, during the period for which ethical approval has already been given, are not initiated without EC review and approval, except to eliminate apparent immediate safety hazards to the volunteer.

10.4. Protocol deviation

Any deviations from the protocol will be documented in a protocol deviation form, which is filed in the trial master file and respective information is entered in the electronic database. Major deviations will be reported to EC within the required timeline. Minor deviations will be collated in an ongoing log and be submitted to the EC at the time of any required annual or end-of-study reports.

10.5. Audits

The project and quality manager at the trial site will conduct internal audits to check that the trial is being conducted, and data are being recorded, analyzed and accurately reported according to the protocol, trial SOPs and in compliance with ICH-GCP. The audits will also include laboratory activities according to an agreed audit schedule. The internal audits will supplement the internal monitoring process and will review processes not covered by the internal monitor.

The Sponsor may carry out an audit to ensure compliance with the protocol, GCP and appropriate regulations.

10.6. Trial progress

The progress of the trial will be overseen by the Principal Investigator.

11. Ethics

Notification of the authorities, approval and registration

The clinical trial will not be started before approval of the competent ethics committee (EC).

Before the start of the clinical trial, all necessary documentation will be submitted for regulatory approval to the competent supreme federal authority (Paul Ehrlich Institute, Paul-Ehrlich-Institut [PEI]). All planned substantial changes (see § 10, (1) of German GCP-Regulation) will be submitted for approval to the EC and PEI in writing as protocol amendments.

Before the trial starts, it will be registered in EUDRACT and in clinicaltrials.gov by the Sponsor.

11.1. Declaration of Helsinki

The Investigator will ensure that this study is conducted according to the principles of the current, 6th revision of the Declaration of Helsinki 2008.

11.2. ICH guidelines for good clinical practice

The Investigator and the Sponsor will ensure that this study is conducted in full conformity with relevant regulations and with the ICH guidelines for GCP (CPMP/ICH/135/95) July 1996 and the German Medicines Law..

11.3. Informed consent

Written, informed consent will be obtained, as described in section 6.2.

11.4. Research ethics committee

Ethical review will be done by the 'Ethikkommission der Medizinischen Fakultät und am Universitätsklinikum Tübingen'. The Principal Investigator will submit and, where necessary, obtain approval for all subsequent substantial amendments to the protocol and informed consent document.

11.5. Volunteer confidentiality

The data obtained in the course of the trial will be treated according to the Federal Data Protection Law (Bundesdatenschutzgesetz, BDSG).

All data will be pseudonymized. Volunteers receive a unique study number in the database and no names, addresses, initials, or other information that may lead to the identification of the subject will be stored in the database. Separate confidential files containing identifiable information will be stored in secured locations. Only the Sponsor, investigators, the monitor, and the PEI as the responsible authority will have access to the records. In addition to the informed consent, volunteers need to approve a separate data protection form to be eligible for participation.

11.6. Volunteer consent withdrawal

Volunteers may withdraw from the study at any time. Since malaria is a potentially life threatening disease if left untreated, measures are implemented to minimize the potential for any volunteers to stop their participation without receiving appropriate antimalarial treatment:

- Risk of complications due to malaria and the need to be treated even when study participation is stopped are presented in the volunteer information sheet ('Information für Studieninteressenten') and will be extensively discussed during screening.
- Volunteers are explicitly asked to consent on antimalarial treatment.
- Active knowledge about the necessity of treatment and possible complications is monitored with a quiz.

In the event that a volunteer withdraws consent and treatment cannot be given immediately, measures to locate and convince the volunteer to take an antimalarial treatment are undertaken by all legal means.

12. Data handling and record keeping

12.1. Data handling

The Principal Investigator or his designee will be the data manager with responsibility for delegating the receiving, entering, cleaning, querying, analyzing and storing all data that accrues from the study. All data will be entered in paper case record forms (CRFs) and transcribed into an electronic database by staff at the investigational site, following appropriate training. Double data entry will be performed. This includes safety, laboratory data and efficacy data.

12.2. Record keeping

All files and source documents will be kept confidentially in locked safety cabinets. Electronic data entry is done with a dedicated database system (OpenClinica) that fulfills regulatory requirements and follows Clinical Data Interchange Standards Consortium (CDISC) guidelines. OpenClinica is validated on a release basis, using a Quality System which checks for functionality as well as compliance to GCP and 21 CFR Part 11. The database will be custom developed for this study, enabling recruitment targets, monitoring access and study-specific events and CRFs to be defined. Requirements will also be set for monitoring and double data entry. The Principal Investigator and co-investigators will have access to records. The investigators will permit authorized representatives of the Sponsors, regulatory agencies and the monitor to examine (and when required by applicable law, to copy) clinical records for the purposes of quality assurance reviews, audits and evaluation of the study safety and progress. At the end of the study, all safety, laboratory data and efficacy data in the clinical database, as well as an audit trail will be transferred to the Trial Sponsor.

12.3. Use of samples, specimens and data

Samples, specimens and data collected under this protocol may be used to conduct protocol-related safety, parasitology and immunogenicity evaluations, exploratory laboratory evaluations related to the type of infection the vaccine was designed to prevent, exploratory laboratory evaluations related to vaccine research in general and for research assay validation. Genetic testing may be performed in accordance with the genetic testing information that was included in the study informed consent and data protection form.

12.4. Sample/ Data storage

All of the stored study research samples are labeled by a code that only the trial site can link to the subject. Samples are stored at the trial site in secure facilities with limited access. Data will be kept in password-protected computers. Only investigators or their designees will have access to the samples and data. According to the §13 of the German GCP-Ordinance all important trial documents (e.g. CRF) will be archived for at least 10 years after the trial termination.

In the case of withdrawal of consent the stored data collected to this time point will be stored and further used. Collected personal data will be stored in a pseudonymous manner for at least 10 years after the end of the trial if there are no other regulatory archiving periods.

The investigator(s) will archive all trial data (source data and Investigator Site File (ISF) including subject identification list and relevant correspondence) according to the section 4.9 of the ICH Consolidated Guideline on GCP (E6) and to local law or regulations.

Biological material will be stored for a maximum of 15 years unless a volunteer opts out. After 15 years samples are anonymized without the possibility to backtrack the identity of the volunteer. In case that a volunteer does not agree that samples are stored and used later on, samples are destroyed permanently.

12.5. Disposition of samples, specimens and data at completion of the protocol

In the future, other investigators (both at the trial site and outside) may wish to study these samples and/or data. The research use of stored, unlinked or de-identified samples will be exempt from the need for prospective ethical review and approval.

At the time of protocol termination, samples will remain in the trial site facility. De-identified (completely anonymized) samples may be transferred to another facility. Data will be archived in compliance with national and international guidelines.

12.6. Source data and case report forms (CRFs)

All protocol-required information will be collected in CRFs designed by the investigator or designee. All source documents will be filed with the CRFs. Source documents are original documents, data, and records from which the volunteer's CRF data are obtained. For this study, these will include, but are not limited to: volunteer consent form, data protection form, blood results, laboratory records and correspondence. In the majority of cases, CRF entries will be considered source data as long as the CRF is the site of the original recording (i.e. there is no other written or electronic record of data). In this study this will include, but is not limited to medical history, medication records, vital signs, physical examination records, urine assessments, blood results, ECG, adverse event data and details of study interventions. All source data and volunteer CRFs will be stored securely for at least 25 years.

12.7. Data protection

The study protocol, documentation, data and all other information generated will be held in strict confidence. No information concerning the study or the data will be released to any unauthorized third party, without prior written approval of the Sponsors. Volunteers are informed about the use of their data with a data protection form and approval by the volunteer is required for inclusion in the study.

12.8. Reporting

An interim statistical safety report (safety and efficacy data) will be prepared one month after the last patient visit. The interim report includes data from screening to Day CIII28 of the last immunized group. This report will also be forwarded to Sanaria and PEI. An end of study safety

report will be prepared by the PI and forwarded to the PEI and Sanaria who will be responsible for reporting to the US FDA as needed.

13. Financing and Insurance

13.1. Financing

The study will be funded by the Institute for Tropical Medicine in Tübingen.

13.2. Insurance

The trial will be covered by a clinical trial insurance from HDI-Gerling Industrie Versicherung and is per year for 50,000 euro per participant, to a total of 50,000,000 euro for the study. A copy of the insurance policy and conditions are distributed to the participant upon enrollment into the study and the patient is advised to adhere to the conditions of the insurance policy to safeguard a valid patient insurance.

13.3. Compensation of volunteers

Volunteers are compensated for the time spent in the clinical trial and associated travel costs according to the number and duration of visits in the study center or associated institutions. Short visits (e.g. daily visits following PfSPZ Challenge product administration) will be compensated with 25€. Visits above one hour (e.g. Screening) will be compensated with 50€. A volunteer who completes the study with all immunizations and CHMI will receive between 1200€ and 2600€ according to study visits.

13.4. Treatment costs

Treatment costs will be covered by project funds. Costs for hospitalization are covered either by project funds or the insurance (depending on the reason for hospital admission).

14. References

1. Okiro EA, Hay SI, Gikandi PW, et al. The decline in paediatric malaria admissions on the coast of Kenya. *Malar J.* 2007;6:151.
2. Murray CJ, Rosenfeld LC, Lim SS, et al. Global malaria mortality between 1980 and 2010: a systematic analysis. *Lancet.* 2012;379(9814):413-431.
3. World Health Organisation. Wold Malaria Report 2017. http://www.who.int/malaria/publications/world-malaria-report-2017/report/en/2. Published 2017. Accessed 06 June, 2018.
4. Sachs J, Malaney P. The economic and social burden of malaria. *Nature.* 2002;415(6872):680-685.
5. Tschan S, Kremsner PG, Mordmüller B. Emerging drugs for malaria. *Expert Opin Emerg Drugs.* 2012;17(3):319-333.
6. Feachem RGA, Phillips AA. Shrinking the malaria map: a prospectus on malaria elimination. 2009.
7. Vaccines mCGo. A research agenda for malaria eradication: vaccines. *PLoS Med.* 2011;8(1):e1000398.
8. Sergent E SE. Sur l'immunité dans le paludisme des Oiseaux. Conservation in vitro des sporozoites de Plasmodium relictum. Immunité relative obtenue par inoculation de ces sporozoite. In. Comptes rendus hebdomadaires des séances de l'Académie des sciences .1910:407-409.
9. Smith T, Ross A, Maire N, et al. Ensemble modeling of the likely public health impact of a pre-erythrocytic malaria vaccine. *PLoS Med.* 2012;9(1):e1001157.
10. Agnandji ST, Lell B, Soulanoudjingar SS, et al. First results of phase 3 trial of RTS,S/AS01 malaria vaccine in African children. *N Engl J Med.* 2011;365(20):1863-1875.
11. Agnandji ST, Lell B, Fernandes JF, et al. A phase 3 trial of RTS,S/AS01 malaria vaccine in African infants. *N Engl J Med.* 2012;367(24):2284-2295.
12. Genton B, Betuela I, Felger I, et al. A recombinant blood-stage malaria vaccine reduces Plasmodium falciparum density and exerts selective pressure on parasite populations in a phase 1-2b trial in Papua New Guinea. *J Infect Dis.* 2002;185(6):820-827.
13. Lusingu JP, Gesase S, Msham S, et al. Satisfactory safety and immunogenicity of MSP3 malaria vaccine candidate in Tanzanian children aged 12-24 months. *Malar J.* 2009;8:163.
14. Ogutu BR, Apollo OJ, McKinney D, et al. Blood stage malaria vaccine eliciting high antigen-specific antibody concentrations confers no protection to young children in Western Kenya. *PLoS One.* 2009;4(3):e4708.
15. Esen M, Kremsner PG, Schleucher R, et al. Safety and immunogenicity of GMZ2 - a MSP3-GLURP fusion protein malaria vaccine candidate. *Vaccine.* 2009;27(49):6862-6868.
16. Mordmüller B, Szywon K, Greutelaers B, et al. Safety and immunogenicity of the malaria vaccine candidate GMZ2 in malaria-exposed, adult individuals from Lambaréné, Gabon. *Vaccine.* 2010;28(41):6698-6703.
17. Bélard S, Issifou S, Hounkpatin AB, et al. A randomized controlled phase Ib trial of the malaria vaccine candidate GMZ2 in African children. *PLoS One.* 2011;6(7):e22525.
18. Thera MA, Doumbo OK, Coulibaly D, et al. A field trial to assess a blood-stage malaria vaccine. *N Engl J Med.* 2011;365(11):1004-1013.

19. Moorthy VS, Newman RD, Okwo-Bele JM. Malaria vaccine technology roadmap. *Lancet.* 2013;382(9906):1700-1701.
20. Russell PF, Mohan BN. THE IMMUNIZATION OF FOWLS AGAINST MOSQUITO-BORNE PLASMODIUM GALLINACEUM BY INJECTIONS OF SERUM AND OF INACTIVATED HOMOLOGOUS SPOROZOITES. *J Exp Med.* 1942;76(5):477-495.
21. Freund J, Sommer HE, Walter AW. Immunization against malaria: vaccination of ducks with killed parasites incorporated with adjuvants. *Science.* 1945;102(2643):200-202.
22. Nussenzweig RS, Nussenzweig V. Development of sporozoite vaccines. *Philos Trans R Soc Lond B Biol Sci.* 1984;307(1131):117-128.
23. Nussenzweig RS, Vanderberg J, Most H, Orton C. Protective immunity produced by the injection of x-irradiated sporozoites of plasmodium berghei. *Nature.* 1967;216(5111):160-162.
24. Clyde DF, Most H, McCarthy VC, Vanderberg JP. Immunization of man against sporozite-induced falciparum malaria. *Am J Med Sci.* 1973;266(3):169-177.
25. Rieckmann KH, Carson PE, Beaudoin RL, Cassells JS, Sell KW. Letter: Sporozoite induced immunity in man against an Ethiopian strain of Plasmodium falciparum. *Trans R Soc Trop Med Hyg.* 1974;68(3):258-259.
26. Hoffman SL, Goh LM, Luke TC, et al. Protection of humans against malaria by immunization with radiation-attenuated Plasmodium falciparum sporozoites. *J Infect Dis.* 2002;185(8):1155-1164.
27. Mueller AK, Labaied M, Kappe SH, Matuschewski K. Genetically modified Plasmodium parasites as a protective experimental malaria vaccine. *Nature.* 2005;433(7022):164-167.
28. Butler NS, Schmidt NW, Vaughan AM, Aly AS, Kappe SH, Harty JT. Superior antimalarial immunity after vaccination with late liver stage-arresting genetically attenuated parasites. *Cell Host Microbe.* 2011;9(6):451-462.
29. Annoura T, Ploemen IH, van Schaijk BC, et al. Assessing the adequacy of attenuation of genetically modified malaria parasite vaccine candidates. *Vaccine.* 2012;30(16):2662-2670.
30. Epstein JE, Tewari K, Lyke KE, et al. Live attenuated malaria vaccine designed to protect through hepatic $CD8^+$ T cell immunity. *Science.* 2011;334(6055):475-480.
31. Seder RA, Chang LJ, Enama ME, et al. Protection against malaria by intravenous immunization with a nonreplicating sporozoite vaccine. *Science.* 2013;341(6152):1359-1365.
32. Epstein JE, Paolino KM, Richie TL, et al. Protection against. *JCI Insight.* 2017;2(1):e89154.
33. Lyke KE, Ishizuka AS, Berry AA, et al. Attenuated PfSPZ Vaccine induces strain-transcending T cells and durable protection against heterologous controlled human malaria infection. *Proc Natl Acad Sci U S A.* 2017;114(10):2711-2716.
34. Sissoko MS, Healy SA, Katile A, et al. Safety and efficacy of PfSPZ Vaccine against Plasmodium falciparum via direct venous inoculation in healthy malaria-exposed adults in Mali: a randomised, double-blind phase 1 trial. *Lancet Infect Dis.* 2017;17(5):498-509.

35. Ishizuka AS, Lyke KE, DeZure A, et al. Protection against malaria at 1 year and immune correlates following PfSPZ vaccination. *Nat Med.* 2016;22(6):614-623.
36. Roestenberg M, McCall M, Hopman J, et al. Protection against a malaria challenge by sporozoite inoculation. *N Engl J Med.* 2009;361(5):468-477.
37. Roestenberg M, Teirlinck AC, McCall MB, et al. Long-term protection against malaria after experimental sporozoite inoculation: an open-label follow-up study. *Lancet.* 2011;377(9779):1770-1776.
38. Bijker EM, Bastiaens GJ, Teirlinck AC, et al. Protection against malaria after immunization by chloroquine prophylaxis and sporozoites is mediated by preerythrocytic immunity. *Proc Natl Acad Sci U S A.* 2013;110(19):7862-7867.
39. Beaudoin RL, Strome CP, Mitchell F, Tubergen TA. Plasmodium berghei: immunization of mice against the ANKA strain using the unaltered sporozoite as an antigen. *Exp Parasitol.* 1977;42(1):1-5.
40. Mordmüller B, Surat G, Lagler H, et al. Sterile protection against human malaria by chemoattenuated PfSPZ vaccine. *Nature.* 2017;542(7642):445-449.
41. Friesen J, Silvie O, Putrianti ED, Hafalla JC, Matuschewski K, Borrmann S. Natural immunization against malaria: causal prophylaxis with antibiotics. *Sci Transl Med.* 2010;2(40):40ra49.
42. Moxon R. Microbial Challenge Studies of Human Volunteers. *The Academy of Medical Sciences.* 2005.
43. Sauerwein RW, Roestenberg M, Moorthy VS. Experimental human challenge infections can accelerate clinical malaria vaccine development. *Nat Rev Immunol.* 2011;11(1):57-64.
44. Raju TN. The Nobel chronicles. 1927: Julius Wagner-Jauregg (1857-1940). *Lancet.* 1998;352(9141):1714.
45. FAIRLEY NH. Chemotherapeutic suppression and prophylaxis in malaria. *Trans R Soc Trop Med Hyg.* 1945;38(5):311-365.
46. FAIRLEY NH. Researches on paludrine (M.4888) in malaria; an experimental investigation undertaken by the L.H.Q. Medical Research Unit (A.I.F.) Cairns, Australia. *Trans R Soc Trop Med Hyg.* 1946;40(2):105-162.
47. Powell RD, McNamara JV. Infection with chloroquine-resistant Plasmodium falciparum in man: prepatent periods, incubation periods, and relationships between parasitemia and the onset of fever in nonimmune persons. *Ann N Y Acad Sci.* 1970;174(2):1027-1041.
48. Trager W, Jensen JB. Human malaria parasites in continuous culture. 1976. *J Parasitol.* 2005;91(3):484-486.
49. Ifediba T, Vanderberg JP. Complete in vitro maturation of Plasmodium falciparum gametocytes. *Nature.* 1981;294(5839):364-366.
50. Epstein JE, Rao S, Williams F, et al. Safety and clinical outcome of experimental challenge of human volunteers with Plasmodium falciparum-infected mosquitoes: an update. *J Infect Dis.* 2007;196(1):145-154.
51. Chulay JD, Schneider I, Cosgriff TM, et al. Malaria transmitted to humans by mosquitoes infected from cultured Plasmodium falciparum. *Am J Trop Med Hyg.* 1986;35(1):66-68.
52. Ballou WR, Hoffman SL, Sherwood JA, et al. Safety and efficacy of a recombinant DNA Plasmodium falciparum sporozoite vaccine. *Lancet.* 1987;1(8545):1277-1281.

53. Herrington DA, Clyde DF, Losonsky G, et al. Safety and immunogenicity in man of a synthetic peptide malaria vaccine against Plasmodium falciparum sporozoites. *Nature.* 1987;328(6127):257-259.
54. Moorthy VS, Diggs C, Ferro S, et al. Report of a consultation on the optimization of clinical challenge trials for evaluation of candidate blood stage malaria vaccines, 18-19 March 2009, Bethesda, MD, USA. *Vaccine.* 2009;27(42):5719-5725.
55. Sheehy SH, Spencer AJ, Douglas AD, et al. Optimising Controlled Human Malaria Infection Studies Using Cryopreserved P. falciparum Parasites Administered by Needle and Syringe. *PLoS One.* 2013;8(6):e65960.
56. Heppner DG, Gordon DM, Gross M, et al. Safety, immunogenicity, and efficacy of Plasmodium falciparum repeatless circumsporozoite protein vaccine encapsulated in liposomes. *J Infect Dis.* 1996;174(2):361-366.
57. Teirlinck AC, Roestenberg M, van de Vegte-Bolmer M, et al. NF135.C10: a new Plasmodium falciparum clone for controlled human malaria infections. *J Infect Dis.* 2013;207(4):656-660.
58. Verhage DF, Telgt DS, Bousema JT, et al. Clinical outcome of experimental human malaria induced by Plasmodium falciparum-infected mosquitoes. *Neth J Med.* 2005;63(2):52-58.
59. Rickman LS, Jones TR, Long GW, et al. Plasmodium falciparum-infected Anopheles stephensi inconsistently transmit malaria to humans. *Am J Trop Med Hyg.* 1990;43(5):441-445.
60. Roestenberg M, Bijker EM, Sim BK, et al. Controlled human malaria infections by intradermal injection of cryopreserved Plasmodium falciparum sporozoites. *Am J Trop Med Hyg.* 2013;88(1):5-13.
61. Roestenberg M, O'Hara GA, Duncan CJ, et al. Comparison of clinical and parasitological data from controlled human malaria infection trials. *PLoS One.* 2012;7(6):e38434.
62. Shekalaghe S, Rutaihwa M, Billingsley PF, et al. Controlled human malaria infection of Tanzanians by intradermal injection of aseptic, purified, cryopreserved Plasmodium falciparum sporozoites. *Am J Trop Med Hyg.* 2014;91(3):471-480.
63. Mordmüller B, Supan C, Sim KL, et al. Direct venous inoculation of Plasmodium falciparum sporozoites for controlled human malaria infection: a dose-finding trial in two centres. *Malar J.* 2015;14:117.
64. Gómez-Pérez GP, Legarda A, Muñoz J, et al. Controlled human malaria infection by intramuscular and direct venous inoculation of cryopreserved Plasmodium falciparum sporozoites in malaria-naïve volunteers: effect of injection volume and dose on infectivity rates. *Malar J.* 2015;14:306.
65. Hodgson SH, Juma E, Salim A, et al. Evaluating controlled human malaria infection in Kenyan adults with varying degrees of prior exposure to Plasmodium falciparum using sporozoites administered by intramuscular injection. *Front Microbiol.* 2014;5:686.
66. Lell B, Mordmüller B, Dejon Agobe JC, et al. Impact of Sickle Cell Trait and Naturally Acquired Immunity on Uncomplicated Malaria after Controlled Human Malaria Infection in Adults in Gabon. *Am J Trop Med Hyg.* 2018;98(2):508-515.
67. Sulyok M, Rückle T, Roth A, et al. DSM265 for Plasmodium falciparum chemoprophylaxis: a randomised, double blinded, phase 1 trial with controlled human malaria infection. *Lancet Infect Dis.* 2017;17(6):636-644.

68. Laurens MB, Duncan CJ, Epstein JE, et al. A consultation on the optimization of controlled human malaria infection by mosquito bite for evaluation of candidate malaria vaccines. *Vaccine.* 2012;30(36):5302-5304.
69. Epstein JE, Paolino KM, Richie TL, et al. Protection against Plasmodium falciparum malaria by PfSPZ Vaccine. *JCI Insight.* 2017;2(1):e89154.
70. Su XZ, Carucci DJ, Wellems TE. Plasmodium falciparum: parasite typing by using a multicopy microsatellite marker, PfRRM. *Exp Parasitol.* 1998;89(2):262-265.
71. Weber JL. Interspersed repetitive DNA from Plasmodium falciparum. *Mol Biochem Parasitol.* 1988;29(2-3):117-124.
72. de Bruin D, Lanzer M, Ravetch JV. The polymorphic subtelomeric regions of Plasmodium falciparum chromosomes contain arrays of repetitive sequence elements. *Proc Natl Acad Sci U S A.* 1994;91(2):619-623.
73. Moorthy, VS. Standardization of Design and Conduct of P. falciparum Sporozoite Challenge Trials. In:2011.
74. Church LW, Le TP, Bryan JP, et al. Clinical manifestations of Plasmodium falciparum malaria experimentally induced by mosquito challenge. *J Infect Dis.* 1997;175(4):915-920.
75. Hoffman SL, Billingsley PF, James E, et al. Development of a metabolically active, non-replicating sporozoite vaccine to prevent Plasmodium falciparum malaria. *Hum Vaccin.* 2010;6(1):97-106.
76. Egan JE, Hoffman SL, Haynes JD, et al. Humoral immune responses in volunteers immunized with irradiated Plasmodium falciparum sporozoites. *Am J Trop Med Hyg.* 1993;49(2):166-173.
77. Bastiaens GJ, van Meer MP, Scholzen A, et al. Safety, Immunogenicity, and Protective Efficacy of Intradermal Immunization with Aseptic, Purified, Cryopreserved Plasmodium falciparum Sporozoites in Volunteers Under Chloroquine Prophylaxis: A Randomized Controlled Trial. *Am J Trop Med Hyg.* 2016;94(3):663-673.
78. Bundesärztekammer, Paul-Ehrlich-Institut. Bekanntmachung der Richtlinien zur Gewinnung von Blut und Blutbestandteilen und zur Anwendung vonBlutprodukten (Hämotherapie) gemäß §§ 12 und 18 des Transfusionsgesetzes. In:2010.
79. (DTG)' DGfTuIG. S1-Leitlinie 042-001: Diagnostik und Therapie der Malaria . In. http://www.dtg.org/images/Leitlinien_DTG/Leitlinie_Malaria_2016.pdf2016.
80. Krishna S, White NJ. Pharmacokinetics of quinine, chloroquine and amodiaquine. Clinical implications. *Clin Pharmacokinet.* 1996;30(4):263-299.
81. Ducharme J, Farinotti R. Clinical pharmacokinetics and metabolism of chloroquine. Focus on recent advancements. *Clin Pharmacokinet.* 1996;31(4):257-274.
82. Organization WH. Management of severe malaria: a practical handbook. Geneva: World

Health Organization. In:2013.
83. Schneider P, Schoone G, Schallig H, et al. Quantification of Plasmodium falciparum gametocytes in differential stages of development by quantitative nucleic acid sequence-based amplification. *Mol Biochem Parasitol.* 2004;137(1):35-41.
84. Piepoli MF, Hoes AW, Agewall S, et al. 2016 European Guidelines on cardiovascular disease prevention in clinical practice: The Sixth Joint Task Force of the European Society of Cardiology and Other Societies on

Cardiovascular Disease Prevention in Clinical Practice (constituted by representatives of 10 societies and by invited experts): Developed with the special contribution of the European Association for Cardiovascular Prevention & Rehabilitation (EACPR). *Eur J Prev Cardiol.* 2016;23(11):NP1-NP96.

85. Deutschland B. Gesetz zur Verhütung und Bekämpfung von Infektionskrankheiten beim Menschen (Infektionsschutzgesetz - IfSG) [Internet]. 2001;Available from: http://www.gesetze-im-internet.de/ifsg/index.html In:2001.

86. Hermans L, Deblander A, De Keyser P, Scheys I, Lesaffre E, Westelinck KJ. At equipotent doses, isradipine is better tolerated than amlodipine in patients with mild-to-moderate hypertension: a double-blind, randomized, parallel-group study. *Br J Clin Pharmacol.* 1994;38(4):335-340.

Annex 1: Tables for clinical abnormalities

**Local Reaction to Injectable Product**	**Mild (Grade 1)**	**Moderate (Grade 2)**	**Severe (Grade 3)**	**Potentially Life Threatening (Grade 4)**
Pain	Does not interfere with activity	Repeated use of non-narcotic pain reliever >24 hours or interferes with activity	Any use of narcotic pain reliever or prevents daily activity	Hospitalization
Tenderness	Mild discomfort when touched	Discomfort while moving	Significant discomfort at rest	Hospitalization
[1]Erythema/Redness	2.5 – 5 cm	5.1 – 10 cm	>10 cm	Necrosis or exfoliative dermatitis
[2]Induration/Swelling	2.5 – 5 cm and does not interfere with activity	5.1 – 10 cm or interferes with activity	>10 cm or prevents daily activity	Necrosis

**[3] Vital Signs**	**Mild (Grade 1)**	**Moderate (Grade 2)**	**Severe (Grade 3)**	**Potentially Life Threatening (Grade 4)**
[4] Fever (°C)	38.0 – 38.4	38.5 – 38.9	39.0 – 40	>40
Tachycardia (beats per minute)	101 – 115	116 – 130	>130	Hospitalization for arrhythmia
[5] Bradycardia (beats per minute)	50 – 54	45 – 49	<45	Hospitalization for arrhythmia
Hypertension – systolic (mmHg) *	141 – 150	151 – 155	>155	Hospitalization for malignant hypertension
Hypertension – diastolic (mmHg)*	91 – 95	96 – 100	>100	Hospitalization for malignant hypertension
Hypotension – systolic (mmHg)	85 – 89	80 – 84	<80	Hospitalization for hypotensive shock
Respiratory Rate (breaths per minute)	17 – 20	21 – 25	>25	Intubation

1. In addition to grading the measured local reaction at the greatest single diameter, the measurement will be recorded as a continuous variable.
2. Induration/Swelling will be evaluated and graded using the functional scale as well as the actual measurement.
3. Subject should be at rest for all vital sign measurements.
4. Indicate way of measurement (tympanic and rectal are acceptable).
5. When resting heart rate is between 60 – 100 beats per minute. Use clinical judgment when characterizing bradycardia among some healthy subject populations, for example, conditioned athletes.

* Accidentally, in good health measured high values may interpreted as non clinically significant, and not reported as an AE

**Systemic (General)**	**Mild (Grade 1)**	**Moderate (Grade 2)**	**Severe (Grade 3)**	**Potentially Life Threatening (Grade 4)**
Nausea/vomiting	No interference with activity or 1–2 episodes/24 hours	Some interference with activity or >2 episodes/24 hours	Prevents daily activity, requires outpatient IV hydration	Hospitalization for hypotensive shock
Diarrhea	2–3 loose stools or <400 g/24 hours	4–5 stools or 400–800 g/24 hours	6 or more watery stools or >800 g/24 hours or requires outpatient IV hydration	Hospitalization
Headache	No interference with activity	Repeated use of non-narcotic pain reliever > 24 hours or some interference with activity	Significant; any use of narcotic pain reliever or prevents daily activity	Hospitalization
Fatigue	No interference with activity	Some interference with activity	Significant; prevents daily activity	Hospitalization
Myalgia	No interference with activity	Some interference with activity	Significant; prevents daily activity	Hospitalization

**Unsolicited Adverse Event**	**Mild (Grade 1)**	**Moderate (Grade 2)**	**Severe (Grade 3)**	**Potentially Life Threatening (Grade 4)**
Description of Adverse Event	No interference with activity	Some interference with activity not requiring medical intervention	Prevents daily activity and requires medical intervention	Hospitalization

Annex 2: Laboratory abnormalities

Laboratory reference intervals (RI) and toxicity scale (adapted from the FDA to the central laboratory of the UKT)

Variable	Unit	Gender	Ref. Range	Mild Grade 1	Moderate Grade 2	Severe Grade 3	Potentially Life Threatening Grade 4
Hemoglobin	g/dl	F<br>M	12.0, 16.0<br>14.0, 18.0	11.0-11.9<br>12.5-13.5	9.5-10.9<br>10.5-12.4	8.0-9.4<br>8.5-10.4	<8.0<br><8.5
Leucocyte	$10^3/\mu l$		4.0, 9.5	10.8-15.0*<br>2.5-3.5#	15.1-20.0<br>1.5-2.4	20.1-25.0<br>1.0-1.4	>25.0<br><1.0
Thrombocyte	$10^3/\mu l$		150, 450	125-140	100-124	25-99	<25
Neutrophils	$10^3/\mu l$		1.6, 7.6	1.3-1.5	1.0-1.2	0.5-0.9	<0.5
Eosinophils	$10^3/\mu l$		0.04, 0.38	0.6-1.5	1.6-5.0	>5	'hypereosinophilia'
Lymphocytes	$10^3/\mu l$		0.8, 4.3	0.65-0.79	0.5-0.64	0.25-0.49	<0.25
Creatinine	mg/dl	F<br>M	0.5, 0.8<br>0.6, 1.1	1.5-1.7	1.8-2.0	2.1-2.5	>2.5
ALT	U/l	F<br>M	<35<br><50	1.1-2.5§	2.6-5.0§	5.1-10§	>10§
AST	U/l	F<br>M	<34<br><50	1.1-2.5§	2.6-5.0§	5.1-10§	>10§
GGT	U/l	F<br>M	<40<br><60	1.1-2.5§	2.6-5.0§	5.1-10§	>10§
ALP	U/l	F<br>M	<105<br><130	1.1-2.5§	2.6-5.0§	5.1-10§	>10§
Protein	g/l		65, 85	55-60	50-54	<50	nd°
Albumin	g/l		34, 48	28-31	25-27	<25	nd°
Bilirubin (conjugated)	mg/dl		<1.1	1.1-1.5§	1.6-2.0§	2.0-3.0§	>3.0§
Glucose	mg/dl		70–110	65-69#<br>110-125*	55-64#<br>126-200*	45-54#<br>>200*	<45#<br>&
Sodium	mmol/l		135–143	132-134#<br>144-145*	130-131#<br>146-147*	125-129#<br>148-150*	<125#<br>>150*
Potassium	mmol/l		3.7–5.0	3.5-3.6#<br>5.1-5.2*	3.3-3.4#<br>5.3-5.4*	3.1-3.2#<br>5.5-5.6*	<3.1#<br>>5.6*

*: increase from RI; #: decrease from RI; §: times increase from upper norm value; °: reported as 'nicht definiert' (not defined); & Insulin requirements or hyperosmolar coma

Annex 3: Questionnaires for psychological assessment

Big Five Inventory (BFI), deutsche Version.[2]

Ich…	sehr unzutreffend	eher unzutreffend	weder zutreffend noch unzutreffend	eher zutreffend	sehr zutreffend
…bin eher zurückhaltend, reserviert	1	2	3	4	5
…schenke anderen leicht Vertrauen, glaube an das Gute im Menschen	1	2	3	4	5
…bin bequem, neige zur Faulheit.	1	2	3	4	5
…bin entspannt, lasse mich durch Stress nicht aus der Ruhe bringen.	1	2	3	4	5
…gehe aus mir heraus, bin gesellig	1	2	3	4	5
… neige dazu, andere zu kritisieren.	1	2	3	4	5
… habe nur wenig künstlerisches Interesse.	1	2	3	4	5
…erledige Aufgaben gründlich.	1	2	3	4	5
…werde leicht nervös und unsicher.	1	2	3	4	5
…habe eine aktive Vorstellungskraft, bin fantasievoll.	1	2	3	4	5

[2] Rammstedt B,. Kemper CJ, Klein MC, Beierlein C, Kovaleva A. Eine kurze Skala zur Messung der fünf Dimensionen der Persönlichkeit. methoden, daten, analysen · 2013, Jg. 7(2), S. 233-249.

Big Five Inventory-10 (BFI-10) English version.[3,4,5]

How well do the following statements describe your personality?

I see myself as someone who…	Disagree strongly	Disagree a little	Neither agree nor disagree	Agree a little	Agree strongly
…is reserved	1	2	3	4	5
…is generally trusting	1	2	3	4	5
…tends to be lazy	1	2	3	4	5
…is relaxed, handles stress well	1	2	3	4	5
…is outgoing, sociable	1	2	3	4	5
… tends to find fault with others	1	2	3	4	5
… has few artistic interests	1	2	3	4	5
…does a thorough job	1	2	3	4	5
…gets nervous easily	1	2	3	4	5
…has an active imagination	1	2	3	4	5

[3] John, O. P., Naumann, L. P., & Soto, C. J. (2008). Paradigm Shift to the Integrative Big-Five Trait Taxonomy: History, Measurement, and Conceptual Issues. In O. P. John, R. W. Robins, & L. A. Pervin (Eds.), Handbook of personality: Theory and research (pp. 114-158). New York, NY: Guilford Press.

[4] John, O. P., Donahue, E. M., & Kentle, R. L. (1991). The Big Five Inventory--Versions 4a and 54. Berkeley, CA: University of California,Berkeley, Institute of Personality and Social Research.

[5] Benet-Martinez, V., & John, O. P. (1998). *Los Cinco Grandes* across cultures and ethnic groups: Multitrait multimethod analyses of the Big Five in Spanish and English. Journal of Personality and Social Psychology,75, 729-750.

		and affecting all following chapters	changed and all references above number 33 were renumbered.
6.0	Treatment of *P.falciparum* infection	2.7.1. Potential risks	A dose definition of A/P treatment was inserted
6.0	Quantifciation of cardiac risk factors	5.4. Exclusion criteria	The range of this exclusion criteria was expanded from 5 to 10 years Quantification score was added to the reference list (#84).
6.0	Criteria for hospital admission	6.2.5. Antimalarial treatment	Decision about admission will be made by the investigator
6.0	Safety reporting procedures were refined for AEs, SAEs and SUSARs<br>SMC responsibility	8.4 Reporting procedures for SAEs<br>8.4.1. Sponsors Assessment of the SAEs<br>8.9. Safety monitoring committee and local safety monitor	5) Sanaria instead of Sponsor will be fully responsible for organizing and reporting to the SMC<br>6) Only related SAEs will be reported to the SMC<br>7) Timelines for reporting to the SMC were expanded
6.0	Statistical Analysis of Efficacy	9.5.4. Efficacy	Refined to control for TypeI error after second and third CHMI

Sponsor Representative Approval
Sponsor Representative: Peter G. Kremsner
Institut für Tropenmedizin

Signature **Date**

Protocol Version 6.0 – Investigator agreement

"I have read this protocol and its amendment and agree to abide by all provisions set forth therein. I agree to comply with the principles of the International Conference on Harmonization Tripartite Guideline on Good Clinical Practice."

Principal Investigator: Zita Sulyok
Institut für Tropenmedizin

Signature **Date**

Statistician: Benjamin Mordmüller
Institut für Tropenmedizin

Signature **Date**

Confidentiality Statement

This document contains confidential information that must not be disclosed to anyone other than the trial Sponsor, the Investigator Team, and members of the Institutional Review Board. This information cannot be used for any purpose other than the evaluation or conduct of the clinical investigation without the prior written consent of the Principal Investigator.
