# Supplementary material for: Heterologous protection against malaria by a simple chemoattenuated PfSPZ vaccine regimen in a randomized trial
Source: Nat Commun. 2021 May 4;12:2518. doi: 10.1038/s41467-021-22740-w (PMC8097064; doi:10.1038/s41467-021-22740-w)
Supplement: Supplementary file 2 — Reporting Summary [file 41467_2021_22740_MOESM2_ESM.pdf]

nature research

Corresponding author(s): Rolf Fendel

Last updated by author(s): Mar 12, 2021

Reporting Summary

Nature Research wishes to improve the reproducibility of the work that we publish. This form provides structure for consistency and transparency in reporting. For further information on Nature Research policies, see our Editorial Policies and the Editorial Policy Checklist.

Statistics

For all statistical analyses, confirm that the following items are present in the figure legend, table legend, main text, or Methods section.

n/a	Confirmed	
☐	☒	The exact sample size (n) for each experimental group/condition, given as a discrete number and unit of measurement
☐	☒	A statement on whether measurements were taken from distinct samples or whether the same sample was measured repeatedly
☐	☒	The statistical test(s) used AND whether they are one- or two-sided<br>*Only common tests should be described solely by name; describe more complex techniques in the Methods section.*
☐	☒	A description of all covariates tested
☐	☒	A description of any assumptions or corrections, such as tests of normality and adjustment for multiple comparisons
☐	☒	A full description of the statistical parameters including central tendency (e.g. means) or other basic estimates (e.g. regression coefficient) AND variation (e.g. standard deviation) or associated estimates of uncertainty (e.g. confidence intervals)
☐	☒	For null hypothesis testing, the test statistic (e.g. F, t, r) with confidence intervals, effect sizes, degrees of freedom and P value noted<br>*Give P values as exact values whenever suitable.*
☒	☐	For Bayesian analysis, information on the choice of priors and Markov chain Monte Carlo settings
☒	☐	For hierarchical and complex designs, identification of the appropriate level for tests and full reporting of outcomes
☐	☒	Estimates of effect sizes (e.g. Cohen's d, Pearson's r), indicating how they were calculated

Our web collection on statistics for biologists contains articles on many of the points above.

Software and code

Policy information about availability of computer code

Data collection	Microplate reader: CLARIOstar with Clariostar Software Version 5.40 R2 and MARS Data Analysis Software Version 3.31<br>Microarray software: ArrayCAM 400-S Microarray Imager Software V 2.2
Data analysis	Data was analysed using GraphPad Prism v9.0.2 and R v4.0.4.<br>R packages:<br>gsDesign v3.2<br>exact2x2 v1.6.5<br>PAA v3.12<br>LIMMA package v3.28.14<br>ggplot2 v3.3.3

For manuscripts utilizing custom algorithms or software that are central to the research but not yet described in published literature, software must be made available to editors and reviewers. We strongly encourage code deposition in a community repository (e.g. GitHub). See the Nature Research guidelines for submitting code & software for further information.

Data

Policy information about availability of data

All manuscripts must include a data availability statement. This statement should provide the following information, where applicable:

- Accession codes, unique identifiers, or web links for publicly available datasets
- A list of figures that have associated raw data
- A description of any restrictions on data availability

Individual participant data that underlie the results reported in this publication are available from the clinical trial sponsor on the basis of a data sharing agreement on reasonable request. The data are not publicly available due to them containing information that could compromise research par-ticipant privacy and consent. The study protocol is available in the supplementary material. Corre-spondance should be submitted to Dr. Rolf Fendel (rolf.fendel@uni-tuebingen.de).

Field-specific reporting

Please select the one below that is the best fit for your research. If you are not sure, read the appropriate sections before making your selection.

☒ Life sciences ☐ Behavioural & social sciences ☐ Ecological, evolutionary & environmental sciences

For a reference copy of the document with all sections, see nature.com/documents/nr-reporting-summary-flat.pdf

Life sciences study design

All studies must disclose on these points even when the disclosure is negative.

Sample size	To detect infection rates of 25% or less in the vaccination and 85% in the placebo groups (allocated in a 2:1 ratio) with a power of 80% and a two-tailed alpha of 5%, 14 PfSPZ-CVac immunized and 7 placebo-treated volunteers were required. Sample size was calculated in R using the nBinomial function provided in the gsDesign package.
Data exclusions	No data was excluded.
Replication	Sample size for biological replicated in vaccine efficiency was described above - 20 individuals were recruited into the study. Two individuals withdrew consent during the trial. 13 volunteers received full vaccine regimen and 5 received placebo.<br>RT-qPCR was performed as a single replicate in technical triplicates to determine respective Ct values. All preocedures were performed according to the MIQE guidelines (Bustin et al. 2009). Immunogenicity (ELISA assay) was determined in three technical replicates. Microarrays were measured in single measurements, including positive and negative controls in each slide to control for assay reproducibility. Thick blood smears were evaluated by two independent microscopists, and in case of discrepancy, a third microscopists read the slides and for positivity or negativity of the thick blood smear.
Randomization	Randomization was performed on the day of first immunization prior to injection by a third party outside the study team and sponsor. The randomization list was generated using a random number generator (Mersenne-Twister implemented in R; www.r-project.org) and given to a dedicated member of the formulation team who did not have a further role in the trial. The allocation ratio for PfSPZ Challenge to placebo was 2:1.
Blinding	Investigators (clinical team, sponsor) and volunteers remained blinded during the study and the data analysis. First unblinding was done following an interim database lock after Day 56 of CHMI to allow assessment of VE by an in-dependent statistician.

Reporting for specific materials, systems and methods

We require information from authors about some types of materials, experimental systems and methods used in many studies. Here, indicate whether each material, system or method listed is relevant to your study. If you are not sure if a list item applies to your research, read the appropriate section before selecting a response.

Materials & experimental systems

n/a	Involved in the study	
☐	☒	Antibodies
☒	☐	Eukaryotic cell lines
☒	☐	Palaeontology and archaeology
☒	☐	Animals and other organisms
☐	☒	Human research participants
☐	☒	Clinical data
☒	☐	Dual use research of concern

Methods

n/a	Involved in the study	
☒	☐	ChIP-seq
☒	☐	Flow cytometry
☒	☐	MRI-based neuroimaging

Antibodies

Antibodies used	HRP conjugated goat anti-human IgG (Jackson ImmunoResearch, # 109-035-098) Lot: 141861<br>HRP-conjugated goat anti-human IgM (ImmunoReagents, # GtxHu-006-E2HRPX) Lot: 23-128-022312<br>goat anti-human IgG QDot™800, Grace Bio-Labs #110635 Lot: 137277A<br>biotin-SP-conjugated goat anti-human IgM, Jackson ImmunoResearch #109-065-043 Lot: 152173
Validation	Only commercially available secondary antibodies were used.

Human research participants

Policy information about studies involving human research participants

Population characteristics	male (number, percentage) 6 (46%) 5 (71%)<br>female (number, percentage) 7 (54%) 2 (29%)<br>age in years (median, range) 25 (19-42) 26 (21-36)<br>height in cm (median, range) 171 (159-184) 178 (165-188)<br>weight in kg (median, range) 69 (50-100) 73 (51-86)<br>BMI in kg/m2 (median, range) 23.8 (16.9-33.8) 23.2 (18.7-25.6)
Recruitment	Healthy, malaria-naive volunteers aged 18 to 45 were recruited in Tübingen and surrounding areas. All participants provided written informed consent at the screening visit. Female volunteers were required to practice continuous effective birth control during the study period. For safety reasons, volunteers were required to be reachable 24/7 by mobile phone. Prior to enrollment, participants had to pass a questionnaire assessing the understanding of risks and obligations of the trial. Main exclusion criteria were history of malaria or previous participation in a malaria vaccine trial and any relevant medical history. The full list of eligibility criteria is listed in the protocol which is available as a supplementary material.<br>Volunteer selection bias is not expected during this procedure, as recruitment criteria were objectively perdetermined and depended on general health condition and questionnaire outcome. Any further selection bias was minimized through randomization to the interventional study treatment or placebo, and double-blinding of volunteer and clinical and diagnostic team.
Ethics oversight	The trial was approved by the Paul Ehrlich Institut and the Clinical Ethics Committee at the University Hospital of Tübingen (UKT). The study design and conduct complied with all relevant regulations regarding the use of human study participants and was conducted in accordance to the criteria set by the Declaration of Helsinki. The study was performed in accordance with Good Clinical Practice/International Conference on Harmonisation guidelines.

Note that full information on the approval of the study protocol must also be provided in the manuscript.

Clinical data

Policy information about clinical studies

All manuscripts should comply with the ICMJEguidelines for publication of clinical research and a completedCONSORT checklist must be included with all submissions.

Clinical trial registration	The trial is registered in the European Union Clinical Trials Register (EudraCT-Nr: 2018-004523-36).
Study protocol	Supplementary information
Data collection	This single-center, double-blinded, randomized trial was conducted at the Institute of Tropical Medicine, University of Tübingen, Tübingen, Germany, from April 2019. Planned trial close out was November 2020, but this had to be postponed due to the COVID-19 pandemic. The first CHMI was performed in September 2019 with a follow-up until November 2019.
Outcomes	The primary objective was to establish an immunization regimen of three injections of PfSPZ Challenge by DVI and oral chloroquine chemoprophylaxis administered on Days 0, 5 and 28, that is safe and well tolerated. The secondary objective was to establish such an immunization regimen, that provides protection against repeat CHMI in healthy adult subjects, as assessed by RT-qPCR after challenge. The aim of the trial was to assess safety, tolerability and efficacy of a condensed immunization regimen with three doses of PfSPZ Challenge and CQ. The primary VE endpoint was the proportion of protected volunteers. Protection was defined as the absence of parasitemia in the peripheral blood for 28 days after CHMI. According to the study protocol, parasitemia was defined as at least one RT-qPCR result above 100 parasites per ml among three positive results, at least 12 hours apart or as a positive thick blood smear. The primary safety endpoint was the occurrence of related grade 3 and 4 adverse events following the first CQ administration until the end of the trial. Related adverse events were recorded and reported using the terminology defined in the Medical Dictionary for Regulatory Activities (MedDRA). Further exploratory endpoints were time to parasitemia (defined as the time to the first positive RT-qPCR result among three positive results at least 12 hours apart, with at least one of them being above 100 parasites per ml, or the time to a positive thick blood smear) and the characterization of immune responses including the identification of parasitological and immunological correlates of protection against CHMI.
